# An annotated checklist of the chrysidid wasps (Hymenoptera, Chrysididae) from China

**DOI:** 10.3897/zookeys.455.6557

**Published:** 2014-11-19

**Authors:** Paolo Rosa, Na-sen Wei, Zai-fu Xu

**Affiliations:** 1 Via Belvedere 8/d, I-20881 Bernareggio (MB), Italy; 2 Department of Entomology, College of Natural Resources and Environment, South China Agricultural University, Guangzhou 510640, China

**Keywords:** Chrysididae, catalogue, new combination, status revived, pictures, China

## Abstract

An annotated checklist of the Chinese Chrysididae is provided. The list includes 188 species and subspecies in twenty three genera of five subfamilies. Four species are proposed as new combinations: *Hedychridium cupreum asianum* (Linsenmaier, 1997), *Philoctetes deauratus* (Mocsáry, 1914), *Philoctetes mordvilkoi* (Semenov-Tian-Shanskij, 1932), and *Pseudomalus hypocritus* (du Buysson, 1893). Two species are revalidated: *Chrysis consobrina* Mocsáry, 1889, and *Philoctetes mongolicus* (du Buysson, 1901). Historical data with comments on the current taxonomic position, and the pictures of sixty five types are also given.

## Introduction

The Chrysididae, commonly known as cuckoo wasps or goldwasps, are a cosmopolitan family and have the greatest diversity in the Palaearctic Region (Morgan 1984). Based on the most recent investigation, 87 genera and 2509 species have been described worldwide in this family (Aguiar et al. 2013). Cuckoo wasps are parasitoids or cleptoparasites of stick insects, moths, and other wasps, bees and sawflies (Kimsey and Bohart 1991). Kimsey and Bohart (1991) divided this family into four subfamilies (Amiseginae, Chrysidinae, Cleptinae, and Loboscelidinae), while Mocsáry (1889), Linsenmaier (1959), Mingo (1994), and Rosa et al. (2013) considered also Parnopinae as a valid subfamily and we follow the latter taxonomic interpretation.

In 1864 Smith included *Stilbum cyanurum* (sub *splendidum*) from China in a table of his paper. This is the first report of the Chrysididae from China. From then on, many articles with occasional descriptions of new species of Chinese chrysidid can be found in the literature (Smith 1874a, 1874b; du Buysson 1887, 1893, 1898a, 1898b, 1898c, 1899, 1900, 1901a, 1904, 1908; Mocsáry 1887, 1889, 1890, 1911, 1912a, 1912b, 1913a, 1913b, 1914; Radoszkowski 1887, 1889, 1891; Semenow 1892; Semenov-Tian-Shansky 1909; Bischoff 1910; Uchida 1927; Semenov-Tian-Shanskij 1932, 1954, 1967; Hammer 1936, 1950; Tsuneki 1947, 1948a, 1950, 1953a, 1970b, 1982; Linsenmaier 1959, 1968, 1997a; Lin 1964; Móczár 1968; Kimsey 1987a, 1987b, 1988, 1995; Rosa 2003; Xu et al. 2003, 2006; Liu et al. 2010, 2011; Yao et al. 2010; Wei et al. 2013; Wei et al. 2014a, 2014b). But these descriptions are scattered and a compositive review of the Chinese Chrysididae is necessary.

The aim of the present checklist is to summarize the taxa previously recorded for China as a base for further research.

## Material and methods

The list follows the genera subdivision proposed by Kimsey and Bohart (1991), with few exceptions. The species are listed alphabetically. Type depositories are given mainly according to Kimsey and Bohart (1991). Types examined are asterisked (*) after the type depositories.

The following abbreviations are used in the text: aberr. (aberratio), biol. (biology), cat. (catalogue), cit. (citation), comp. notes (comparative notes), ecol. (ecology), design. (designation), distr. (distribution), ex. (examplar), fig. (figs) (figure (figures)), misid. (misidentification), pl. (pls) (fig (figs)), syn. (synonym), tab. (table), tax. (taxonomy), typ. gen. (typus generis).

Pictures of the types were taken with Nikon D-80 connected to the stereomicroscope Togal SCZ and stacked with the software Combine ZP.

Types and other specimens were deposited in the following institutions:

AEI The American Entomological Institute, Gainesville, Florida, USA.

BMNH The Natural History Museum, London, England.

CNC Hymenoptera Section, Biosystematics Institute, Ottawa, Canada.

EIHU Entomology Institute, Hokkaido University, Hokkaido, Japan.

HNHM Hungarian Naturwissenschaftlichen Museum, Budapest, Hungary.

HEC Hope Entomological Collections, Oxford University Museum, England.

HUSK Department of Biological Sciences, Faculty of Science, Hanseo University, Seosan, Korea Republic.

ISEA-PAS Invertebrate collections of the Institute of Systematics and Evolution of Animals, Polish Academy of Sciences in Krakow, Poland.

KUM The Kyushu University Museum, Faculty of Bioresource and Bioenvironmental Sciences and Faculty of Social and Cultural Studies, Fukuoka, Japan.

LSL Linnean Society of London, England.

LZM Lund Zoological Museum, University of Lund, Sweden.

MHNG Muséum d’Histoire Naturelle, Genève, Switzerland.

MMU Zoologial Museum, Lomonosov State University Moscow, Russia.

MNHN Muséum National d’Histoire Naturelle, Paris, France.

MNHU Museum für Naturkunde der Humboldt-Universität, Berlin, Germany.

MRSN Museo Regionale di Scienze Naturali, Turin, Italy.

MSNG Museo Civico di Storia Naturale “G. Doria”, Genoa, Italy.

MSNM Museo Civico di Storia Naturale di Milano, Italy.

NHMW Naturhistorisches Museum Wien, Vienna, Austria.

NHRS Swedish Museum of Natural History, Stockholm, Sweden.

NIAS Laboratory of Insect Systematics, National Institute of Agro-Environmental Sciences, Kannondai, Tsukuba, Ibaraki, Japan.

NMLS Natur Museum Luzern, Switzerland.

OMNH Osaka Museum of Natural History, Osaka, Japan.

RMNH Nationaal Natuurhistorisch Museum, Leiden, The Netherlands.

SCAU Department of Entomology, College of Natural Resources and Environment, South China Agricultural University, Guangzhou, China.

SMFD Forschungsinstitut und Museum Senckenberg, Frankfurt am Main, Germany.

TARI Entomology Collection, Taiwan Agricoltural Research Institue, Taichung, Taiwan, China.

USNM United States National Museum of Natural History, United States National Entomological Collection, Washington DC, USA.

ZIN Zoological Institute, St. Petersburg, Russia.

ZJUH Institute of Insect Sciences, University of Zhejiang, Hangzhou, China.

ZMU Zoological Museum, University of Copenhagen, Denmark.

## Results

### I. Taxa from China

#### Subfamily Cleptinae

##### 1. Genus *Cleptes* Latreille, 1802

###### 1. Cleptes albonotatus

Taxon classificationAnimaliaHymenopteraChrysididae

Wei, Rosa & Xu, 2013

http://species-id.net/wiki/Cleptes_albonotatus

Cleptes albonotatus Wei, Rosa & Xu, 2013: 61. Holotype ♀, China: Guangdong: Nanling National Nature Reserve (59 (key), 61 (descr.), 62 (pl. 1), 63 (*satoi* group), 65 (comp. notes), 74 (comp. notes), 75 (comp. notes), depository: SCAU)*.

####### Distribution.

China (Guangdong).

###### 2. Cleptes asianus

Taxon classificationAnimaliaHymenopteraChrysididae

Kimsey, 1987

http://species-id.net/wiki/Cleptes_asianus

Cleptes asianus Kimsey, 1987b: 56. Holotype ♀, Taiwan: Wushe (56 (descr.), 57 (figs 3, 4, 7), depository: AEI).
Cleptes asianus : Kimsey and Bohart 1991: 59 (cat., *orientalis* group); Wei et al. 2013: 56 (tab.), 58 (key), 60 (tax., *asianus* group).
Cleptes (Cleptes) asianus : Móczár 2000: 320 (diagnosis of the *asianus* group; cat.); 322 (key; figs 3–5); 325 (tax., descr.).

####### Distribution.

China (Taiwan).

####### Remarks.

Móczár (2000) added some morphological characteristics to the original description.

###### 3. Cleptes eburnecoxis

Taxon classificationAnimaliaHymenopteraChrysididae

Wei, Rosa & Xu, 2013

http://species-id.net/wiki/Cleptes_eburnecoxis

Cleptes eburnecoxis Wei, Rosa & Xu, 2013: 63. Holotype ♂, China: Zhejiang: Mt. Tianmu, Xianrending (60 (key), 63 (type series: China: Zhejiang: Mt. Tianmu, Xianrending; Guangxi: Longsheng, Huaping National Nature Reserve, descr.), 64 (pl. 2), 65 (*townesi* group), depository: SCAU)*.

####### Distribution.

China (Zhejiang, Guangxi).

###### 4. Cleptes flavolineatus

Taxon classificationAnimaliaHymenopteraChrysididae

Wei, Rosa & Xu, 2013

http://species-id.net/wiki/Cleptes_flavolineatus

Cleptes flavolineatus Wei, Rosa & Xu, 2013: 65. Holotype ♀, China: Zhejiang: Mt. Tianmu, Xianrending (59 (key), 61 (comp. notes), 65 (descr.), 66 (pl. 3), 67 (*satoi* group), 74 (comp. notes), depository: ZJUH)*.

####### Distribution.

China (Zhejiang).

###### 5. Cleptes helanshanus

Taxon classificationAnimaliaHymenopteraChrysididae

Wei, Rosa & Xu, 2013

http://species-id.net/wiki/Cleptes_helanshanus

Cleptes helanshanus Wei, Rosa & Xu, 2013: 67. Holotype ♀, China: Inner Mongolia: Mt. Helan (59 (key), 67 (descr.), 68 (pl. 4), 69 (*nitidulus* group), depository: SCAU)*.

####### Distribution.

China (Inner Mongolia).

###### 6. Cleptes mandsuricus

Taxon classificationAnimaliaHymenopteraChrysididae

Móczár, 1968

http://species-id.net/wiki/Cleptes_mandsuricus

Plate 1

Cleptes (Holcocleptes) mandsuricus Móczár, 1968: 171. Holotype ♂, China: Manchuria: Erzendjanzsy (171 (descr.), 172 (figs 5–7), depository: NHMW)*.
Cleptes (Holcocleptes) mandsuricus : Móczár 1998: 325 (cat., aerosus group), 329 (key), 332 (figs 12–13), 337 (China: Manchuria: Erzendjanzsy, tax., descr.).
Cleptes mandsuricus : Kimsey and Bohart 1991: 61 (China: Manchuria: Erzendjanzsy, cat., *aerosus* group); Kurzenko and Lelej 2007: 1002 (Northeast China, cat.); Wei et al. 2013: 56 (tab.), 60 (key), 69 (Northeast China, tax.).

####### Distribution.

China.

####### Remarks.

Móczár (1998) added some morphological characteristics to the original description.

###### 7. Cleptes mareki

Taxon classificationAnimaliaHymenopteraChrysididae

Rosa, 2003

http://species-id.net/wiki/Cleptes_mareki

Cleptes (Leiocleptes) mareki Rosa, 2003: 408. Holotype ♀, China: Shanxi: Zhongtiao Shan c. [= Mt. Zhongtiao], 45 km W of Sanmenxia (408 (descr.), 410 (comp. tab.), 411 (figs 1, 2), 412 (figs 3, 4), 413 (figs 5–6), depository: MSNM)*.
Cleptes (Leiocleptes) mareki : Rosa 2005: 5 (cit.), 6 (cit), 7 (China: Shanxi: Zhongtiao Shan c. [= Mt. Zhongtiao], 45 Km W of Sanmenxia, cat.).
Cleptes mareki : Wei et al. 2013: 56 (tab.), 59 (key), 60 (key), 68 (comp. notes), 70 (China: Shanxi; Gansu: Jiuquan, Huangnibao, tax., *nitidulus* group), 71 (pl. 5, ♂), 72 (pl. 6, ♀), 82 (comp. notes).

####### Distribution.

China (Gansu, Shanxi).

####### Remarks.

Wei et al. (2013) added some morphological characteristics to the original description.

###### 8. Cleptes metallicorpus

Taxon classificationAnimaliaHymenopteraChrysididae

Ha, Lee & Kim, 2011

http://species-id.net/wiki/Cleptes_metallicorpus

Cleptes metallicorpus Ha, Lee & Kim, 2011: 493. Holotype ♀, Korea: Gangwon-do, Wonju-si, Maeji-ri (489 (cit.), 490 (key; figs 1B, 1H, 1O, 1S), 491 (figs 2D, 2H), 493 (descr.), depository: HUSK).
Cleptes metallicorpus : Wei et al. 2013: 59 (key), 70 (China: Guangdong: Nanling National Nature Reserve; Zhejiang: Mt. Tianmu, Xianrending, Mt. Tianmu, Qiliting; Shaanxi: Quing Ling Shan mts, tax., descr.), 73 (pl. 7 ♀), 74 (*asianus* group), 87 (comp. notes), 91 (comp. notes).

####### Distribution.

China (Shaanxi, Zhejiang, Guangdong). Korea (Ha et al. 2011).

###### 9. Cleptes niger

Taxon classificationAnimaliaHymenopteraChrysididae

Wei, Rosa & Xu, 2013

http://species-id.net/wiki/Cleptes_niger

Cleptes niger Wei, Rosa & Xu, 2013: 74. Holotype ♀, China: Shaanxi: Mt. Taibai, 1100 m (59 (key), 61 (comp. notes), 65 (comp. notes), 74 (descr.), 75 (pl. 8), 76 (*satoi* group), depository: SCAU)*.

####### Distribution.

China (Shaanxi).

###### 10. Cleptes seoulensis

Taxon classificationAnimaliaHymenopteraChrysididae

Tsuneki, 1959

http://species-id.net/wiki/Cleptes_seoulensis

Cleptes seoulensis Tsuneki, 1959: 13. Holotype ♀, Korea: Keijo (4 (comp. tab.), 6 (key), 13 (descr.), 14 (comp. notes), 17 (figs 8–12), depository: OMNH, not NIAS (Móczár 1998: 340)).
Cleptes seoulensis : Kim 1970: 506 (tax.); Kimsey and Bohart 1991: 64 (cat., *orientalis* group); Ha et al. 2011: 489 (cit.), 490 (key; figs 1C, 1F, 1M, 1T), 491 (figs 2B, 2F), 492 (descr.); Wei et al. 2013: 58 (key), 59 (key), 76 (tax., *fudzi* group), 77 (descr., pl. 9, ♂), 78 (*fudzi* group).
Cleptes (Holcocleptes) seoulensis : Móczár 1998: 325 (*fudzi* group, cat.), 330 (fig. 9), 331 (key), 332 (figs 14–16), 340 (descr., tax.).

####### Distribution.

China (Anhui). Korea (Tsuneki 1959; Kim 1970).

####### Remarks.

Móczár (1998) added some morphological characteristics to the original description. Wei et al. (2013) gave the first description of the male.

###### 11. Cleptes shengi

Taxon classificationAnimaliaHymenopteraChrysididae

Wei, Rosa & Xu, 2013

http://species-id.net/wiki/Cleptes_shengi

Cleptes semiauratus (Linnaeus, 1758): Sheng et al. 1998: 7 (misid.).
Cleptes shengi Wei, Rosa & Xu, 2013: 78. Holotype ♀, China: Jilin: Maoershan National Forest Park (59 (key), 78 (descr.), 79 (pl. 10), 80 (*semiauratus* group), 93 (comp. notes), depository: SCAU)*.

####### Distribution.

China (Jilin).

###### 12. Cleptes sinensis

Taxon classificationAnimaliaHymenopteraChrysididae

Wei, Rosa & Xu, 2013

http://species-id.net/wiki/Cleptes_sinensis

Cleptes sinensis Wei, Rosa & Xu, 2013: 81. Holotype ♂, China: Shaanxi: Liping National Forest Park (60 (key), 80 (Type series: China: Shaanxi: Liping National Forest Park; Shaanxi, Mt. Taibai; Shaanxi: Liping National; Shaanxi, Liuba, Mt. Zibo; Hainan: Jianfengling National Nature Reserve; Sichuan: Wolong National Nature Reserve; Hubei: Wufeng, Houhe National Nature Reserve, descr.), 81 (pl. 11, ♂), 83 (*nitidulus* group), depository: SCAU)*.

####### Distribution.

China (Shaanxi, Zhejiang, Hubei, Hainan, Sichuan).

###### 13. Cleptes sjostedti

Taxon classificationAnimaliaHymenopteraChrysididae

Hammer, 1950

http://species-id.net/wiki/Cleptes_sjostedti

Plate 2

Cleptes sjostedti *Cleptes Sjöstedti* Hammer, 1950: 2. Holotype ♀, China: Kiangsu [= Jiangsu], (2 (descr.), depository: NHRS)*.
Cleptes pinicola Lin, 1959: 205. Holotype ♀, Taiwan: Lien-hwa-chich (205 (descr.), depository: TARI) (synonymised by Móczár 1998).
Cleptes sjostedti : Kimsey and Bohart 1991: 64 (China: Kiangsu [= Jiangsu], cat., *orientalis* group); Kurzenko and Lelej 2007: 1002 (Jiangsu, cat.); Wei et al. 2013: 56 (tab.), 58 (key), 60 (key), 83 (China: Hunan, Liuyang City; Yunnan: Xiangyun; Zhejiang, Anji; Guangdong: Xinhui; Yunnan, Kunming; Anhui: Ningguo; Zhejiang, Gaozhou, Bamen, tax., descr.), 84 (pl. 12, ♀), 85 (pl. 13, ♂), 86 (*fudzi* group).
Cleptes (Holcocleptes) sjoestedti (!): Móczár 1998: 325 (cat., *fudzi* group), 332 (key, fig. 19), 340 (tax.), 341 (descr.).

####### Distribution.

China (Jiangsu, Zhejiang, Anhui, Taiwan, Hunan, Guangdong, Yunnan). Korea (Móczár 1998).

####### Host.

*Nesodiprion japonica* Marlatt (Hymenoptera, Diprionidae) (Lin 1959; Móczár 1998).

####### Remarks.

Móczár (1998) designated the neotype based on the paratype of *sjostedti* deposited in NHMW. A closer examination of the type material (by Paolo Rosa) has revealed the original holotype in NHRS, thus Móczár’s neotype has consequently been set aside. *Cleptes pinicola* Lin was not listed in Kimsey and Bohart (1991). Móczár (1998) and Wei et al. (2013) have provided detailed descriptions.

###### 14. Cleptes taiwanus

Taxon classificationAnimaliaHymenopteraChrysididae

Tsuneki, 1982

http://species-id.net/wiki/Cleptes_taiwanus

Cleptes taiwanus Tsuneki, 1982: 2. Holotype ♀, Taiwan: Pempuchi (2 (descr.), depository: OMNH).
Cleptes taiwanus : Móczár 2000: 320 (cat., *asianus* group), 321 (fig. 2), 322 (key, fig. 6), 328 (comp. notes), 329 (Taiwan: Nantou Prov., Pempuchi, tax.); Wei et al. 2013: 56 (tab.), 59 (key), 86 (tax., *asianus* group), 91 (comp. notes).

####### Distribution.

China (Taiwan).

####### Remarks.

*Cleptes taiwanus* Tsuneki is not listed in Kimsey and Bohart (1991). Móczár (2000) has provided some additions and corrections to the original description.

###### 15. Cleptes tibetensis

Taxon classificationAnimaliaHymenopteraChrysididae

Wei, Rosa & Xu, 2013

http://species-id.net/wiki/Cleptes_tibetensis

Cleptes tibetensis Wei, Rosa & Xu, 2013: 87. Holotype ♂, Tibet: Pailongxiang, Daxiagu (60 (key), 87 (descr.), 88 (pl. 14), 89 (*asianus* group), 91 (comp. notes), depository: SCAU)*.

####### Distribution.

China (Tibet).

###### 16. Cleptes townesi

Taxon classificationAnimaliaHymenopteraChrysididae

Kimsey, 1987

http://species-id.net/wiki/Cleptes_townesi

Cleptes townesi Kimsey, 1987b: 58. Holotype ♂, Taiwan: Wushe (58 (descr., figs 2–6), depository: AEI).
Cleptes townesi : Kimsey and Bohart 1991: 58 (tax.), 64 (Taiwan: Wushe, cat., *townesi* group); Móczár 2000: 330 (Taiwan: Wu-feng, tax., descr., *townesi* group); Wei et al. 2013: 56 (tab.), 60 (key), 63 (comp. notes), 89 (China: Zhejiang: Hangzhou; Fujian: Chong’an, Mt. Wuyi, Jiuqu, descr., *townesi* group), 90 (pl. 15, ♂).

####### Distribution.

China (Zhejiang, Fujian, Taiwan).

###### 17. Cleptes villosus

Taxon classificationAnimaliaHymenopteraChrysididae

Wei, Rosa & Xu, 2013

http://species-id.net/wiki/Cleptes_villosus

Cleptes villosus Wei, Rosa & Xu, 2013: 91. Holotype ♂, China: Guizhou, Suiyang, Kuankuoshui National Nature Reserve (60 (key), 87 (comp. notes), 91 (type series: China: Guizhou: Suiyang, Kuankuoshui National Nature Reserve; Daozhen, Dashahe, Xiannvdong, descr.), 92 (pl. 16), 93 (*asianus* group), depository: SCAU)*.

####### Distribution.

China (Guizhou).

#### Subfamily Amiseginae

##### 2. Genus *Magdalium* Kimsey, 1986

###### 18. Magdalium orchidense

Taxon classificationAnimaliaHymenopteraChrysididae

Kimsey, 1995

http://species-id.net/wiki/Magdalium_orchidense

Magdalium orchidense Kimsey, 1995: 594. Holotype ♂, Taiwan: Orchid Isl., Batel Tobago (591 (fig. 6), 594 (comp. notes, descr.), depository: CNC).

####### Distribution.

China (Taiwan).

##### 3. Genus *Nipponosega* Kurzenko & Lelej, 1994

###### 19. Nipponosega kurzenkoi

Taxon classificationAnimaliaHymenopteraChrysididae

Xu, He & Terayama, 2003

http://species-id.net/wiki/Nipponosega_kurzenkoi

Nipponosega kurzenkoi Xu, He & Terayama, 2003: 195. Holotype ♀, China: Zhejiang: Suichang, Mt. Jiulongshan (195 (key, descr.), 196 (figs 1, 2), depository: SCAU)*.
Nipponosega kurzenkoi : Kurzenko and Lelej 2007: 1002 (China: Zhejiang, cat.).

####### Distribution.

China (Zhejiang).

#### Subfamily Loboscelidiinae

##### 4. Genus *Loboscelidia* Westwood, 1874

###### 20. Loboscelidia guangxiensis

Taxon classificationAnimaliaHymenopteraChrysididae

Xu, Weng & He, 2006

http://species-id.net/wiki/Loboscelidia_guangxiensis

Loboscelidia guangxiensis Xu, Weng & He, 2006: 208. Holotype ♂, China: Guangxi: Jiuwandashan (208 (descr.), 209 (key, figs 1–6), depository: SCAU)*.
Loboscelidia guangxiensis : Liu et al. 2010: 641 (key in Chinese), 645 (key in English); Yao et al. 2010: 526 (China: Guangxi: Jiuwandashan; Guangdong: Nanling National Nature Reserve; Chebaling National Nature Reserve, distr., tax.), 527 (figs 1A–1H), 528 (comp. notes), 533 (key); Kimsey 2012: 6 (key), 16 (comp. notes), 18 (China: Guangxi, Jiuwandashan, tax.), 19 (comp. notes).

####### Distribution.

China (Guangdong, Guangxi).

###### 21. Loboscelidia hei

Taxon classificationAnimaliaHymenopteraChrysididae

Liu, Yao & Xu, 2010

http://species-id.net/wiki/Loboscelidia_hei

Loboscelidia hei Liu, Yao & Xu, 2010: 642. Holotype ♀, China: Fujian, Mt. Meihua (641 (key in Chinese), 642 (descr. in Chinese, figs 1–6), 645 (key and descr. in English), depository: SCAU)*.

####### Distribution.

China (Fujian).

###### 22. Loboscelidia levigata

Taxon classificationAnimaliaHymenopteraChrysididae

Yao, Liu & Xu, 2010

http://species-id.net/wiki/Loboscelidia_levigata

Loboscelidia levigata Yao, Liu & Xu, 2010: 528. Holotype ♂, China: Guangdong: Chebaling National Nature Reserve (526 (cit.), 528 (type series: China: Guangdong: Chebaling National Nature Reserve, Nanling National Nature Reserve; Fujian: Minqing County, Huangchulin Provincial Nature Reserve, descr.), 529 (figs 2A–2H), 533 (key), depository: SCAU)*.
Loboscelidia levigata : Kimsey 2012: 8 (key), 24 (China: Guandong, tax.), 30 (comp. notes), 35 (comp. notes).

####### Distribution.

China (Fujian, Guangdong).

###### 23. Loboscelidia maai

Taxon classificationAnimaliaHymenopteraChrysididae

(Lin, 1964)

http://species-id.net/wiki/Loboscelidia_maai

Scelidoloba maai Lin, 1964: 238. Holotype ♀ (not ♂); Taiwan: Paomingszu, 2 km S Keelung (238 (descr.), depository: TARI).
Loboscelidia latigena Lin, 1964: 241. Holotype ♂, Taiwan: Tsaoshan, 20 km NW Taipei city (241 (descr.), depository: TARI) (synonymised by Kimsey 2012).
Loboscelidia artigena Lin, 1964: 243. Holotype ♂, Taiwan: Paomingzu, 2 Km S Keelung (243 (descry.), depository: TARI) (synonymised by Kimsey and Bohart 1991).
Loboscelidia maai (Lin, 1964): Kimsey and Bohart 1991: 147 (Taiwan, cat.); Xu et al. 2006: 209 (key); Liu et al. 2010: 641 (key in Chinese), 643 (comp. notes in Chinese.), 645 (key and comp. notes in English); Yao et al. 2010: 526 (cit.), 533 (key); Kimsey 2012: 11 (comp. notes), 24 (Taiwan, tax.).

####### Distribution.

China (Taiwan).

####### Remarks.

In Kimsey (2012) *Loboscelidia latigena* is considered a synonym of *Loboscelidia maai*, but in the keys *Loboscelidia latigena*, rather than *Loboscelidia maai*, is mentioned.

###### 24. Loboscelidia sinensis

Taxon classificationAnimaliaHymenopteraChrysididae

Kimsey, 1988

http://species-id.net/wiki/Loboscelidia_sinensis

Loboscelidia sinensis Kimsey, 1988: 76. Holotype ♂, China: Hainan (76 (descr.), depository: BMNH).
Loboscelidia sinensis : Kimsey and Bohart 1991: 148 (China: Hainan, cat.); Xu et al. 2006: 209 (key); Liu et al. 2010: 641 (key in Chinese), 645 (key in English); Yao et al. 2010: 526 (cit.), 530 (China: China: Zhejiang: Kaihua County, Gutianshan Provincial Nature Reserve; Taishun County, Wuyanling Provincial Nature Reserve; Fujian, Minqing, Huangchulin Provincial Nature Reserve; Guangdong, Nanling National Nature Reserve; Shixing, Chebaling National Nature Reserve; Hainan, Jianfengling National Nature Reserve; Bawangling National Nature Reserve, descr., tax.), 531 (figs 3A–3H), 533 (key, comp. notes); Kimsey 2012: 8 (key), 24 (comp. notes), 37 (China: Hainan, descr., tax.), 39 (cit.).

####### Distribution.

China (Zhejiang, Fujian, Guangdong, Hainan).

###### 25. Loboscelidia striolata

Taxon classificationAnimaliaHymenopteraChrysididae

Yao, Liu & Xu, 2010

http://species-id.net/wiki/Loboscelidia_striolata

Loboscelidia striolata Yao, Liu & Xu, 2010: 530. Holotype ♂, China: Guangdong: Nanling National Nature Reserve (526 (cit.), 530 (descr.), 532 (figs 4A–4H), 533 (key), depository: SCAU)*.
Loboscelidia striolata : Kimsey 2012: 24 (comp. notes), 38 (China: Guangdong, tax.), 39 (comp. notes).

####### Distribution.

China (Guandong).

###### 26. Loboscelidia zengae

Taxon classificationAnimaliaHymenopteraChrysididae

Liu, Yao & Xu, 2010

http://species-id.net/wiki/Loboscelidia_zengae

Loboscelidia zengae Liu, Yao & Xu, 2010: 643. Holotype ♀, China: Hainan: Wuzhishan (641 (key in Chinese), 643 (descr. in Chinese), 644 (figs 7–12), 645 (key, descr. in English), depository: SCAU)*.

####### Distribution.

China (Hainan).

##### 5. Genus *Rhadinoscelidia* Kimsey, 1988

###### 27. Rhadinoscelidia delta

Taxon classificationAnimaliaHymenopteraChrysididae

Liu, Yao & Xu, 2011

http://species-id.net/wiki/Rhadinoscelidia_delta

Rhadinoscelidia delta Liu, Yao & Xu, 2011: 13. Holotype ♀, China: Hainan: Wuzhishan (13 (descr.), 14 (figs 1–6), 15 (figs 7–12), 16 (key), depository: SCAU)*.

####### Distribution.

China (Hainan).

#### Subfamily Chrysidinae

##### Tribe Elampini

###### 6. Genus *Elampus* Spinola, 1806

####### 28. Elampus albipennis

Taxon classificationAnimaliaHymenopteraChrysididae

(Mocsáry, 1889)

http://species-id.net/wiki/Ellampus_albipennis

Plate 3

Ellampus (Notozus) albipennis Mocsáry, 1889: 80 [*nec Elampus*]. Lectotype ♂ design. by Móczár (1964b: 447), Russia: Sarepta (depository: HNHM)*.
Notozus violascens (Mocsáry, 1889): du Buysson 1911: 218 (China: “Nan Chan, versant Nord; route de Cha Tchéou à Kan Tchéou; Linchouei, par 1, 500 mètres d’altitude, 25 juin 1908. Nan Chan: route de Kan Tchéou à Lan Tchéou par Si Ning; col de King Yang Ling, par 3,800 mètres d’altitude”, tax.) [misid.].
Notozus albipennis : Tsuneki 1953a: 54 (Manchuria: Kaiyüan, Kupeikau, tax.).
Omalus (Notozus) albipennis : Linsenmaier 1959: 16 (key), 24 (tax., cat., distr.).
Elampus albipennis : Kimsey and Bohart 1991: 166 (cat.).

######## Distribution.

China (Liaoning, Beijing). Widely distributed in the Palaearctic Region (Tsuneki 1953a; Linsenmaier 1959). The Chinese specimen is not listed in the distribution by Kimsey and Bohart (1991).

######## Remarks.

The record of *Notozus violascens* Mocsáry by du Buysson (1911) is a misidentification: the reduced dimension and the green colour reported by the author match other species: *Elampus albipennis* Mocsáry, *Elampus mocsaryi* (Radoszkowski) and *Elampus turcmenicus* (Linsenmaier). Without examining the specimens it is not possible to correctly identify them.

Linsenmaier (1959) placed *Elampus tournieri* Dalla Torre, 1892 (repl. name for *Elampus viridis* Tournier, 1890) as synonym of *Elampus albipennis* (Mocsáry).

####### 29. Elampus bischoffi

Taxon classificationAnimaliaHymenopteraChrysididae

Kimsey, 1991

http://species-id.net/wiki/Elampus_bischoffi

Notuzus spinosus Bischoff, 1910: 436. Syntypes ♂♀, China: Chinese Turkestan [= Xinjiang], Tschakar and Saiback near Pulu (MNHU)*, *nec* Provancher, 1881.
Notuzus spinosus Bischoff, 1913: 7 (Chinese Turkestan [= Xinjiang], cat.).
Elampus (Notozus) spinosus : Linsenmaier 1959: 16 (key), 24 (Chinese Turkestan [= Xinjiang], tax., descr.).
Elampus bischoffi Kimsey (in Kimsey & Bohart), 1991: 167. Replacement name for *spinosus* Bischoff, 1910 *nec* Provancher, 1881, China: Sinkiang [= Xinjiang], cat.).
Elampus bischoffi : Kurzenko and Lelej 2007: 1003 (China: Inner Mongolia, Xinjiang, cat.).

######## Distribution.

China (Xinjiang, Inner Mongolia).

####### 30. Elampus coeruleus

Taxon classificationAnimaliaHymenopteraChrysididae

Dahlbom, 1854

http://species-id.net/wiki/Elampus_coeruleus

Plate 4

Elampus coeruleus Dahlbom, 1854: 46. Syntypes, ♂♀, Austria, Germany, Ukraine (46 (descr.), depository: MNHU)*.
Omalus viridiventris Abeille, 1878: 2. Unnecessary replacement name for *Elampus coeruleus* Dahlbom, 1854.
Notozus coeruleus f. coeruleus : Tsuneki 1953a: 53 (Manchuria: Tierin, cat.).
Omalus (Notozus) panzeri ssp. coeruleus : Linsenmaier 1968: 12 (Manchuria [Heilongjiang], distr.).
Notozus panzeri coeruleus : Banaszak 1980: 8 (Manchuria [Heilongjiang], tax.).
Elampus caeruleus (!): Kimsey and Bohart 1991: 167 (cat.).

######## Material examined.

Heilongjiang: 1♂, Harbin, without data; 1♂, Harbin, 10.VII.1949 leg. Alin (NMLS).

######## Distribution.

China (Heilongjiang, Liaoning). Widely distributed in the Palaearctic Region (Tsuneki 1953a; Linsenmaier 1959, 1968).

######## Remarks.

The taxonomic status of *Elampus coeruleus* is unclear. Various authors have alternately considered it as a valid species, a subspecies of *Elampus panzeri*, or a synonym of either *Elampus panzeri* or *Elampus constrictus*.

####### 31. Elampus constrictus

Taxon classificationAnimaliaHymenopteraChrysididae

(Förster, 1853)

http://species-id.net/wiki/Notozus_constrictus

Notozus constrictus Förster, 1853: 336. Holotype ♂, Germany: Aachen (336 (descr.), depository: MNHU)*.
Ellampus (Notozus) soror Mocsáry, 1889: 68. Neotype ♀ design. by Móczár (1964b: 422), Hungary: Budapest (depository: HNHM) (synonymised by Móczár, 1967).
Elampus panzeri sensu Trautmann, 1927, sensu Linsenmaier, 1959.
Notozus yasumatsui : Tsuneki 1948a: 116 (China: Kaiyüan).
Notozus panzeri : Tsuneki 1948a: 118 (China: Shanxi: Tungyehchen, distr., tax.), 128 (China: Shanxi, cat.).
Notozus coeruleus f. soror : Tsuneki 1953a: 54 (China: Kaiyüan, tax.).

######## Distribution.

China (Liaoning, Shanxi). Widely distributed in the Palaearctic Region (Linsenmaier 1959; Móczár 1967).

######## Remarks.

Tsuneki (1953a) wrote that the paratype of *Elampus yasumatsui* from Shanxi is to be referred to *Elampus* (=*Notozus*) *coeruleus* f. *soror*. The taxonomic position of *Elampus soror* is unclear: according to Linsenmaier (1959) it is a synonym of *Elampus coeruleus* Dahlbom. It has been considered a variety of *Elampus coeruleus* (Bischoff 1913), *Elampus panzeri* (Trautmann 1927) and *Elampus constrictus* (Móczár 1964b). Only Kimsey and Bohart (1991) listed *Elampus soror* as a valid species. We here consider *Elampus soror* as a synonym of *Elampus constrictus* following Móczár (1967) and Rosa and Soon (2012). Kimsey and Bohart (1991) considered *Elampus panzeri* and *Elampus constrictus* synonyms of Chrysis *Elampus scutellaris* Panzer, but without any type examination.

####### 32. Elampus mocsaryi

Taxon classificationAnimaliaHymenopteraChrysididae

Radoszkowski, 1887

http://species-id.net/wiki/Elampus_mocsaryi

Elampus mocsari (!) Radoszkowski, 1887: 45. Holotype ♀, China [not Mongolia]: Qinghai: Zaïdam (45 (descr.), depository: ISEA-PAS)*.
Ellampus (Notozus) mocsaryi : Mocsáry 1889: 80. Justified emendation of *Elampus mocsari* Radoszkowski, 1887.
Ellampus mocsaryi : Dalla Torre 1892: 14 (Mongolia [= China], cat.); Kimsey and Bohart 1991: 168 (Mongolia [= China], cat.).
Notozus mocsaryi : Bischoff 1913: 6 (Mongolia [= China], cat.).
Omalus (Notozus) mocsaryi : Linsenmaier 1959: 16 (key), 24 (Mongolia [= China], tax., descr.).

######## Distribution.

China (Qinghai).

####### 33. Elampus panzeri

Taxon classificationAnimaliaHymenopteraChrysididae

(Fabricius, 1804)

http://species-id.net/wiki/Chrysis_panzeri

Chrysis scutellaris Panzer, 1798: fig. 51, tav. 11. Holotype (sex unknown), Germany: Nurnberg (MNHU?).
Chrysis panzeri Fabricius, 1804: 172. Replacement name for *Chrysis scutellaris* Panzer, 1798, *nec* Fabricius, 1794.
Notozus constrictus Förster, 1853 sensu Linsenmaier 1959.
Notozus constrictus : Balthasar 1954: 73 (key), 77 (tax., descr.), 78 (China, distr.); Banaszak 1980: 8 (Manchuria, tax., biol.).
Omalus (Notozus) constrictus : Linsenmaier 1959: 16 (key), 24 (Manchuria, tax., descr.), 216 (fig. 667).

######## Material examined.

Heilongjiang: 1♂, Harbin, 19.VII.1953; 1♂, Harbin, 10.VII.1949 leg. Alin (NMLS).

######## Distribution.

China (Heilongjiang). Widely distributed in the Palaearctic Region (Linsenmaier 1959).

####### 34. Elampus schmidtianus

Taxon classificationAnimaliaHymenopteraChrysididae

(Semenov-Tian-Shanskij, 1967)

http://species-id.net/wiki/Notozus_schmidtianus

Plate 5

Notozus schmidtianus Semenov-Tian-Shanskij, 1967: 124. Holotype ♀, China: Xinjiang: Gashun Gobi, Sandzou oasis (124 (descr.), depository: ZIN)*.
Elampus schmidtianus : Kimsey and Bohart 1991: 170 (China: Gashun Gobi, cat.).

######## Distribution.

China (Xinjiang). Former southern USSR (Kimsey and Bohart 1991).

######## Remarks.

This species is closely related to *Elampus albipennis* (Mocsáry, 1889).

####### 35. Elampus spinipes

Taxon classificationAnimaliaHymenopteraChrysididae

(Mocsáry, 1890)

http://species-id.net/wiki/Ellampus_spinipes

Ellampus (Notozus) spinipes Mocsáry, 1890b: 49. Holotype ♀, China: “Mongolia meridionalis (Ta-wan), [China: Inner Mongolia] (49 (descr.), depository: ISEA-PAS)*.
Ellampus spinipes : Dalla Torre 1892: 18 (Mongolia [= Inner Mongolia], cat.).
Elampus spinipes : Kimsey and Bohart 1991: 171 (Mongolia [= Inner Mongolia], cat.).
Notozus spinipes : Bischoff 1913: 7 (Mongolia [= Inner Mongolia], cat.).
Omalus (Notozus) spinipes : Linsenmaier 1959: 16 (key), 24 (Mongolia [= Inner Mongolia], descr.).

######## Distribution.

China (Inner Mongolia).

######## Remarks.

The type locality in literature was always considered in Mongolia.

####### 36. Elampus yasumatsui

Taxon classificationAnimaliaHymenopteraChrysididae

(Tsuneki, 1948)

http://species-id.net/wiki/Notozus_yasumatsui

Notozus yasumatsui Tsuneki, 1948a: 116. Holotype ♀, China: Shanxi: Yüankii (116 (descr.) 117 (comp. notes), 128 (Shanxi, cat.), pl. 6 (figs. A–F), depository: KUM).
Omalus (Notozus) yasumatsui : Linsenmaier 1959: 16 (key), 24 (China, descr.).
Elampus yasumatsui : Kimsey and Bohart 1991: 173 (China: Shanxi, cat.); Kurzenko and Lelej 2007: 1003 (China: Shanxi, cat.).

######## Distribution.

China (Shanxi).

###### 7. Genus *Hedychridium* Abeille de Perrin, 1878

####### 37. Hedychridium ardens mongolicum

Taxon classificationAnimaliaHymenopteraChrysididae

Tsuneki, 1947

http://species-id.net/wiki/Hedychridium_ardens_mongolicum

Hedychridium ardens ssp. mongolicum Tsuneki, 1947: 47. Holotype ♀, China: Inner Mongolia: Apaka (47 (descr.), depository: NIAS).
Hedychridium ardens ssp. mongolicum : Linsenmaier 1959: 48 (possible synonym of *incensum* Mocsáry, 1914).
Hedychridium ardens : Kurzenko and Lelej 2007: 1003 (China: Gansu: Xiahe, cat.).

######## Distribution.

China (Inner Mongolia, Gansu).

######## Remarks.

Linsenmaier (1959) considered Hedychridium ardens ssp. mongolicum a possible junior synonym of *Hedychridium incensum* (Mocsáry, 1914). The type examination of Hedychridium ardens ssp. mongolicum is needed, because Hedychridium ardens ssp. mongolicum could be also related to Hedychridium ardens ssp. asianum Linsenmaier, 1997 described from Mongolia [= *integrum* ssp. *asianum*]. However, Linsenmaier did not check any types in this complicated species group.

####### 38. Hedychridium coriaceum

Taxon classificationAnimaliaHymenopteraChrysididae

(Dahlbom, 1854)

http://species-id.net/wiki/Hedychrum_coriaceum

Plate 6

Hedychrum coriaceum Dahlbom, 1854: 88. Lectotype ♀ design. by Morgan (1984: 10), Finland (depository: LZM)*.
Hedychridium coriaceum : Tsuneki 1947: 46 (China: Beijing, cat.); Linsenmaier 1959: 44 (key), 53 (tax., descr.), 200 (figs 189, 190); Kimsey and Bohart 1991: 184 (tax.), 191 (cat.).

######## Distribution.

China (Beijing). Europe (Linsenmaier 1959; Kimsey and Bohart 1991).

####### 39. Hedychridium cupreum asianum

Taxon classificationAnimaliaHymenopteraChrysididae

(Linsenmaier, 1997), n. comb.

http://species-id.net/wiki/Hedychridium_integrum_asianum

Hedychridium integrum ssp. asianum Linsenmaier, 1997a: 254. Holotype ♂, Mongolia: Central Aimag, Ulan Bator, 1900 m (254 (descr.), type series: China: Gansu, Xiahe, 3000–3500 m, depository: coll. Koschwitz, Germany).

######## Distribution.

China (Gansu). Mongolia (Linsenmaier 1997a).

######## Remarks.

Linsenmaier (1959) and Kimsey and Bohart (1991) listed *Hedychridium integrum* (Dahlbom, 1829) and *Hedychridium cupreum* (Dahlbom, 1845) as valid species, but *Hedychridium integrum* is a greenish form of *Hedychridium ardens* (Coquebert, 1801) (Paukkunen et al. 2014). Therefore we transfer the subspecies *Hedychridium asianum* Linsenmaier to *Hedychridium cupreum* (Dahlbom).

####### 40. Hedychridium cupreum

Taxon classificationAnimaliaHymenopteraChrysididae

(Dahlbom, 1845)

http://species-id.net/wiki/Hedychrum_cupreum

Plate 7

Hedychrum cupreum Dahlbom, 1845: 3. Lectotype ♀ design. by Paukkunen et al. (2014), Sweden [*nec* Switzerland] (3 (descr.), depository: NHMW)*.
Hedychridium integrum f. cupratum : Tsuneki 1948a: 123 (China: Shanxi: Chenhaissu, cat.), 128 (Shanxi, cat.).

######## Distribution.

China (Shanxi). Widely distributed from central and northern Europe to West Asia (Linsenmaier 1959, 1997a).

######## Remarks.

The species was listed as *cupratum* Dahlbom by Tsuneki (1948a), a species endemic to the European Alps and is to be excluded from the Chinese fauna. It is likely that Tsuneki was referring to *cupreum* (Dahlbom, 1854), which is widely distributed from central and northern Europe all the way to West Asia.

####### 41. Hedychridium flos

Taxon classificationAnimaliaHymenopteraChrysididae

(Semenov-Tian-Shanskij, 1954)

http://species-id.net/wiki/Cyrteuchrum_flos

Plate 8

Cyrteuchrum flos Semenov-Tian-Shanskij, 1954: 105. Holotype ♀, Kazakhstan: Imam-Baba (depository: ZIN)*.
Cyrteuchrum nivifrons Semenov-Tian-Shanskij, 1967: 134. Holotype ♂, China [Xinjiang]: Bugas near Hami [Kumul] (depository: ZIN)* (synonymised by Kimsey and Bohart 1991: 194).
Hedychridium flos : Kimsey and Bohart 1991: 194 (cat.); Rosa et al. 2013: 5 (China: Xinjiang [= Heilongjiang], cat.).

######## Distribution.

China (Xinjiang). Iran, Kazakhstan (Semenov-Tian-Shanskij 1967; Rosa et al. 2013).

####### 42. Hedychridium roborovskii

Taxon classificationAnimaliaHymenopteraChrysididae

Semenov-Tian-Shanskij, 1967

http://species-id.net/wiki/Hedychridium_roborovskii

Plate 9

Hedychridium roborovskii Semenov-Tian-Shanskij, 1967: 129. Holotype ♀, China: Xinjiang: Gashun Gobi, Sachzou oasis (129 (descr.), depository: ZIN)*.
Hedychridium roborovskii : Kimsey and Bohart 1991: 203 (China: Gashun Gobi, cat.).

######## Distribution.

China (Xinjiang).

####### 43. Hedychridium roseum

Taxon classificationAnimaliaHymenopteraChrysididae

(Rossi, 1790)

http://species-id.net/wiki/Chrysis_carnea_rosea

Chrysis carnea var. rosea Rossi, 1790: 75. Syntypes, Italy: Tuscany (75 (descr.), depository: MNHU?).
Hedychridium roseum : Tsuneki 1953a: 55 (Manchuria: Fen-Tien [= Liaoning], cat., distr.); Tsuneki 1953b: 23 (Manchuria, cat.); Linsenmaier 1959: 57 (*roseum* group), 58 (Manchuria, key, tax., distr.), 198 (figs 105, 106, 115), 199 (fig. 137); Banaszak 1980: 14 (Manchuria, tax., biol.); Kimsey and Bohart 1991: 180 (fig. 62m), 185 (tax.), 203 (cat.).
Hedychridium roseum roseum : Arens 2010: 406 (Manchuria, cat.), 410 (tax., descr.), 442 (figs 9e, 9f).

######## Material examined.

1 ex., Heilongjiang: Harbin, 20.VII.1953 leg. Alin (NMLS).

######## Distribution.

China (Heilongjiang, Liaoning). Widely distributed in the Palaearctic Region (Tsuneki 1953a, 1953b; Linsenmaier 1959, 1999; Kurzenko and Lelej 2007).

###### 8. Genus *Hedychrum* Latreille, 1802

####### 44. Hedychrum chalybaeum

Taxon classificationAnimaliaHymenopteraChrysididae

Dahlbom, 1854

http://species-id.net/wiki/Hedychrum_chalybaeum

Hedychrum chalybaeum Dahlbom, 1854: 64. Syntypes ♂♂, Europe: ‘*Europa media et meridionali*’, Russia, Prussia, Silesia (64 (descr.), depositories: MNHU, LZM)*.
Hedychrum coerulescens Shuckard, 1837: Tsuneki 1947: 51 (China: Inner Mongolia: Apaka, cat.) [misid.].
Hedychrum szaboi Mocsáry, 1889: 167. Lectotype ♀ (design. by Móczár (1964a: 440), Germany: Thuringia (167 (descr.), depository: HNHM)* (synonymised by Trautmann 1927).
Hedychrum chalybaeum : Tsuneki 1953a: 55 (China: Heilongjiang: Harbin, cat., distr.); Linsenmaier 1959: 36 (key), 39 (Manchuria, tax., descr.), 197 (figs 65–69); Móczár 1967: 39 (China, key, tax., descr.); Banaszak 1980: 16 (tax., distr.), 17 (China, biol.); Kimsey and Bohart 1991: 210 (tax.), 212 (cat.).
Hedychrum komarovi Semenov-Tian-Shanskij, 1967: 138. Holotype ♂, China: Gansu (138 (descr.), depository: ZIN)* (synonymised by Kimsey and Bohart 1991).
Hedychrum martynovi Semenov-Tian-Shanskij, 1967: 138. Holotype ♂, Manchuria: Langashi (138 (descr.), depository: ZIN)* (synonymised by Kimsey and Bohart 1991).

######## Distribution.

China (Heilongjiang, Inner Mongolia, Gansu). Widely distributed in the Palaearctic Region (Linsenmaier 1959; Kurzenko and Lelej 2007).

######## Remarks.

*Hedychrum szaboi* Mocsáry, 1889 is the female of *Hedychrum chalybaeum* Dahlbom (Trautmann 1927).

####### 45. Hedychrum davidi

Taxon classificationAnimaliaHymenopteraChrysididae

du Buysson, 1900

http://species-id.net/wiki/Hedychrum_davidi

Hedychrum davidi du Buysson, 1900: 131. Holotype ♀, China: Beijing (131 (descr.), depository: MNHN).
Hedychrum davidi : Bischoff 1913: 18 (China, cat.); Kimsey and Bohart 1991: 213 (China: Beijing, cat.); Kurzenko and Lelej 2007: 1003 (China: Beijing, cat.).

######## Distribution.

China (Beijing).

####### 46. Hedychrum formosanum

Taxon classificationAnimaliaHymenopteraChrysididae

Mocsáry, 1911

http://species-id.net/wiki/Hedychrum_formosanum

Plate 10

Hedychrum formosanum Mocsáry, 1911: 458. Holotype ♂, Taiwan: Takao [= Kaohsiung] (458 (descr.), depository: HNHM)*.
Hedychrum formosanum : Mocsáry 1913b: 613 (Taiwan: Takao [= Kaohsiung], cat.), 619 (cat.); Bischoff 1913: 19 (Taiwan, cat.); Uchida 1927: 150 (Taiwan, cat.); Uchida 1933: 2 (Taiwan, cat.); Tsuneki 1970b: 4 (Taiwan: Shihtsulu, Kuonfu, tax., descr.), 5 (comp. notes); Tsuneki 1970c: 48 (Formosa, tax.).

######## Distribution.

China (Taiwan).

######## Remarks.

Tsuneki (1970b) gave a detailed comparison with the similar Japanese species *Hedychrum okai* Tsuneki.

####### 47. Hedychrum gerstaeckeri

Taxon classificationAnimaliaHymenopteraChrysididae

Chevrier, 1869

http://species-id.net/wiki/Hedychrum_gerstaeckeri

Plate 11

Hedychrum gerstaeckeri Chevrier, 1869: 47. Syntypes ♀♀, Switzerland: Nyon, Beau-lac (47 (descr.), depository: MHNG)*.
Hedychrum marianum Mocsáry, 1911: 450. Lectotype ♀ design. by French (in Bohart and French 1986: 341), China (depository: HNHM)*.
Hedychrum marianum : Bischoff 1913: 19 (China, cat.); Wu 1941: 118 (China, cat.); Linsenmaier 1959: 41 (syn. of *japonicum* Mocsáry); Bohart and French 1986: 341 (China, lectotype design.); Kimsey and Bohart 1991: 214 (China, syn.).
Hedychrum gerstaeckeri f. marianum : Tsuneki 1947: 50 (tax., possible syn. of *japonicum* Cameron, 1887).
Hedychrum gerstaeckeri : Tsuneki 1947: 50 (comp. notes); Linsenmaier 1959: 36 (key), 37 (key), 40 (tax., descr.), 198 (figs 81–83); Kimsey and Bohart 1991: 214 (Taiwan, cat.); Linsenmaier 1997b: 33 (key), 62 (Taiwan, tax., descr., fig. 33), 63 (colour picture).
?Hedychrum gerstaeckeri ssp. formosaiense Linsenmaier, 1959: 41. Holotype ♂, Taiwan (41 (descr.), depository: RMNH).

######## Distribution.

China (Taiwan and mainland) (Linsenmaier 1959). Widely distributed in the Palaearctic Region (Mocsáry 1911; Trautmann 1927; Linsenmaier 1959; Kurzenko and Lelej 2007).

######## Remarks.

The placement of *Hedychrum gerstaeckeri formosaiense* Linsenmaier is uncertain. According to Tsuneki (1970b), it could be synonym or a form of *Hedychrum japonicum* Cameron, 1887.

####### 48. Hedychrum gracile

Taxon classificationAnimaliaHymenopteraChrysididae

Semenov-Tian-Shanskij, 1967

http://species-id.net/wiki/Hedychrum_gracile

Plate 12

Hedychrum gracile Semenov-Tian-Shanskij, 1967: 139. Holotype ♀, China: Gansu (139 (descr.), depository: ZIN)*.
Hedychrum gracile : Kimsey and Bohart 1991: 214 (China: Han Shui, cat.).

######## Distribution.

China (Sichuan).

######## Remarks.

The label of the holotype is handwritten by Semenov: Sichuan, Maozhou-Matajgi, leg. Potanin, 27.VIII.1893. Actually, the date (27.VIII.1893) and the collector (Potanin) given in the description are the same as those on the type label. Semenov-Tian-Shanskij mistakenly placed the type locality in Gansu Province instead of the adjacent Sichuan Province.

####### 49. Hedychrum japonicum

Taxon classificationAnimaliaHymenopteraChrysididae

Cameron, 1887

http://species-id.net/wiki/Hedychrum_japonicum

Hedychrum japonicum Cameron, 1887: 123. Holotype ♂, Japan: Fukui (123 (descr.), depository: BMNH).
Hedychrum japonicum : Tsuneki 1946: 35 (North China, tax.), 39 (descr.); Tsuneki 1947: 50 (comp. notes); Tsuneki 1953b: 23 (North China, tax., aberr.); Kimsey and Bohart 1991: 214 (cat.).
Hedychrum gerstaeckeri ssp. japonicum : Linsenmaier 1959: 41 (North China, descr.); Tsuneki 1970b: 33 (North China, Taiwan, tax.); Tsuneki 1970b: 3 (tax.), 4 (Taiwan: Wushue, notes); Tsuneki 1970c: 47 (Taiwan, North China, key, tax.).

######## Distribution.

North China and Taiwan. Korea, Japan (Tsuneki 1953b; Linsenmaier 1959).

######## Host.

*Hedychrum gerstaeckeri japonicum* was observed flying around the nests of *Cerceris* spp., in particular those of *Cerceris hortivaga* Kohl (Hymenoptera, Crabronidae). For other host relationships observed in Europe see Rosa (2006).

######## Remarks.

Tsuneki (1946) considered *Hedychrum marianum* as the junior synonym of *Hedychrum japonicum*.

####### 50. Hedychrum latitudum

Taxon classificationAnimaliaHymenopteraChrysididae

Linsenmaier, 1959

http://species-id.net/wiki/Hedychrum_latitudum

Hedychrum latitudum Linsenmaier, 1959: 39. Holotype ♂, China: Heilongjiang: Harbin (36 (key), 39 (descr.), 197 (figs 75, 76), depository: NMLS)*.
Hedychrum latitudum : Kimsey and Bohart 1991: 215 (China: Manchuria, cat.); Kurzenko and Lelej 2007: 1003 (China: Heilongjiang, cat.).

######## Material examined.

Heilongjiang: 1♂, Harbin, 20.VII.1953, leg. Alin / Type (NMLS).

######## Distribution.

China (Heilongjiang).

####### 51. Hedychrum longicolle

Taxon classificationAnimaliaHymenopteraChrysididae

Abeille, 1877

http://species-id.net/wiki/Hedychrum_longicolle

Plate 13

Hedychrum longicolle Abeille, 1877: 65. Lectotype ♀ design. by Kimsey (1986: 108), France: Marseille, Toulon (65 (descr.), depositories: MNHN, MHNG)*.
Hedychrum longicolle : du Buysson 1904: 257 (China, cat.); Trautmann 1927: 71 (China, tax., descr., distr.); Berland and Bernard 1938: 53 (key), 55 (China, tax., descr., distr.), 56 (figs 68–69); Balthasar 1954: 120 (tax.), 122 (China, key, tax., descr., distr.); Linsenmaier 1959: 36 (key), 37 (key), 41 (China, tax., descr., distr.), 198 (figs 84–86); Móczár 1967: 41 (China, key, tax., descr., distr.); Banaszak 1980: 19 (China, tax., distr.); Kimsey 1986: 108 (lectotype des.); Kimsey and Bohart 1991: 209 (figs 64d, 64h, 64l), 210 (tax.), 215 (cat.); Kurzenko and Lelej 2007: 1003 (China, cat.).

######## Distribution.

China. Widely distributed in the Palaearctic Region (Linsenmaier 1959, 1999; Kurzenko and Lelej 2007).

####### 52. Hedychrum manchurianum

Taxon classificationAnimaliaHymenopteraChrysididae

Tsuneki, 1950

http://species-id.net/wiki/Hedychrum_manchurianum

Hedychrum manchurianum Tsuneki, 1950: 64. Holotype ♀, Manchuria (64 (Kaiyüan, descr.), depository: EIHU).
Hedychrum manchurianum : Tsuneki 1953a: 57 (Manchuria, tax., fig. 1); Kimsey and Bohart 1991: 216 (Manchuria, cat.); Kurzenko and Lelej 2007: 1003 (Northeast China, cat.).

######## Distribution.

China (Liaoning).

####### 53. Hedychrum niemelai

Taxon classificationAnimaliaHymenopteraChrysididae

Linsenmaier, 1959

http://species-id.net/wiki/Hedychrum_aureicolle_niemelai

Plate 14

Hedychrum aureicolle ssp. niemelai Linsenmaier, 1959: 38. Holotype ♀, Switzerland: Wallis (63 (type series: Manchuria, descr.), 197 (figs 62–64), depository: NMLS)*.
Hedychrum aureicolle ssp. niemelai : Banaszak 1980: 19 (Manchuria, tax., distr., biol.); Linsenmaier 1997b: 33 (key), 62 (Manchuria, tax., descr., distr., fig. 32).
Hedychrum niemelai : Kimsey and Bohart 1991: 217 (cat.).

######## Material examined.

Heilongjiang: Paratype, 1♀, Harbin, 20 July (NMLS).

######## Distribution.

China (Heilongjiang). Switzerland (Linsenmaier 1959).

####### 54. Hedychrum nobile

Taxon classificationAnimaliaHymenopteraChrysididae

(Scopoli, 1763)

http://species-id.net/wiki/Sphex_nobile

Sphex nobile Scopoli, 1763: 297. Holotype ♀; Italy (297 (descr.), type lost).
Hedychrum nobile : Hammer 1936: 3 (China: Kansu [= Gansu], cat.); Tsuneki 1947: 47 (China: Inner Mongolia: Apaka, cat., distr.); Tsuneki 1953a: 56 (Manchuria: Furaruki, North of Tsitsihar [= Qiqihar], cat., distr.); Tsuneki 1953b: 23 (North China, Manchuria, cat.); Kimsey and Bohart 1991: 209 (fig. 209e), 210 (tax.), 217 (cat.).

######## Distribution.

China (Heilongjiang, Inner Mongolia, Gansu, Tianjin). Widely distributed in the Palaearctic Region from Europe and North Africa to Turkmenistan, Siberia, Korea, Japan (Tsuneki 1953b; Linsenmaier 1959; Kimsey and Bohart 1991; Kurzenko and Lelej 2007).

####### 55. Hedychrum simile

Taxon classificationAnimaliaHymenopteraChrysididae

Mocsáry, 1889

http://species-id.net/wiki/Hedychrum_simile

Plate 15

Hedychrum cyaneum Mocsáry (in Radoszkowski), 1889: 10 *nec* Brullé, 1846. Lectotype ♀ design. by French (in Bohart and French 1986: 341), China “Ta-schian-sy” (HNHM)*.
Hedychrum simile Mocsáry, 1889: 157. Replacement name for *Hedychrum cyaneum* Radoszkowski, 1889 *nec* Brullé, 1846.
Hedychrum simile : Dalla Torre, 1892: 35 (China, cat.); Mocsáry 1890a: 61 (China borealis, cat.); Tsuneki 1946: 37 (Manchuria, tax.); Tsuneki 1947: 51 (China: Inner Mongolia: Apaka; North China: Tientsing [= Tianjin], tax., distr.); Tsuneki 1953b: 23 (North China, Manchuria, tax.); Linsenmaier 1959: 36 (key), 37 (key), 39 (Manchuria, tax., descr.), 198 (figs 79, 80); Bohart and French 1986: 341 (lectotype designation by French, China (“borealis”): Ta-schian-sy); Kimsey and Bohart 1991: 220 (China, Manchuria, cat.); Kurzenko and Lelej 2007: 1003 (Northeast China, tax.); Terayama et al. 2010: 6 (figs.), 10 (China, cat.), 12 (tab., biol.).
Hedychrum simili (!): Uchida 1927: 151 (North China, cat.).
Hedychrum simile f. pullatum Tsuneki, 1953b: 23. Syntype ♂♀, Korea, Shôyôzan (23 (descr.), depository: NIAS).
Hedychrum simile ssp. aereum Tsuneki, 1970a: 34. Holotype ♀, Japan, Chiba (34 (descr.), depository: NIAS).

######## Distribution.

Northeast China, Inner Mongolia, Tianjin. Widely distributed in the Palaearctic Region from Europe and North Africa to Central Asia, Mongolia, Korea, Japan and Russian Far East (Tsuneki 1953b; Linsenmaier 1959; Kimsey and Bohart 1991; Kurzenko and Lelej 2007).

######## Host.

*Cerceris arenaria* (Linnaeus, 1758) (Hymenoptera, Crabronidae) (Tsuneki 1979).

######## Remarks.

Mocsáry examined at least two specimens of both sexes (see the simbols male and female in the first couplet). However, in the original description Mocsáry (in Radoszkowski) gave the description of *Hedychrum simile* (sub *cyaneum* Mocsáry nec Brullé) based only on the male housed in the Radoszkowski collection and dissected by the Russian entomologist, who drew the genitalia. The type locality given by Radoszkowski is “Siberia orientalis”, and it has to be referred only to the male housed in the Radoszkowski collection. In the MNHM, we examined the rest of the type series listed by Mocsáry (1889): a female specimen collected in China (Ta-schian-sy). French (1986) designated this female as lectotype. However, this female belongs to a different species compared with the male collected in Siberia. The female lectotype is characterised by the very long pronotum (similar to *Hedychrum longicolle*) and sharp and pointing out propodeal angles, while the male has a short pronotum and wide and triangular propodeal angles. A revision of the blue Asian species of *Hedychrum* is missing and urgently needed.

####### 56. Hedychrum sinicum

Taxon classificationAnimaliaHymenopteraChrysididae

Semenov-Tian-Shanskij, 1967

http://species-id.net/wiki/Hedychrum_sinicum

Plate 16

Hedychrum sinicum Semenov-Tian-Shanskij, 1967: 140. Holotype ♂, China: Sichuan (depository: ZIN)*.
Hedychrum sinicum : Kimsey and Bohart 1991: 220 (China: Sechuan [= Sichuan], cat.).

######## Distribution.

China (Sichuan).

####### 57. Hedychrum taiwanense

Taxon classificationAnimaliaHymenopteraChrysididae

Tsuneki, 1970

http://species-id.net/wiki/Hedychrum_taiwanense

Hedychrum taiwanense Tsuneki, 1970b: 5. Holotype ♂, Taiwan: Liyuchih [= Li-yu Ch’ih]: Hualien (5 (tax. descr.), depository: NIAS).
Hedychrum taiwanense : Kimsey and Bohart 1991: 221 (Taiwan: Liyuchih Prov., Hualien, cat.).

######## Distribution.

China (Taiwan).

####### 58. Hedychrum takasago

Taxon classificationAnimaliaHymenopteraChrysididae

Tsuneki, 1970

http://species-id.net/wiki/Hedychrum_takasago

Hedychrum takasago Tsuneki, 1970b: 4. Holotype ♀, Taiwan: Chuchi: Chiai (4 (tax., descr.), 5 (type series: Taiwan: Chiai Prov: Chuchi; Hualien Prov: Kuanfu, Pingtung Prov.: Henchung, tax., descr. comp. notes), depository: OMNH, not NIAS).
Hedychrum takasago : Kimsey and Bohart 1991: 221 (Taiwan: Chuchi, cat.).

######## Distribution.

China (Taiwan).

###### 9. Genus *Holophris* Mocsáry, 1890

####### 59. Holophris taiwanus

Taxon classificationAnimaliaHymenopteraChrysididae

(Tsuneki, 1970)

http://species-id.net/wiki/Omalus_taiwanus

Omalus taiwanus Tsuneki, 1970b: 2. Holotype ♂, Taiwan: Nantou: Chienching (2 (tax., descr., figs 1–4), 3 (type series: Taiwan: Nantou Province: Chienching, Puli), depository: OMNH, not NIAS).
Holophris taiwanus : Kimsey and Bohart 1991: 225 (Taiwan, cat.).

######## Distribution.

China (Taiwan).

###### 10. Genus *Holopyga* Dahlbom, 1845

####### 60. Holopyga amoenula virideaurata

Taxon classificationAnimaliaHymenopteraChrysididae

Linsenmaier, 1951

http://species-id.net/wiki/Holopyga_amoenula_virideaurata

Holopyga amoenula var. virideaurata Linsenmaier, 1951: 16. Holotype ♀, Greece: Rhodes (16 (descr.), depository: NMLS)*.
Holopyga amoenula ssp. virideaurata : Linsenmaier 1959: 31 (? China, tax.).
Holopyga amoenula : Kimsey and Bohart 1991: 228 (cat.).

######## Material examined.

Heilongjiang: 1♂, Harbin, 6.VII.1947; 1♀, id., 10.VII.1949; 1♂, id., 20.VII.1953, all specimens leg. Alin; 1♀, China; 1♂, Kouy-Théou Cavalarie 1921 (NMLS).

######## Distribution.

China (Heilongjiang, Guizhou). Widely distributed in the Palaearctic Region (Kimsey and Bohart 1991).

######## Remarks.

Linsenmaier (1959) dubiously considered the specimens from Harbin to be Holopyga amoenula ssp. virideaurata. These specimens were still identified as *virideaurata* in his collection and seem to fit the current interpretation of the taxon. At present, *Holopyga amoenula amoenula* is an endemism of the Mediterranean island of Rhodes, but some specimens may also be found in southern Greece. A re-evaluation of all the subspecies and taxa related to *Holopyga amoenula* s. str. is urgently needed.

####### 61. Holopyga chrysonota

Taxon classificationAnimaliaHymenopteraChrysididae

(Förster, 1853)

http://species-id.net/wiki/Ellampus_chrysonotus

Ellampus chrysonotus Förster, 1853: 347. Holotype ♀, Hungary: Budapest (347 (descr.), depository: MNHU)*.
Holopyga gloriosa f. chrysonota : Tsuneki 1947: 45 (cat.), 46 (China: Beijing, Inner Mongolia, tax., distr.).
Holopyga chrysonota : Linsenmaier 1959: 30 (key), 32 (tax., descr.), 197 (fig. 34); Kimsey and Bohart 1991: 227 (fig. 68a), 230 (cat.); Kurzenko and Lelej 2007: 1004 (China, cat.).

######## Distribution.

China (Beijing, Inner Mongolia). Widely distributed in the Palaearctic Region (Kurzenko and Lelej 2007).

######## Remarks.

The identification of this taxon is dubious because Tsuneki (1947) did not separate *ignicollis* Dahlbom, 1854 from *chrysonota* (Förster, 1853). The two were later recognized as valid species by Linsenmaier (1959), who provided both a key and characteristics to distinguish them from one another. The specific name *Chrysis gloriosa* Fabricius (=*Holopyga gloriosa*) was suppressed by ICZN (1998).

####### 62. Holopyga generosa

Taxon classificationAnimaliaHymenopteraChrysididae

(Förster, 1853)

http://species-id.net/wiki/Ellampus_generosus

Ellampus generosus Förster, 1853: 349. Syntypes ♂♂, Germany: Aachen (349 (descr.), depository: MNHU)*.
Holopyga ovata Dahlbom, 1854: 51. Syntypes, Italy, Austria, Prussia, Sweden (51 (descr.), depositories: MRSN, MNHU, LZM)*. Linsenmaier 1959: 29 (key), 31 (China, tax., descr.) (synonymised by Linsenmaier 1997a).
Holopyga fastuosa generosa : Linsenmaier 1997a: 250 (China, tax.); Linsenmaier 1997b: 32 (figs 10, b4, c5), 33 (key), 57 (China, tax., descr., distr.).
Holopyga generosa : Rosa 2006: 39 (ecol.), 41 (ecol.), 42 (ecol.), 52 (ecol.), 55 (distr.), 78 (cat.), 137 (key), 146 (tax., descr., distr.).

######## Distribution.

China. Widely distributed in the Palaearctic Region (Linsenmaier 1959).

######## Remarks.

Kimsey and Bohart (1991) considered *Holopyga generosa* a junior synonym of *Holopyga chrysonota*, while *fastuosa* and *ovata* synonyms of *amoenula.* Linsenmaier (1997a) considered *generosa* (Förster) as a subspecies of *Holopyga fastuosa* (Lucas).

####### 63. Holopyga ignicollis

Taxon classificationAnimaliaHymenopteraChrysididae

Dahlbom, 1854

http://species-id.net/wiki/Holopyga_ovata_ignicollis

Holopyga ovata var. ignicollis Dahlbom, 1854: 53 (given as var. h). Syntypes ♂♀, Greece, Rhodes, Austria (53 (descr.), depositories: NHMW, MNHU)*.
Holopyga gloriosa var. aureomaculata Abeille de Perrin, 1879: 32. Syntypes ♂♂, France: Marseille (32 (descr.), depository: MNHN)*.
Holopyga gloriosa var. aureomaculata : du Buysson 1900: 128 (China: Beijing, cat.).

######## Distribution.

China (Beijing). Central and southern Europe, North Africa, Turkey (Linsenmaier 1959).

######## Remarks.

*Holopyga aureomaculata* Abeille is recognized to be the male of *Holopyga ignicollis* Dahlbom. This is a dubious identification, which can perhaps be referenced to *Holopyga chrysonota*. In Kimsey and Bohart (1991: 230) *Holopyga ignicollis* is synonym of *Holopyga chrysonota*, but the two species are clearly distinct.

###### 11. Genus *Omalus* Panzer, 1806

####### 64. Omalus aeneus

Taxon classificationAnimaliaHymenopteraChrysididae

(Fabricius, 1787)

http://species-id.net/wiki/Chrysis_aenea

Plates 17 , 18

Chrysis aenea Fabricius, 1787: 284. Holotype ♀, Germany: Halle “*Halae Saxonum*” (284 (descr.), depository: ZMU)*.
Omalus aeneus var. pygialis du Buysson, 1887: 170. Syntypes ♂, ♀; China, Caucasus (170 (descr.), depository: MNHN).
Ellampus aeneus var. pygialis : Mocsáry 1889: 98 (China, descr.); Dalla Torre 1892: 8 (China, cat.); Bischoff 1913: 8 (China, cat.).
Ellampus sauteri Mocsáry, 1913b: 613. Holotype ♀, Taiwan, Taihorinsho (613 (descr.), 619 (Taiwan, cat.), depository: HNHM)*.
Ellampus sauteri : Uchida 1927: 150 (Taiwan, cat.); Uchida 1933: 1 (Taiwan, cat.).
Philoctetes sauteri : Tsuneki 1946: 35 (Manchuria, Taiwan).
Omalus (Omalus) sauteri : Linsenmaier 1959: 15 (key), 19 (Taiwan, tax., descr., *punctulatus* group).
Omalus aeneus sauteri : Tsuneki 1970b: 1 (Taiwan: Nantou Prov.: Chienching Wushe (1800m), tax., descr.).
Omalus aeneus : Kimsey and Bohart 1991: 245 (Taiwan, cat., fig. 76g); Wei et al. 2014b: 31 (key), 32 (China: Inner Mongolia, Helanshan: Gulaben, Dayanggou, Halawuchagou, Shuimogou, Qianggangling, Halawuchagou; Taiwan, descr., tax.), 33 (pl. 1), 34 (pl. 2A–2F), 35 (pl. 3), 36 (pl. 4A–4F).

######## Distribution.

China (Inner Mongolia, Taiwan). In literature the records for mainland China are listed under the name *pygialis* du Buysson and the Taiwanese citations are listed under the name *sauteri*. It is widely distributed in the Palearctic from Europe to the Russian Far East and in the Nearctic, where it is very likely to have been introduced via commerce (Kimsey and Bohart 1991; Kurzenko and Lelej 2007).

######## Remarks.

Du Buysson described *pygialis* based on two syntypes collected in China and Caucasus: “*Deux esemplaires* ♂♀: *Chine, Caucase*” [not *Chinese Caucasus* as listed in Kimsey and Bohart, 1991]. Tsuneki (1946) considered *Ellampus potanini* as synonym of *sauteri*.

####### 65. Omalus berezovskii

Taxon classificationAnimaliaHymenopteraChrysididae

(Semenov-Tian-Shanskij, 1932)

http://species-id.net/wiki/Ellampus_berezovskii

Plate 19

Ellampus (Dictenulus) berezovskii Semenov-Tian-Shanskij, 1932: 12. Holotype ♀, China: Setshuan [= Sichuan]: Cho-dzi-gou, Lun-ngan-fu (12 (descr.), depository: ZIN)*.
Omalus berezovskii : Kimsey and Bohart 1991: 247 (China: Setshuan [= Sichuan], cat.); Wei et al. 2014b: 31 (key), 35 (China: Ningxia, Liupanshan Forest Park, tax.), 36 (pl. 4, descr.), 37 (pl. 5), 38 (pl. 6A–6F).

######## Distribution.

China (Ningxia, Sichuan).

####### 66. Omalus helanshanus

Taxon classificationAnimaliaHymenopteraChrysididae

Wei, Rosa, Liu & Xu, 2014

http://species-id.net/wiki/Omalus_helanshanus

Omalus helanshanus Wei, Rosa, Liu & Xu, 2014: 39. Holotype ♀, China: Inner Mongolia, Helanshan, Gulaben (32 (key), 39 (type series: Inner Mongolia, Helanshan, Gulaben, Dayanggou; Shuimogou; Habeigou, Huangliangzi; Halawuchagou; Halawubeigou; Halawu, descr.), 40 (pl. 7), 41 (pl. 8A–8F), depository: SCAU)*.

######## Distribution.

China (Inner Mongolia).

####### 67. Omalus imbecillus

Taxon classificationAnimaliaHymenopteraChrysididae

(Mocsáry, 1889)

http://species-id.net/wiki/Ellampus_imbecillus

Plate 20

Ellampus imbecillus Mocsáry, 1889: 98. Lectotype ♀ design. by French (in Bohart and French 1986: 341), Turkmenistan: Pendgikent (depository: HNHM)*.
Holophris imbecillus : Kimsey and Bohart 1991: 225.
Omalus imbecillus : Rosa 2005: 12; Wei et al. 2014b: 31 (key), 41 (tax.), 42 (China: Yunnan, Kaiyuannan River; Gaoligonshan National Nature Reserve, descr., pl. 9), 43 (pl. 10A–10F).

######## Distribution.

China (Yunnan). Laos, Russia, Turkey, Iran (Kimsey and Bohart 1991; Rosa et al. 2013).

####### 68. Omalus potanini

Taxon classificationAnimaliaHymenopteraChrysididae

(Semenov-Tian-Shanskij, 1932)

http://species-id.net/wiki/Ellampus_potanini

Plate 21

Ellampus (Dictenulus) potanini Semenov-Tian-Shanskij, 1932: 11. Lectotype ♂ design. by Kimsey (1986: 107), China: Sichuan: river Sjao-tzhin-cho (11 (type series: China: Sichuan: river Sjao-tzhin-cho; river Fu-bjan cho, Chun-shujgu, Li-fan, descr.), depository: ZIN)*.
Philoctetes (Holophris) potanini : Tsuneki 1953a: 55 (West China: Setshuan and Manchuria, tax., distr.).
Ellampus potanini : Kimsey 1986: 107 (China: Setchuan [= Sichuan]: Sjao-tzhin-cho, lectotype design.).
Omalus potanini : Kimsey and Bohart 1991: 249 (China: Setshuan [= Sichuan], cat.); Wei et al. 2014b: 32 (key), 44 (China: Liaoning, Sichuan, tax., descr.).

######## Distribution.

China (Liaoning, Sichuan).

######## Remarks.

Only two specimens erroneously labelled as paratypes and collected in the same place (Sichuan: river Sjao-tzhin-cho, leg. Potanin) are present in the Semenov collection. We don’t know whether the lectotype label or the lectotype is lost.

####### 69. Omalus probiaccinctus

Taxon classificationAnimaliaHymenopteraChrysididae

Wei, Rosa, Liu & Xu, 2014

http://species-id.net/wiki/Omalus_probiaccinctus

Omalus probiaccinctus Wei, Rosa, Liu & Xu, 2014: 44. Holotype ♀, China: Guizhou, Suiyang, Kuankuoshui National Reserve (31 (key), 44 (tax.), 45 (descr., pl. 11), 46 (pl. 12A–12F), depository: SCAU)*.

######## Distribution.

China (Guizhou).

####### 70. Omalus pseudoimbecillus

Taxon classificationAnimaliaHymenopteraChrysididae

Wei, Rosa, Liu & Xu, 2014

http://species-id.net/wiki/Omalus_pseudoimbecillus

Omalus pseudoimbecillus Wei, Rosa, Liu & Xu, 2014: 44. Holotype ♀, China: Yunnan, Yimen, Longquan Park (31 (key), 47 (type series: Yunnan, Yimen, Longquan Park; Yunlong, Tianchi National Nature Reserve; Jingdong, Jingping, tax., pl. 13), 48 (pl. 14A–14F), 49 (descr.), depository: SCAU)*.

######## Distribution.

China (Yunnan).

####### 71. Omalus tibetanus

Taxon classificationAnimaliaHymenopteraChrysididae

Wei, Rosa, Liu & Xu, 2014

http://species-id.net/wiki/Omalus_tibetanus

Omalus tibetanus Wei, Rosa, Liu & Xu, 2014: 49. Holotype ♀, China: Tibet, Chayu, Cibagou (31 (key), 49 (tax.), 50 (descr., pl. 15), 51 (pl. 16A–16F), 49 (descr.), depository: SCAU)*.

######## Distribution.

China (Tibet).

###### 12. Genus *Philoctetes* Abeille de Perrin, 1878

####### 72. Philoctetes deauratus

Taxon classificationAnimaliaHymenopteraChrysididae

(Mocsáry, 1914), n. comb.

http://species-id.net/wiki/Ellampus_deauratus

Ellampus deauratus Mocsáry, 1914: 2. Holotype ♀, China: Tientsin [= Tianjin] (2 (descr.), depository: BMNH).
Ellampus deauratus : Tsuneki, 1947: 45 (North China: Tieng-tsing [= Tianjin], comp. notes); Tsuneki, 1948a: 119 (comp. notes).
Omalus (Omalus) deauratus : Linsenmaier 1959: 20 (China, tax., *pusillus* group).
Pseudomalus deauratus : Kimsey and Bohart 1991: 267 (China: Tientsin, cat.).

######## Distribution.

China (Tianjin) (Mocsáry 1914).

######## Remarks.

After the examination of other specimens labeled as types of *Ellampus deauratus* by Mocsáry in the HNHM, we have included this taxon in the genus *Philoctetes* Abeille, based on the characteristics given by Kimsey and Bohart (1991).

####### 73. Philoctetes duplipunctatus

Taxon classificationAnimaliaHymenopteraChrysididae

(Tsuneki, 1948)

http://species-id.net/wiki/Chrysellampus_duplipunctatus

Chrysellampus near *harmandi*: Tsuneki 1946: 33 (China: Shanxi, tax.).
Chrysellampus duplipunctatus Tsuneki, 1948a: 120. Holotype ♀, China: Shanxi, Wutai Shan (120 (descr.), 122 (Wutaishan, comp. notes), 128 (Shanxi, cat.), pl. 7 (figs A, B), pl. 8 (figs A–E), depository: KUM).
Chrysellampus duplipunctatus f. suzukii Tsuneki, 1948a: 122. Holotype ♀, China: Shanxi, Yangchêng (122 (descr.), 128 (cat.), depository: KUM).
Chrysellampus duplipunctatus f. variegatus Tsuneki, 1950: 63. Syntypes ♂♀, Korea, Manchuria: Kay-juan (63 (descr.), depository: NIAS).
Chrysellampus duplipunctatus : Tsuneki 1953a: 55 (Manchuria: Kaiyüan, tax.); Tsuneki 1953b: 23 (North China, Manchuria, tax.).
Chrysellampus duplipunctatus f. variegatus : Tsuneki 1953a: 55 (Manchuria: Kaiyüan, distr., cat.); Tsuneki 1953b: 23 (tax.).
Omalus (Chrysellampus) duplipunctatus : Linsenmaier 1959: 22 (China, tax., descr.).
Philoctetes duplipunctatus : Kimsey and Bohart 1991: 255 (China, cat.); Kurzenko and Lelej 2007: 1004 (China, cat.).

######## Distribution.

China (Liaoning, Jilin, Shanxi). Korea and Russian Far East (Tsuneki 1953b; Kurzenko and Lelej 2007; Lelej and Kurzenko 2012).

######## Remarks.

Kimsey and Bohart (1991) considered the forms Philoctetes duplipunctatus f. suzukii and Philoctetes duplipunctatus f. variegatus invalid names. However, according to the ICZN, the two names are indeed valid.

####### 74. Philoctetes heros

Taxon classificationAnimaliaHymenopteraChrysididae

(Semenow, 1892)

http://species-id.net/wiki/Elampus_heros

Plate 22

Elampus heros Semenow, 1892c: 71. Holotype ♀, China: Alaschan, (71 (descr.), depository: ZIN)*.
Ellampus heros : Bischoff 1913: 8 (China, cat.).
Chrysellampus heros : Semenov-Tian-Shanskij 1932: 5 (descr., typus gen.).
Omalus (Chrysellampus) heros : Linsenmaier 1959: 22 (typus subgen.).
Philoctetes heros : Kimsey and Bohart 1991: 255 (China: Alaschan, cat.).

######## Distribution.

China (Inner Mongolia).

####### 75. Philoctetes horvathi

Taxon classificationAnimaliaHymenopteraChrysididae

(Mocsáry, 1889)

http://species-id.net/wiki/Ellampus_horvati

Plate 23

Ellampus wesmaeli Mocsáry, 1882: 27. Lecctotype ♀ design. by Móczár (1964a: 434), Hungary (27 (descr.), 80 (cat.) depository: HNHM)*, *nec* Chevrier, 1862. (!) Mocsáry, 1889: 82. Replacement name for *Ellampus wesmaeli* Mocsáry, 1882.
Ellampus horvathii (!): Dalla Torre 1892: 13 (cat.).
Omalus (Omalus) horvathi : Linsenmaier 1959: 15 (key, *horwathi* (!)), 19 (China, tax., *pusillus* group); Linsenmaier 1997a: 249 (tax., *pusillus* group).
Omalus horváthi (!): Móczár 1964a: 434 (lectotype designation).
Philoctetes horvathi : Kimsey and Bohart 1991: 256 (cat.); Rosa et al. 2013: 13 (China, cat., distr.).

######## Material examined.

1 ex., Nan-Chan le Kan Tchegu à Lan Tcheou Dr. Vaillant 1909 / 2000 à 4000m Juillet 1908 (NMLS).

######## Distribution.

China (Shanxi). Widely distributed in the Palaearctic Region from Europe and North Africa to Korea (Kimsey and Bohart 1991; Linsenmaier 1997a).

####### 76. Philoctetes mongolicus

Taxon classificationAnimaliaHymenopteraChrysididae

(du Buysson, 1901), status revived

http://species-id.net/wiki/Ellampus_horvathi_mongolicus

Ellampus horvathi var. mongolicus du Buysson, 1901b: 98. Syntypes ♂♀, N Mongolia (98 (descr.), depository: NHMW)*.
Ellampus horwathi (!) var. mongolicus : du Buysson 1911: 219 (China: Nan Chan, Che Yeou Ho, tax.); Bischoff 1913: 8 (cat.).
Notozus mongolicus : Tsuneki 1948a: 116 (China: Shanxi: Wutaishan, tax., distr.), 128 (China: Shanxi, cat.).
Omalus (Notozus) mongolicus : Linsenmaier 1959: 16 (key), 23 (cat., *ambiguus* group).
Philoctetes horvathi : Kimsey and Bohart 1991: 256 (cat.).

######## Distribution.

China (Shanxi). Widely distributed from Mongolia to Central Asia and southern Russia to Volga (Trautmann 1927).

######## Remarks.

*Philoctetes mongolicus* was often erroneously considered belonging to the genus *Elampus* (= *Notozus*) due to its elongated metanotal projection. However, the metanotal projection is also present in various *Philoctetes* species (e.g.: *Philoctetes putoni* (du Buysson)). Kimsey and Bohart (1991) placed *Philoctetes mongolicus* under *Philoctetes horvathi*, even if Tsuneki and Linsenmaier considered it as a valid species. Type examination has confirmed that *Philoctetes mongolicus* is indeed a valid species.

####### 77. Philoctetes mordvilkoi

Taxon classificationAnimaliaHymenopteraChrysididae

(Semenov-Tian-Shanskij, 1932), n. comb.

http://species-id.net/wiki/Ellampus_mordvilkoi

Plate 24

Ellampus mordvilkoi Semenov-Tian-Shanskij, 1932: 36. Holotype ♀, China: Xinjiang: Chotan, Sajbag (36 (descr.), depository: ZIN) *.
Pseudomalus mordilkoi (!): Kimsey and Bohart, 1991: 268 (China: Singkiang [= Xinjiang]: Chotan Sajbag, cat.).

######## Distribution.

China (Xinjiang).

######## Remarks.

*Ellampus mordvilkoi* shows the main characteristics of the genus *Philoctetes sensu* Kimsey and Bohart (1991). The gena is not bisected by the genal carina, the punctuation on the mesosoma is more distributed along the notauli and the anal margin of the last metasomal tergite has a distinct brownish transparent rim with a wide median notch. Therefore, we have moved this taxon into the genus *Philoctetes* Abeille.

####### 78. Philoctetes praeteritorum

Taxon classificationAnimaliaHymenopteraChrysididae

(Semenov-Tian-Shanskij, 1932)

http://species-id.net/wiki/Parellampus_praeteritorum

Plate 25

Parellampus praeteritorum Semenov-Tian-Shanskij, 1932: 7. Holotype ♀, China: Sichuan, Tadzinlu (7 descr.), depository: ZIN)*.
Philoctetes praeteritorum : Kimsey and Bohart, 1991: 257 (China: Setchuan [= Sichuan], cat.).

######## Distribution.

China (Sichuan).

###### 13. Genus *Pseudomalus* Ashmead, 1902

####### 79. Pseudomalus auratus

Taxon classificationAnimaliaHymenopteraChrysididae

(Linnaeus, 1758)

http://species-id.net/wiki/Sphex_aurata

Sphex aurata Linnaeus, 1758: 572. Holotype ♀, Europe (572 (descr.), depository: LSL).
Ellampus auratus f. maculatus du Buysson, 1887: Tsuneki 1946: 33 (Manchuria, tax.), 38 (résumé).
Ellampus auratus var. cupratus Mocsáry, 1889: 92. Holotype ♂ [not ♀], Dalmatia (92 (descr.), depository: HNHM)*.
Ellampus auratus var. cupratus : du Buysson 1911: 219 (China: Nan Chan, Kan Tchéou, oasis Chan Kin Hia, Tien Chouei Tsing, Koua Tchéou Kéou tsé, tax.).
Ellampus auratus f. nigridorsus Tsuneki, 1950a: 63. Syntypes ♂♀, Japan, Korea, Manchuria (63 (descr.), depository: NIAS).
Ellampus auratus f. nigridorsus : Tsuneki, 1953a: 54 (Manchuria: Tâshonshan, cat., distr.).
Omalus (Omalus) auratus ssp. nigridorsus : Linsenmaier 1968: 9 (Manchuria, tax.).
Omalus (Omalus) auratus : Linsenmaier 1959: 14 (key), 17 (tax., descr., *auratus* group), 196 (figs 1–4).
Pseudomalus auratus : Kimsey and Bohart 1991: 263 (fig. 84), 264 (cat., figs 85d, 85i); Kurzenko and Lelej 2007: 1004 (China, cat.).

######## Material examined.

Heilongjiang: 1 ex., Harbin, 18.VI.1944; 1 ex., 1.VIII.1945 Alin leg. (NMLS).

######## Distribution.

China (Heilongjiang, Shanxi). Widely distributed in the Holarctic Region, beeing introduced into the Nearctic Region by commerce, very likely with multiple introductions (Krombein 1959; Kimsey and Bohart 1991; Kurzenko and Lelej 2007).

######## Host.

*Pemphredon rugifer* Dahlbom (Hymenoptera, Crabronidae) (Tsuneki 1970a).

######## Biology.

The larval habits were studied by Tsuneki (1952).

######## Remarks.

Kimsey and Bohart (1991) considered Ellampus auratus f. nigridorsus Tsuneki as an invalid name. However, it is a valid name according to the ICZN.

####### 80. Pseudomalus conradti

Taxon classificationAnimaliaHymenopteraChrysididae

(Bischoff, 1910)

http://species-id.net/wiki/Ellampus_conradti

Ellampus conradti Bischoff, 1910: 437. Syntypes ♂♀, Chinese Turkestan [= Xinjiang]: Tochta Chon, Jarkand (437 (descr.), depository: MNHU)*.
Ellampus conradti : Bischoff 1913: 8 (Chinese Turkestan [= Xinjiang], cat.).
Pseudomalus conradti : Kimsey and Bohart 1991: 267 (China, Singkiang [= Xinjiang], cat.).

######## Distribution.

China (Xinjiang).

####### 81. Pseudomalus corensis

Taxon classificationAnimaliaHymenopteraChrysididae

(Uchida, 1927)

http://species-id.net/wiki/Philoctetes_corensis

Philoctetes punctatus var. corensis Uchida, 1927: 153. Holotype ♂, Korea: Seiryori (153 (descr.), depository: EIHU).
Philoctetes corensis : Uchida 1933: 1 (cat.).
Ellampus corensis : Tsuneki 1946: 33 (China: Shanxi, tax.); Tsuneki 1948a: 120 (type series: China: Shanxi: Takui, Nanpintsun, Henglingshan - Peihungkaokao, Wutaishan, tax., comp. notes, descr.), 128 (Shanxi, cat.); Tsuneki 1953a: 55 (North China, Manchuria [= Heilongjiang]: Harbin, Kaiyüan; Manchuria, tax., distr.); Tsuneki 1953b: 22 (North China, Manchuria, tax., aberr.).
Omalus (Omalus) corensis : Linsenmaier 1959: 17 (possible syn. of *Omalus joannisi* (du Buysson, 1908), *auratus* group).
Pseudomalus corensis : Kimsey and Bohart 1991: 268 (synonym of *Pseudomalus punctatus* (Uchida, 1927)).

######## Distribution.

China (Heilongjiang, Liaoning, Shanxi). Korea (Uchida, 1927).

######## Remarks.

Kimsey and Bohart (1991) placed Philoctetes punctatus var. corensis Uchida in the junior synonymic list of *Pseudomalus punctatus* (Uchida, 1927). However, Tsuneki examined the material collected by Uchida and considered *Pseudomalus corensis* a valid species. We follow the latter interpretation.

####### 82. Pseudomalus hypocritus

Taxon classificationAnimaliaHymenopteraChrysididae

(du Buysson, 1893), n. comb.

http://species-id.net/wiki/Ellampus_hypocrita

Ellampus hypocrita du Buysson, 1893: 246. Syntype ♀, Mongolia [= China]: Kansu-Yelisyn-Kuse (246 (descr.), depositories: ISEA-PAS, MNHN)*.
Ellampus hypocrita : du Buysson 1911: 218 (China: Nan Chan, Chan Kin Hia, Tien Chouei Tsing, Koua Tchéou Kéou tsé, tax.); Bischoff 1913: 8 (Mongolia [= China], cat.).
Omalus hypocritus : Kimsey and Bohart 1991: 248 (cat.).

######## Distribution.

China (Gansu, Shanxi).

######## Remarks.

After the examination of the syntype specimen in the Radoszkowski collection in ISEA-PAS, we have confirmed that the species belongs to the genus *Pseudomalus* Ashmead *sensu* Kimsey and Bohart (1991).

####### 83. Pseudomalus joannisi

Taxon classificationAnimaliaHymenopteraChrysididae

(du Buysson, 1908)

http://species-id.net/wiki/Ellampus_joannisi

Ellampus joannisi du Buysson, 1908: 207. Holotype ♀, China: Nanking [= Nanjing] (207 (descr.), depository: MNHN)*.
Ellampus joannisi : Bischoff 1913: 8 (Japan [= China], cat.); Tsuneki 1946: 32 (tax).
Omalus (Omalus) joannisi : Linsenmaier 1959: 17 (China, Manchuria [= Heilongjiang], tax., descr., comp. notes).
Pseudomalus joannisi : Kimsey and Bohart 1991: 268 (China: Nanking, cat.).

######## Material examined.

Heilongjiang: 2♀♀, Harbin, 15.VII.1943; 1♂, 1♀, id., 9.VII.1944; 1♀, id., 9.VII.1944; 1♀, id., 10.VII.1949; 3♂♂, 8♀♀, id., 25.VI.1950; 1♂, 1♀, id., 28.VII.1950; 2♂♂, id., 21.VIII.1950; 2 exx., id., 20.VII.1953 all the specimens were collected by Alin (NMLS).

######## Distribution.

China (Heilongjiang, Jiangsu). Korea (Kimsey and Bohart 1991).

####### 84. Pseudomalus sinensis

Taxon classificationAnimaliaHymenopteraChrysididae

(Tsuneki, 1947)

http://species-id.net/wiki/Ellampus_sinensis

Ellampus sinensis Tsuneki, 1947: 44. Holotype ♀; China: Beijing (44 (descr.), 45 (Beijing, comp. notes), depository: OMNH, not NIAS).
Ellampus sinensis f. viridiauratus Tsuneki, 1948a: 118. Syntypes ♂♀♀, China: Henglingshan-Peihungkaokao, Wutaishan, Yangchêng (118 (descr.), 119 (China: Shanxi: Yangchêng, Wutaishan, Henglingshan-Peihungkaokao, comp. notes), 128 (cat.), depository: KUM?).
Ellampus sinensis f. nigricans Tsuneki, 1948a: 119. Holotype ♂, China: Kiu-Taiyüan (119 (descr.), 128 (cat.), depository: KUM?).
Ellampus sinensis : Tsuneki 1948a: 128 (China: Beijing district, cat.).
Omalus (Omalus) sinensis : Linsenmaier 1959: 20 (China, tax., *pusillus* group).
Pseudomalus sinensis : Kimsey and Bohart 1991: 269 (China, cat.).

######## Distribution.

China (Beijing, Shanxi).

######## Remarks.

Kimsey and Bohart (1991) considered Ellampus sinensis f. nigricans Tsuneki and Ellampus sinensis f. viridiauratus Tsuneki as invalid names. However, the two names are valid according to the ICZN.

####### 85. Pseudomalus triangulifer

Taxon classificationAnimaliaHymenopteraChrysididae

(Abeille, 1877)

http://species-id.net/wiki/Omalus_triangulifer

Plate 26

Omalus triangulifer Abeille, 1877: 65. Lectotype ♀ design. by Kimsey (1986: 106); France: St. Baume (depository: MNHN)*.
Omalus (Omalus) triangulifer : Linsenmaier 1959: 14 (key), 17 (tax., descr.), 196 (figs 5, 6); Linsenmaier 1997a: 248 (China: Gansu, cat.).
Ellampus auratus triangulifer (!): Kimsey 1986: 106 (lectotype design.).
Pseudomalus triangulifer : Kimsey and Bohart 1991: 269 (cat.); Rosa 2006: 78 (cat.), 108 (key), 113 (China, tax., descr.).

######## Distribution.

China (Gansu). Widely distributed in the Palaearctic Region (Linsenmaier 1959, 1968, 1987, 1997a).

####### 86. Pseudomalus tshingiz

Taxon classificationAnimaliaHymenopteraChrysididae

(Semenov-Tian-Shanskij, 1954)

http://species-id.net/wiki/Ellampus_tshingiz

Plate 27

Ellampus tshingiz Semenov-Tian-Shanskij (in Semenov-Tian-Shanskji and Nikol’skaja), 1954: 93. Holotype ♂, Sandzhu [Xinjiang], Gushan Gobi (depository: ZIN)*.
Pseudomalus tshingiz : Kimsey and Bohart 1991: 264 (fig. 85a), 270 (cat.).

######## Distribution.

China (Xinjiang).

######## Remarks.

The type (according to the original description and labels) is from the Oasis Sandzhu [Xinjiang], Gushan Gobi and not from [Kansu]: Sachow Gobi (as reported in Kimsey and Bohart 1991).

####### 87. Pseudomalus violaceus

Taxon classificationAnimaliaHymenopteraChrysididae

(Scopoli, 1763)

http://species-id.net/wiki/Sphex_violacea

Sphex violacea Scopoli, 1763: 298. Holotype ♀, Italy: Trieste (298 (descr.), depository: lost).
Ellampus violaceus f. virens Mocsáry, 1889: Tsuneki 1953a: 54 (cit.).
Ellampus violaceus : Tsuneki 1953a: 54 (Manchuria [= Inner Mongolia]: Hairar, tax., distr.).
Omalus (Omalus) violaceus : Linsenmaier 1959: 14 (key), 17 (tax., descr., *auratus* group); Linsenmaier 1997b: 31 (key), 48 (Manchuria, tax., descr., fig. 16).
Omalus violaceus : Móczár 1967: 30 (Manchuria, key, tax., descr., distr.); Banaszak 1980: 9 (tax., biol., distr.).
Pseudomalus violaceus : Kimsey and Bohart 1991: 264 (fig. 85g), 270 (cat.).

######## Distribution.

China (Inner Mongolia). Widely distributed in the Palaearctic Region (Kimsey and Bohart 1991).

######## Remarks.

The identification by Tsuneki (1953a) should be double-checked. The specimen may be related to *Pseudomalus bergi* Semenov-Tian-Shanskij, 1932.

##### Tribe Chrysidini

###### 14. Genus *Chrysidea* Bischoff, 1913

####### 88. Chrysidea pumila

Taxon classificationAnimaliaHymenopteraChrysididae

(Klug, 1845)

http://species-id.net/wiki/Chrysis_pumila

Chrysis pumila Klug, 1845: tab. 45, fig. 13. Type, Sudan: Ambukohl (type lost).
Chrysidea pumila Bischoff 1913: 34 (cat., typ. gen.); Hammer 1936: 3 (China [Xinjiang]: Urumchi [= Ürümqi], cat.); Kimsey and Bohart 1991: 314 (cat.).

######## Material examined.

2♀♀, Harare: Ketmen, Thian Chan Occid. Monts Sussamyr Ketmen Tjube M. Pic. 1914. The specimens were collected close to the Kyrgyzstan border (Sussamyr Mt.).

######## Distribution.

China (Xinjiang). Widely distributed in the Palaearctic Region (Kimsey and Bohart 1991).

###### 15. Genus *Chrysis* Linnaeus, 1761

####### 89. Chrysis aegle

Taxon classificationAnimaliaHymenopteraChrysididae

Semenov-Tian-Shanskij, 1967

http://species-id.net/wiki/Chrysis_aegle

Plate 28

Chrysis (Gonodontochrysis) aegle Semenov-Tian-Shanskij, 1967: 160. Holotype ♀, North China: Alashan, Maladzhin (depository: ZIN)*.
Chrysis aegle : Kimsey and Bohart 1991: 379 (Mongolia [= Inner Mongolia]: Alashan, cat.).

######## Distribution.

China (Inner Mongolia).

######## Remarks.

*Chrysis aegle* belongs to the *bihamata* group.

####### 90. Chrysis alticata

Taxon classificationAnimaliaHymenopteraChrysididae

Bohart, 1991

http://species-id.net/wiki/Chrysis_alticata

Chrysis (Tetrachrysis) alticola Mocsáry, 1914: 42. Holotype ♀, Tibet: Gyangtse, 13.000 ft. (depository: BMNH) *nec*Semenov-Tian-Shanskij 1912.
Chrysis alticata Bohart (in Kimsey and Bohart), 1991: 381. Replacement name for *Chrysis alticola* Mocsáry, 1914, *nec*Semenov-Tian-Shanskij 1912 (*ignita* group).
Chrysis alticata : Kurzenko and Lelej 2007: 1005 (China, cat.).

######## Distribution.

China (Tibet).

####### 91. Chrysis angolensis

Taxon classificationAnimaliaHymenopteraChrysididae

Radoszkovsky, 1881

http://species-id.net/wiki/Chrysis_angolensis

Chrysis fuscipennis Brullé, 1846: 38. Holotype ♀, Philippine (depository: MNHN), *nec* Dahlbom, 1829.
Chrysis janthinus (!) Smith, 1874b: 459. Holotype ♀, China: Shanghai (459 (descr.), depository: BMNH) *nec* Förster, 1853.
Chrysis angolensis Radoszkovsky, 1881: 219. Holotype, Angola (219 (descr.), type lost ?).
Chrysis erratica du Buysson, 1887: 189. Syntypes ♂♀, China, Egypt (189 (descr.), depository: MNHN?) (synonymised by Mocsáry 1889: 370).
Chrysis (Tetrachrysis) janthina : Mocsáry 1889: 374 (China, descr.); Bischoff 1913: 54 (North China, cat.).
Chrysis janthina : Dalla Torre 1892: 66 (China, cat.).
Chrysis fuscipennis : du Buysson (in André) 1895: 443 (China, tax., descr., distr.); du Buysson 1898a: 529 (China: Kiang-si, North Beijing, cat., distr.); du Buysson 1899: 165 (China, cat., distr.); du Buysson 1900: 153 (China: Beijing, cat.); du Buysson 1901b: 101 (Central China, cat.); Bingham 1903: 386 (key), 467 (tax., descr.), 468 (China, distr.); Linsenmaier 1959: 149 (*fuscipennis* group).
Chrysis (Tetrachrysis) fuscipennis : Mocsáry 1913b: 614 (Taiwan); Uchida 1927: 151 (Taiwan, cat.); Tsuneki 1947: 55 (China: Beijing, Taiwan, cat., distr.); Tsuneki 1948a: 125 (Manchuria: Yangchêng, Taiwan, tax.), 128 (Beijing distr., Manchuria, Taiwan, cat.); Tsuneki 1953a: 59 (China: Beijing, Formosa, cat., distr.); Tsuneki 1953b: 26 (North China, tax., distr.). Tsuneki 1970b: 13 (Taiwan: Ilan Province: Tsukeng, Chuantou; Taipei Province: Ulai, Kueishanlu; Nantou Province: Puli; Chiai Province: Chuchi; Taitung Province: Chihpenchi; Pingtung Province: Ssuchungchi, Fanshanlu, Manchou, cat.).
Chrysis fuscipennis var. murasaki Uchida, 1927: 155. Syntypes ♂♂♀♀, Japan, Korea (155 (descr.), depository: EIHU).
Tetrachrysis fuscipennis : Hammer 1950: 2 (China: Kiangsu [= Jiangsu], cat.).
Chrysis fuscipennis var. takanoi Tsuneki, 1950: 78. Holotype ♀, Taiwan: Shinka (78 (descr.), depository: NIAS).
Chrysis (Tetrachrysis) fuscipennis murasaki : Tsuneki 1953a: 59 (Manchuria: Sungari, Kunchun, cat., distr.); Tsuneki 1953b: 26 (North China, Manchuria, tax., distr.).
Chrysis (Chrysis) fuscipennis murasaki : Linsenmaier 1959: 149 (North China, Manchuria, descr., distr.); Tsuneki 1961: 376 (Manchuria, cat.).
Chrysis (Chrysis) fuscipennis fuscipennis : Tsuneki 1970c: 48 (Taiwan, key, tax.).
Chrysis angolensis : Kimsey and Bohart 1991: 319 (tax.), 336 (110), 337 (tax.), 357 (fig. 113c), 383 (cat., *angolensis* group); Kurzenko and Lelej 2007: 1005 (China, cat.); Terayama et al. 2010: 3 (China, cat.), 4 (figs.), 12 (tab., biol.).

######## Material examined.

1♀, Museum Paris Checkiang Hangtchéou A. Pichon 1925; 1♀, China Ning Po V.36 Coll. Linsenmaier; 1♀, Museum Paris Kouy-Tchéou Cavalerie 1921; 1♀, Museum Paris Chine Nan-King J. de Joannis 1908; 1♀, N Formosa Chipon Aug. 1935 K. Iwata, all the specimens identified by Linsenmaier in 1959 as *Chrysis fuscipennis* Brullé. The following specimens were identified by Linsenmaier in 1974 and 1979 as *Chrysis fuscipennis murasaki* Uchida: 2 ♀♀, Mandschurei Maoerschan, 20.–30.VII.1939; 1♀, Mandschurei Maoerschan, 5.8.40; 1♂ and 10♀♀, Maoerschan, 5–10.VIII.1939 Mandschurei; 11♀♀, China: Manchuria. Maoershan 100 Km E. Harbin on Chinese Eastern Railway, 18.VIII.1941 V.N. Alin, Coll.; 2♂♂, Chusan China Juni 1948 Collect. Naef; 5♀♀, Mandschurei Charbin 9.VII.1944 W. Alin leg (all specimens in NMLS). 1♀, Sichuan, Tzitun, 16.IX.1893, leg. Potanin (ZIN); 1♀, Nin-sia-fu, Yellow river valley, 4.–16.VI.1908, leg. Kozlov (ZIN); 2♀♀, Lyu-li-he, 65 km SW Beijing, 14.–17.VIII.1913, leg. Vasiliev (ZIN); 1♀, Harbin, 19.VI.1911, leg. Emelyanov (ZIN); Imanpo (Manchuria), 20.VI.1911, leg. Emelyanov (ZIN); 1♀, env. Beijing, VII.1916 (ZIN).

######## Distribution.

China (Heilongjiang, Jilin, Beijing, Shanxi, Shanghai, Jiangsu, Zhejiang, Jiangxi, Taiwan, Guizhou, Sichuan). World-wide except Europe (Kimsey and Bohart 1991).

######## Remarks.

Hosts are *Chalybion japonicum*, *Sceliphron mandraspatanum*, and *Sceliphron deforme* (Hymenoptera, Sphecidae) (Tereyama et al. 2010).

####### 92. Chrysis angustula

Taxon classificationAnimaliaHymenopteraChrysididae

Schenck, 1856

http://species-id.net/wiki/Chrysis_angustula

Chrysis angustula Schenck, 1856: 30. Lectotype ♀ design. by Morgan (1984: 9), Germany: former Duchy of Nassau (depository: SMFD).
Chrysis (Chrysis) angustula : Linsenmaier 1959: 151 (key), 159 (tax., descr., *ignita* group), 217 (fig. 697).
Chrysis angustula : Kimsey and Bohart 1991: 383 (cat., *ignita* group).
Chrysis angustula gracilis : Linsenmaier 1997b: 124 (China, Manchuria, tax., descr., *ignita* group).

######## Material examined.

1♀, Sjaolin [= Henan: Shaolin], 25.VIII.1940; Heilongjiang: 1♀, Harbin, 9.VII.1944, leg. Alin; 1♀, Harbin, 25.VII.1950 leg. Alin; 2♀♀, Maoershan, 100 Km E Harbin on Chinese Eastern Railway, 18.VIII.1941 V.N. Alin, coll.; all the specimens identified by Linsenmaier and Niehuis in 1998 as *Chrysis angustula* Schenck.

######## Distribution.

China (Heilongjiang, Jilin, Henan). Europe and Siberia (Kimsey and Bohart 1991).

####### 93. Chrysis asahinai

Taxon classificationAnimaliaHymenopteraChrysididae

Tsuneki, 1950

http://species-id.net/wiki/Chrysis_asahinai

Chrysis asahinai Tsuneki, 1950a: 80. Holotype ♀, Manchuria (80 (descr.), depository: OMNH).
Chrysis asahinai : Tsuneki 1953a: 59 (Manchuria: Ouri near Liaoyüanchow, cat.), 60 (figs 2A, 2B); Kimsey and Bohart 1991: 385 (cat., *pulchella* group); Kurzenko and Lelej 2007: 1005 (Northeast China, cat.).
Chrysis (Chrysis) asahinai : Linsenmaier 1959: 103 (Manchuria, key, tax., *pulchella* group).

######## Distribution.

China (Liaoning).

####### 94. Chrysis buda

Taxon classificationAnimaliaHymenopteraChrysididae

Bohart, 1991

http://species-id.net/wiki/Chrysis_buda

Plate 29

Chrysis (Tetrachrysis) buddhae Semenov-Tian-Shanskij, 1967: 179. Holotype ♀, North China: Inner Mongolia (179 (descr.), depository: ZIN)* *nec* Mocsáry, 1913a.
Chrysis buda Bohart (in Kimsey and Bohart), 1991: 392. Replacement name for *Chrysis buddhae* Semenov-Tian-Shanskij, 1967 *nec* Mocsáry, 1913a (cat., *ignita* group).
Chrysis buda : Kurzenko and Lelej 2007: 1005 (China: Hubei, cat.).

######## Distribution.

China (Inner Mongolia, Hubei).

######## Remarks.

The specimen labeled as type was collected on the 20.V.1908 by Kozlov (and not 20.VI.1908 as written in the original description) in Alashan, Tzosto Canyon and not Gansu.

####### 95. Chrysis buddhae

Taxon classificationAnimaliaHymenopteraChrysididae

Mocsáry, 1913

http://species-id.net/wiki/Chrysis_buddhae

Plate 30

Chrysis (Hexachrysis) buddhae Mocsáry, 1913a: 25. Lectotype ♂, design. by Bohart (in Bohart and French 1986: 341), Taiwan: Takao [= Kaohsiung] (depository: HNHM)*.
Chrysis (Hexachrysis) buddhae : Mocsáry 1913b: 619 (China, Taiwan: Takao, cat.); Bischoff 1913: 64 (China, Taiwan, cat.); Uchida 1927: 152 (China, Taiwan, cat.); Tsuneki 1963a: 1 (China, Taiwan, key), 5 (tax., comp. notes), 6 (China, Taiwan, comp. notes).
Chrysis buddhae : Kimsey and Bohart 1991: 392 (Taiwan, cat., *smaragdula* group).

######## Material examined.

1♀, Taiwan, Taihanroku, 8–18.IV.1908, leg. H. Sauter, *Chrysis buddhae* Semenov det. Linsenmaier 1973 (NMLS) [Linsenmaier confused the name of the authors].

######## Distribution.

China (Taiwan). Borneo, India (Kimsey and Bohart 1991).

######## Remarks.

The specimen listed as Chrysis (Hexachrysis) buddhae by Uchida (1927) was considered as *Chrysis takasago* Tsuneki, 1963 (Tsuneki 1963a).

####### 96. Chrysis carnifex

Taxon classificationAnimaliaHymenopteraChrysididae

Mocsáry, 1889

http://species-id.net/wiki/Chrysis_carnifex

Plate 31

Chrysis (Tetrachrysis) carnifex Mocsáry, 1889: 517. Holotype ♂, China: Ta-tschiansy (517 (descr.), depository: HNHM)*.
Chrysis (Tetrachrysis) carnifex : Mocsáry 1890a: 63 (China borealis, cat.); Bischoff 1913: 49 (North China, cat.); Uchida 1927: 151 (North China, cat.); Tsuneki 1948a: 125 (China, cat., synonym of *Chrysis chrysochlora* Mocsáry, 1889); Tsuneki 1948b: 48 (North China: Shanxi, tax.).
Chrysis carnifex : Dalla Torre 1892: 49 (China, cat.); Kimsey and Bohart 1991: 394 (China, cat., *ignita* group); Kurzenko and Lelej 2007: 1005 (China, cat.).

######## Distribution.

China (Shanxi).

######## Remarks.

The type of Chrysis (Tetrachrysis) carnifex shares most of the characteristics with the type of *Chrysis keriensis* Mocsáry, which is the male of *Chrysis chrysochlora* Mocsáry. The main difference is found in the punctuation on the mesosoma and on the first two metasomal tergites. Tsuneki (1948a, 1948b) considered Chrysis (Tetrachrysis) carnifex Mocsáry as a junior synonym of *Chrysis chrysochlora* Mocsáry, 1889.

####### 97. Chrysis cavaleriei

Taxon classificationAnimaliaHymenopteraChrysididae

(du Buysson, 1908)

http://species-id.net/wiki/Tetrachrysis_cavalerieri

Plate 32

Chrysis cavaleriei *Tetrachrysis Cavaleriei* du Buysson, 1908: 211. Holotype ♀, China: Kouy-Tchéou [= Guizhou]: Kouy-Yang (211 (descr.), depository: MNHN)*.
Chrysis (Tetrachrysis) cavaleriei : Bischoff 1913: 49 (China, cat.); Tsuneki 1953b: 27 (Middle China [= Guizhou]: Kouy-Yang, tax., descr.).
Chrysis (Chrysis) cavaleriei : Linsenmaier 1959: 112 (China, descr., distr., *succincta* group), 205 (fig. 422); Linsenmaier 1968: 69 (China, cat.).
Chrysis cavaleriei : Kimsey and Bohart 1991: 394 (China, cat., *succincta* group); Kurzenko and Lelej 2007: 1005 (China, cat.); Terayama et al. 2010: 8 (China, cat.).

######## Distribution.

China (Guizhou). Korea (Tsuneki 1953b).

####### 98. Chrysis ceciliae

Taxon classificationAnimaliaHymenopteraChrysididae

du Buysson, 1904

http://species-id.net/wiki/Chrysis_ceciliae

Plate 33

Chrysis ceciliae *Chrysis Ceciliae* du Buysson, 1904: 259. Holotype ♀, Java: Malang (259 (descr.), depository: MNHN)*.
Chrysidea (Chrysogona) insulicola Mocsáry, 1913b: 614. Holotype ♀, Taiwan: Takao (614 (descr.), 619 (Taiwan, cat.), depository: HNHM)* (synonymised by Kimsey and Bohart 1991: 395).
Chrysis (Chrysogona) insulicola : Uchida 1927: 151 (Taiwan, cat.); Uchida 1933: 2 (Taiwan, cat.).
Chrysis (Chrysura) insulicola : Tsuneki 1970b: 6 (Taiwan: Ssuchungchi, Taoyeh, tax., descr., figs 5–8).
Chrysis ceciliae : Kimsey and Bohart 1991: 321 (fig. 104b), 329 (fig. 107f), 336 (fig. 110f), 339 (tax., *ceciliae* group), 395 (Taiwan, cat., *ceciliae* group).

######## Distribution.

China (Taiwan). Java, Philippines, Malaysia, Laos (Kimsey and Bohart 1991).

######## Remarks.

Tsuneki (1970b) redescribed Chrysidea (Chrysogona) insulicola Mocsáry.

####### 99. Chrysis chinensis

Taxon classificationAnimaliaHymenopteraChrysididae

Mocsáry, 1912

http://species-id.net/wiki/Chrysis_chinensis

Plate 34

Chrysis (Tetrachrysis) ignita var. chinensis Mocsáry, 1912b: 589. Holotype ♀, China: Shanghai (589 (descr.), depository: HNHM)*.
Chrysis (Tetrachrysis) ignita var. chinensis Bischoff, 1913: 53 (China, cat.); Trautmann 1927: 147 (China: Shanghai, cat.).
Chrysis (Chrysis) chinensis : Linsenmaier 1959: 158 (China, Manchuria, tax., descr., distr., *ignita* group), 205 (fig. 393); Linsenmaier 1997b: 39 (key), 113 (China, Manchuria, cat., descr., distr., *ignita* group, fig. 96).
Chrysis chinensis : Kimsey and Bohart 1991: 396 (China: Shanghai, cat., *ignita* group); Tarbinsky 2000: 195 (key), 202 (China, cat., distr.); Kurzenko and Lelej 2007: 1005 (China, cat.).

######## Material examined.

Heilongjiang: 1♀, Harbin, 25.VI.1950 leg. Alin; 2♂♂, Harbin, 20.VII.1953, leg. Alin; 1♀, Harbin, 24.VI.1953 leg. Alin. All the specimens identified by Linsenmaier in 1959 (NMLS).

######## Distribution.

China (Helongjiang, Shanghai).

####### 100. Chrysis chrysochlora

Taxon classificationAnimaliaHymenopteraChrysididae

Mocsáry, 1889

http://species-id.net/wiki/Chrysis_chrysochlora

Plate 35

Chrysis (Tetrachrysis) chrysochlora Mocsáry, 1889: 589. Lectotype ♀ design. by Bohart (in Kimsey and Bohart 1991: 396), Uzbekistan: Tashkent (depository: HNHM)*.
Chrysis viridans Radoszkowski, 1891: 192. Holotype ♀, Turkmenistan, Ashkabad (192 (descr.), depository: ISEA-PAS).
Chrysis (Tetrachrysis) chrysochlora : Bischoff 1910: 483 (Chinese Turkestan [= Xinjiang]: Chotan-Kiljang, cat.); Tsuneki 1948a: 125 (China: Shanxi: Hengshuichen-Henglingkuan, cat.), 126 (tax., comp. notes, distr.), 128 (cat.).
Chrysis (Chrysis) chrysochlora : Linsenmaier 1959: 152 (key), 161 (China, tax., descr., distr., *ignita* group).
Chrysis chrysochlora : Kimsey and Bohart, 1991: 396 (cat.); Tarbinsky 2000: 193 (key), 197 (China, cat., distr.); Rosa et al. 2013: 17 (China, cat., distr.).

######## Material examined.

7♂♂ and 10♀♀ collected at: Alashan, Din-yuan-in, 3.–5.V.1908, 14.–28.V.1908, 31.V.1908, 3.VI.1908 and 23.–26.IV.1909, leg. Kozlov (ZIN); 1♀, Huan-He Valley, 6.–20.X.1908, leg. Kozlov (ZIN); 1♀, Gansu, Pin-fan, 21.–23.VIII.1908, leg. Kozlov; 1♀, env. Sinin, 1.IX.1908, leg. Kozlov (ZIN). All the specimen identified as *Chrysis viridans*.

######## Distribution.

China (Xinjiang, Inner Mongolia, Gansu, Shanxi). Turkmenistan (Radoszkowski, 1891), Iran, Lebanon, Turkey, Turkmenistan (Linsenmaier 1959, 1968), Uzbekistan (Tarbinsky 2000).

####### 101. Chrysis comta

Taxon classificationAnimaliaHymenopteraChrysididae

Förster, 1853

http://species-id.net/wiki/Chrysis_comta

Chrysis comta Förster, 1853: 314. Holotype ♂, Turkey (314 (descr.), depository: lost).
Chrysis (Tetrachrysis) ignita f. comta : Tsuneki 1948a: 126 (cat.), 127 (China: Shanxi: Luan-hsien, Nanpintsun, distr.), 128 (Shanxi, cat.).
Chrysis (Chrysis) comta : Linsenmaier 1959: 151 (key), 152 (key), 158 (Manchuria, tax., descr., distr., *ignita* group), 208 (fig. 500).
Chrysis comta : Móczár 1967: 111 (key, tax., descr.), 112 (Manchuria, distr. ); Kimsey and Bohart 1991: 399 (cat., *ignita* group).

######## Material examined.

Heilongjiang: 2♂♂, Harbin, 31.V.1943 leg. Alin; 1♂, Harbin, 20.VII.1953.

######## Distribution.

China (Helongjiang, Shanxi). Europe, southern Russia (Linsenmaier, 1959).

####### 102. Chrysis consobrina

Taxon classificationAnimaliaHymenopteraChrysididae

Mocsáry, 1889, status revived

http://species-id.net/wiki/Chrysis_consobrina

Plate 36

Chrysis consobrina Mocsáry, 1889: 458. Lectotype ♀ design. by Bohart (in Bohart and French 1986: 341), Transcaspia (depository: HNHM)*.
Chrysis scutellaris ssp. consobrina : Semenov-Tian-Shanskij and Nikol’skaja 1954: 127 (China: Xinjiang, tax., descr.).
Chrysis (Chrysis) soror ssp. consobrina : Linsenmaier 1959: 125 (tax., descr., distr., *scutellaris* group).
Chrysis soror : Kimsey and Bohart 1991: 464 (cat., *scutellaris* group).

######## Distribution.

China (Xinjiang).

######## Remarks.

*Chrysis consobrina* was considered a subspecies of *Chrysis scutellaris* Fabricius by Semenov-Tian-Shanskij and Nikolskaja (1954), and later, a subspeceis of *Chrysis soror* Dahlbom (Linsenmaier 1959), the eastern greenish form of *Chrysis scutellaris*. For this reason Kimsey and Bohart (1991) placed it in synonym of *Chrysis soror*. However the type examination of *Chrysis consobrina* confirms that it is a valid species because it shares only a similar colour with *Chrysis scutellaris* and *Chrysis soror*.

####### 103. Chrysis dentipes

Taxon classificationAnimaliaHymenopteraChrysididae

Radoszkowski, 1877

http://species-id.net/wiki/Chrysis_dentipes

Chrysis dentipes Radoszkowski, 1877: 15. Lectotype ♀ design. by Bohart (in Kimsey and Bohart 1991: 403, Uzbekistan: Sarafschan (depository: MMU)*.
Chrysis eversmanni Mocsáry, 1912a: 407. Holotype ♂, Turkestan (407 (descr.), depository: HNHM) (synonymised by Kimsey and Bohart 1991: 403).
Chrysis (Cornuchrysis) eversmanni : Linsenmaier 1968: 115 (China, tax., *taczanovskii* group).
Chrysis dentipes : Kimsey and Bohart 1991: 403 (cat., *taczanovskii* group).

######## Material examined.

1♂, 2♀♀: Harare Ketmen, Tjube, Thian Chan Occid. Monts Sussamyr leg. Pic 1914, *Chrysis eversmanni* Mocs. det. Linsenmaier 1973 (NMLS).

######## Distribution.

China (Xinjiang). Iran, Tadjikistan, Turkmenistan, Uzbekistan (du Buysson 1900; Mocsáry, 1912a; Semenov-Tian-Shanskij, 1954; Kimsey and Bohart 1991; Tarbinsky 2002a).

####### 104. Chrysis duplopilosa

Taxon classificationAnimaliaHymenopteraChrysididae

Linsenmaier, 1968

http://species-id.net/wiki/Chrysis_duplopilosa

Chrysis (Chrysis) duplopilosa Linsenmaier, 1968: 101. Holotype ♀, Tibet: Gyangtse (101 (descr.), *ignita* group, depository: BMNH).
Chrysis duplopilosa : Kimsey and Bohart 1991: 406 (Tibet: Gyangtse, cat., *ignita* group).

######## Distribution.

China (Tibet).

####### 105. Chrysis durga

Taxon classificationAnimaliaHymenopteraChrysididae

Bingham, 1903

http://species-id.net/wiki/Chrysis_durga

Plate 37

Chrysis durga Bingham, 1903: 487. Lectotype ♀ design. by Bohart (in Kimsey and Bohart 1991: 406), Burma: Mandalay (depository: BMNH)*.
Chrysis durga : Kimsey and Bohart 1991: 406 (China, lectotype design., cat., *smaragdula* group).

######## Distribution.

China. Burma, Laos, Malaysia (Kimsey and Bohart 1991).

####### 106. Chrysis extersa

Taxon classificationAnimaliaHymenopteraChrysididae

du Buysson, 1898

http://species-id.net/wiki/Chrysis_extersa

Chrysis extersa du Buysson, 1898b: 137. Holotype ♀ China: Nyan-kin [= Nanjing] (137 (descr.), depository: MNHN).
Chrysis extersa : Kimsey and Bohart 1991: 410 (China: Nyan-kin [= Nanjing], cat., *ignita* group); Kurzenko and Lelej 2007: 1005 (China, cat.).

######## Distribution.

China (Jiangsu).

####### 107. Chrysis fasciata daphne

Taxon classificationAnimaliaHymenopteraChrysididae

Smith, 1874

http://species-id.net/wiki/Chrysis_daphne

Chrysis fasciata daphne *Chrysis Daphne* Smith, 1874a: 399. Holotype ♀ Japan: Hiogo (399 (descr.), depository: BMNH).
Chrysis (Hexachrysis) zetterstedti Dahlbom, 1845: Tsuneki 1947: 57 (China: Beijing, cat., distr.) [misid.].
Chrysis (Tetrachrysis) daphne : Tsuneki 1948b: 48 (tax.).
Chrysis (Hexachrysis) fasciata zetterstedti : Tsuneki 1953a: 60 (Manchuria: Tashonshan [= Guansu, Daxiangshan], tax., distr.) [misid.]; Tsuneki 1953b: 28 (North China, tax., distr.) [misid.].
Chrysis (Chrysis) fasciata var. daphne : Linsenmaier 1959: 163 (China (?), tax., descr., *fasciata* group).
Chrysis (Hexachrysis) fasciata daphne : Tsuneki 1963a: 1 (Manchuria, North China, key), 8 (Manchuria, North China, Beijing, tax., descr.), 9 (comp. notes, figs 16–23).
Chrysis (Pyria) fasciata daphne : Tsuneki 1970c; 49 (North China, key, tax.).
Chrysis fasciata : Kimsey and Bohart 1991: 410 (*smaragdula* group, cat.); Kurzenko and Lelej 2007: 1005 (Northeast China, cat.); Terayama et al. 2010: 4 (figs.), 8 (China, cat.), 12 (tab., biol.), 13 (fig. 2), 14 (fig. 7).

######## Material examined.

Heilongjiang: 1♀, Harbin, 1.VIII.1943 leg. Alin, *Chrysis fasciata zetterstedti* Dhlb. det. Linsenmaier 1974 (NMLS).

######## Distribution.

China (Heilongjiang, Gansu, Beijing). Japan (Smith 1874a; Linsenmaier 1959).

######## Remarks.

At present, Chrysis fasciata var. zetterstedti Dahlbom, 1845 is known for certain only in Scandinavian countries and the Baltic countries. The Chinese and Japanese specimens should be considered as Chrysis fasciata ssp. daphne. Unpublished molecular data confirms that *zetterstedti* and *daphnis* belong to separate clades of the typical *Chrysis fasciata fasciata* (Paukkunen et al. 2014).

####### 108. Chrysis foochowia

Taxon classificationAnimaliaHymenopteraChrysididae

Linsenmaier, 1968

http://species-id.net/wiki/Chrysis_foochowia

Chrysis (Chrysis) foochowia Linsenmaier, 1968: 102. Holotype ♀, China: Foochow [Fujian] (102 (descr., *ignita* group), depository: BMNH).
Chrysis foochowia : Kimsey and Bohart 1991: 411 (China, cat., *ignita* group); Kurzenko and Lelej 2007: 1005 (Southeast China, cat.).

######## Material examined.

Fujian: 1♀, China Foochow C.R. Kellogg / Type ♀ *Chrysis* L. *foochowia* Lins. det. Linsenmaier 1966.

######## Distribution.

China (Fujian).

####### 109. Chrysis fossulata

Taxon classificationAnimaliaHymenopteraChrysididae

Smith, 1874

http://species-id.net/wiki/Chrysis_fossulata

Chrysis fossulatus (!) Smith, 1874b: 459. Holotype ♀, China: Shanghai (459 (descr.), depository: BMNH).
Chrysis (Tetrachrysis) fossulata : Mocsáry 1889: 375 (China septentrionalis, tax., descr.); Bischoff 1913: 51 (North China, cat.).
Chrysis fossulata : Dalla Torre 1892: 62 (China borealis, cat.); Kimsey and Bohart 1991: 412 (China, cat., *intricans* group).

######## Distribution.

China (Shanghai). Neotropical species distributed from Brazil to Venezuela (Kimsey and Bohart 1991).

######## Remarks.

According to Kimsey and Bohart (1991), *Chrysis fossulata* was introduced to China, South Africa and Australia via commerce.

####### 110. Chrysis fouqueti

Taxon classificationAnimaliaHymenopteraChrysididae

du Buysson, 1908

http://species-id.net/wiki/Chrysis_fouqueti

Chrysis fouqueti *Chrysis Fouqueti* du Buysson, 1908: 210. Holotype ♀, Viet Nam: “Tonkin” (210 (descr.descr.), depository: MNHN)*.
Chrysis (Tetrachrysis) faceta Mocsáry, 1912b: 561. Holotype ♂, Taiwan: Takao [= Kaohsiung], (561 (descr.), depository: HNHM) *nec* Aaron, 1885.
Chrysis (Tetrachrysis) faceta : Mocsáry 1913b: 615 (Taiwan: Takao [= Kaohsiung], Taihorin, Taihorinsho, cat.), 619 (Taiwan, cat.); Bischoff 1913: 51 (Taiwan, cat.); Uchida 1927: 151 (Taiwan, cat.); Uchida 1933: 5 (Taiwan: Tainan, Shinka, cat.).
Chrysis (Chrysis) facetana Linsenmaier, 1968: 101. Replacement name for *Chrysis faceta* Mocsáry, 1912.
Chrysis (Chrysis) faceta : Tsuneki 1970b: 13 (Taiwan: Tsukeng, Chuantou, Fangliao, Fangshnlu, Paoli, tax., descr.), 14 (figs 35–37).
Chrysis fouqueti : Kimsey and Bohart 1991: 412 (Taiwan, cat., *ignita* group); Kurzenko and Lelej 2007: 1005 (Taiwan, cat.).

######## Material examined.

1♀, China Tinghai [= Qinghai], Chusom Coll. Linsenmaier / ♀ *Chrysis* L. *fouqueti* Buyss. det. Linsenmaier 1973.

######## Distribution.

China (Qinghai, Shandong, Taiwan). Mongolia, Viet Nam (du Buysson 1908; Mocsáry, 1912).

######## Remarks.

Linsenmaier (1959, 1968) considered *Chrysis csikiana* as a subspecies of *Chrysis fouqueti* from the Chinese mainland and *Chrysis facetana* to be a subspecies from Taiwan.

####### 111. Chrysis fulgida

Taxon classificationAnimaliaHymenopteraChrysididae

Linnaeus, 1761

http://species-id.net/wiki/Chrysis_fulgida

Chrysis fulgida Linnaeus, 1761: 415. Lectotype ♀ design. by Morgan (1984: 9), Sweden: Uppsala (depository: LSL).
Chrysis (Tetrachrysis) fulgida : Trautmann 1927: 175 (E China, tax., descr.); Tsuneki 1950: 79 (comp. notes).
Chrysis (Chrysis) fulgida : Linsenmaier 1997b: 37 (key), 121 (colour table), 122 (Manchuria, tax., descr., *ignita* group, fig. 104).
Chrysis fulgida : Kimsey and Bohart 1991: 412 (cat).

######## Material examined.

Heilongjiang: 1♀, Harbin, 9.VII.1944 leg. Alin, identified by Linsenmaier in 1973 as *fulgida* ssp. *aequicolor* (NMLS).

######## Distribution.

China (Heilongjiang). Widely distributed from Europe to Asia (Dalla Torre 1892; Tsuneki 1953b; Kimsey and Bohart 1991; Kurzenko and Lelej 2007).

####### 112. Chrysis galloisi

Taxon classificationAnimaliaHymenopteraChrysididae

(du Buysson, 1908)

http://species-id.net/wiki/Tetrachrysis_galloisi

Chrysis galloisi *Tetrachrysis Galloisi* du Buysson, 1908: 210. Lectotype ♂ design. by Bohart (in Kimsey and Bohart 1991: 413), Japan (depository: MNHN)*.
Chrysis (Tetrachrysis) galloisi : Tsuneki 1948b: 52 (tax.).
Chrysis (Chrysis) galloisi : Linsenmaier 1959: 150 (key), 161 (tax., descr., *ignita* group).
Chrysis galloisi : Kimsey and Bohart 1991: 413 (lectotype design., cat., *ignita* group); Kurzenko and Lelej 2007: 1005 (China, cat.); Terayama et al. 2010: 4 (figs.), 8 (China, cat.), 12 (tab., biol.).

######## Distribution.

China. Southeastern Russia and Russian Far East, Japan (Tsuneki 1950; Kimsey and Bohart 1991; Kurzenko and Lelej 2007).

####### 113. Chrysis gracilenta

Taxon classificationAnimaliaHymenopteraChrysididae

Mocsáry, 1889

http://species-id.net/wiki/Chrysis_gracilenta

Plate 38

Chrysis (Tetrachrysis) gracilenta Mocsáry, 1889: 375. Holotype ♀, China: Hong Kong (375 (descr.), depository: NHMW)*.
Chrysis (Tetrachrysis) gracilenta : Bingham 1903: 437 (key), 464 (China, tax., descr.); Bischoff 1913: 52 (China, cat.).
Chrysis gracilenta : Dalla Torre 1892: 64 (China, cat.); Kimsey and Bohart 1991: 415 (Hong Kong, cat. *ignita* group).

######## Distribution.

China (Hong Kong). Burma, India (Bingham 1903, Bischoff 1913).

####### 114. Chrysis graelsii

Taxon classificationAnimaliaHymenopteraChrysididae

Guérin-Méneville, 1842

http://species-id.net/wiki/Chrysis_graelsii

Chrysis graelsii Guérin-Méneville, 1842: 148. Holotype ♀, Spain: Barcelona (148 (descr.), depository: MSNG)*.
Chrysis sybarita f. pekinensis Tsuneki, 1947: 57. Syntypes, China: Beijing (56 (tax., distr.), 57 (descr.), depository: NMLS)*.
Chrysis sybarita Förster, 1853: 309. Holotype ♂, Hungary ((309 (descr.), Type lost ?).
Chrysis (Chrysis) sybarita : Linsenmaier 1959: 135 (*sybarita* group).
Chrysis graelsii : Kimsey and Bohart 1991: 341 (fig. 111d), 347 (tax.), 415 (cat., *graelsii* group).

######## Material examined.

2♂♂, Peking N. China 20.V.38 K. Tsuneki / Types are much larger <handwritten by Tsuneki> / Paratype Chrysis sybarita var. pekinensis K. Tsuneki (NMLS).

######## Distribution.

China (Beijing). Europe, Iran, Kyrgyzstan, Siberia, Turkey (Linsenmaier 1959, 1968, 1997a; Semenov-Tian-Shanskij 1967; Tarbinsky 2002c).

######## Remarks.

Chrysis sybarita var. pekinensis Tsuneki is not mentioned in Kimsey and Bohart (1991).

####### 115. Chrysis grumorum

Taxon classificationAnimaliaHymenopteraChrysididae

Semenow, 1892

http://species-id.net/wiki/Chrysis_grumorum

Plate 39

Chrysis grumorum Semenow, 1892: 92. Holotype ♂ (not ♀), Tibet: Amdo (92 (descr.), depository: ZIN)*.
Chrysis (Tetrachrysis) grumorum : Bischoff 1913: 52 (Amdo, cat.).
Chrysis grumorum : Kimsey and Bohart 1991: 416 (Tibet: Amdo, cat., *ignita* group).

######## Distribution.

China (Tibet).

####### 116. Chrysis hoozana

Taxon classificationAnimaliaHymenopteraChrysididae

Mocsáry, 1913

http://species-id.net/wiki/Chrysis_hoozana

Plate 40

Chrysis hoozana Mocsáry, 1913b: 615. Holotype ♀ (not ♂), Taiwan: Hoozan [= Fengshan] (615 (descr.), 619 (Taiwan, cat.). depository: HNHM)*.
Chrysis (*Tetrachrysis) hoozana*: Uchida 1927: 151 (Taiwan, cat.); Uchida 1933: 5 (Taiwan, cat.).
Chrysis (Chrysis) hoozana : Tsuneki 1970b: 15 (Taiwan, tax., descr.).
Chrysis hoozana : Kimsey and Bohart 1991: 418 (Taiwan: Hoozan [= Fengshan], cat., *ignita* group).

######## Distribution.

China (Taiwan).

####### 117. Chrysis hyacinthus

Taxon classificationAnimaliaHymenopteraChrysididae

Semenov-Tian-Shanskij, 1967

http://species-id.net/wiki/Chrysis_hyacinthus

Plate 41

Chrysis (Tetrachrysis) hyacinthus Semenov-Tian-Shanskij, 1967: 168. Holotype ♀, China [Xinjiang]: Gashun, Bugas near Hami [Kumul] (168 (descr.), depository: ZIN)*.
Chrysis hyacinthus : Kimsey and Bohart 1991: 419 (North China, cat., *splendidula-senegalensis* group); Tarbinsky 2002c: 36 (sub *hiacithus* (!) key), 37 (China, cat.); Kurzenko and Lelej 2007: 1005 (China, cat.).

######## Distribution.

China (Xinjiang).

####### 118. Chrysis ignifascia

Taxon classificationAnimaliaHymenopteraChrysididae

Mocsáry, 1893

http://species-id.net/wiki/Chrysis_ignifascia

Chrysis (Holochrysis) birmanica Mocsáry, 1893: 214. Holotype ♂, Burma (214 (descr.), depository: MSNG)*.
Chrysis (Holochrysis) ignifascia Mocsáry, 1893: 215. Holotype ♀, Burma (215 (descr.), depository: MSNG)*.
Chrysis birmanica : Kimsey and Bohart 1991: 420 (synonym of *ignifascia*); Rosa 2009: 221 (tax., type, cat.).
Chrysis ignifascia : Kimsey and Bohart 1991: 420 (China, cat., *capitalis* group).
Chrysis (Holochrysis) ignifascia : Rosa 2009: 233 (tax., type, cat.).

######## Material examined.

1♀, Taiwan, Koshun, Apr. 1937, coll. K. Iwata (NMLS).

######## Distribution.

China (Taiwan). Burma (Mocsáry, 1893).

####### 119. Chrysis ignita

Taxon classificationAnimaliaHymenopteraChrysididae

(Linnaeus, 1758)

http://species-id.net/wiki/Sphex_ignita

Sphex ignita Linnaeus, 1758: 571. Lectotype ♀ design. by Richards (1935: 159), Europe (depository: LSL).
Chrysis (Tetrachrysis) ignita : Mocsáry 1889: 487 (tax.), 488 (China boreali, tax., descr.); Uchida 1927: 151 (North China, cat.); Tsuneki 1947: 55 (China: Beijing, cat.); Tsuneki 1948a: 126 (China: Hengshuichen-Henglingkuan, Henglingshan-Peihungkaokao, cat.), 128 (Beijing distr., Manchuria, Shanxi, cat.); Tsuneki 1953a: 58 (Manchuria: Wenchüansze; Heilongjiang: Harbin, tax.).
Chrysis ignita : Dalla Torre 1892: 69 (China boreali, cat.); du Buysson 1898a: 535 (“Montagnes au nord de Pèking”, cat.); du Buysson 1899: 167 (China, cat.); du Buysson 1900: 148 (China: Beijing, cat.); Kimsey and Bohart 1991: 317 (fig. 103), 336 (fig. 110a), 348 (tax., *ignita* group), 420 (cat.); Kurzenko and Lelej 2007: 1005 (China, cat.).
Chrysis ignita var.: Hammer 1936: 3 (China: Inner Mongolia: Hurtjertu Gol, cat.).
Chrysis (Chrysis) ignita : Linsenmaier 1959: 151 (key), 152 (key), 155 (tax., *ignita* group), 205 (fig. 388), 217 (fig. 696).

######## Distribution.

China (Heilongjiang, Inner Mongolia, Hebei, Beijing, Shanxi). Widely distributed in the Palaearctic Region (Trautmann 1927; Linsenmaier 1959; Kimsey and Bohart 1991).

######## Remarks.

Some identification may be related to other species belonging to the *ignita* group. The species is traditionally subdivided in two forms, *Chrysis ignita* A and B after Linsenmaier (1959), now recognized as two different species: *Chrysis terminata* Dahlbom and *Chrysis ignita* (Linnaeus).

####### 120. Chrysis illecebrosa

Taxon classificationAnimaliaHymenopteraChrysididae

Semenov-Tian-Shanskij, 1967

http://species-id.net/wiki/Chrysis_illecebrosa

Plate 42

Chrysis (Tetrachrysis) illecebrosa Semenov-Tian-Shanskij, 1967: 166. Holotype ♂, North China [= Xinjiang]: Bugas near Hami [Kumul] (166 (descr.), depository: ZIN)*.
Chrysis illecebrosa : Kimsey and Bohart 1991: 421 (North China, cat., *comparata* group).

######## Distribution.

China (Xinjiang).

####### 121. Chrysis inaequalis

Taxon classificationAnimaliaHymenopteraChrysididae

Dahlbom, 1845

http://species-id.net/wiki/Chrysis_inaequalis

Chrysis inaequalis Dahlbom, 1845: 8. Holotype ♀, Turkey: Bosfor (8 (descr.), type lost).
Chrysis (Tetrachrysis) inaequalis : Tsuneki 1947: 56 (China: Beijing, cat.); Tsuneki 1948a: 126 (China: Shanxi: Kiutauyüan, Hengshuichen, Hengshuichen-Henglingkuan, cat.), 128 (Beijing distr., Shanxi, cat.); Tsuneki 1953a: 59 (Manchuria: Kaiyüan, Lushan, cat., distr.).
Chrysis (Pentachrysis) inaequalis : Linsenmaier 1959: 165 (North China, Manchuria, tax., descr., *inaequalis* group), 205 (fig. 405), 213 (fig. 610); Linsenmaier 1997b: 38 (key), 126 (China, Manchuria, tax., descr., *inaequalis* group, fig. 110).
Chrysis inaequalis : Banaszak 1980: 28 (China, Manchuria, tax.); Kimsey and Bohart 1991: 329 (fig. 107q), 331 (108b), 335 (fig. 109k), 336 (fig 110g), 351 (tax.), 422 (cat.).

######## Material examined.

♀, Beijing, Russian mission, 5.IX.1906, leg. Y. Vasiliev (ZIN); ♀, Alashan, Din-yuan-in, 5.–6.VI.1908, leg. Kozlov (ZIN).

######## Distribution.

China (Liaoning, Inner Mongolia, Beijing, Shanxi). Widely distributed in central and southern Europe, Kyrgyzstan, Turkey (Linsenmaier 1959; Kimsey and Bohart 1991), Iran (Rosa et al. 2013), Kazakhstan, Tadjikistan, Transcaucasia, Uzbekistan (Tarbinsky 2002b), Siberia (Dalla Torre 1892), Russian Far East (Kurzenko and Lelej 2007).

####### 122. Chrysis ionophris

Taxon classificationAnimaliaHymenopteraChrysididae

Mocsáry, 1893

http://species-id.net/wiki/Chrysis_ionophris

Plate 43

Chrysis (Tetrachrysis) ionophris Mocsáry, 1893: 226. Holotype ♀, Burma (226 (descr.), depository: MSNG)*.
Chrysis ionophris : du Buysson 1899: 165 (China, cat.); Kimsey and Bohart 1991: 425 (Hong Kong, Taiwan, tax., *splendidula-senegalensis* group); Rosa 2009: 239 (tax., type, cat.).
Chrysis ionophris *Chrysis (Tetrachrysis) Schenklingi* Mocsáry, 1913b: 618. Lectotype ♀, design. by Bohart (in Kimsey and Bohart 1991: 425), Taiwan, depository: HNHM)* (synonymised by Kimsey and Bohart 1991: 425).
Chrysis (*Tetrachrysis) schenklingi*: Uchida 1927: 151 (Taiwan, cat.).
Chrysis (Chrysis) schenklingi : Tsuneki 1961: 375 (Taiwan, tax., descr., figs 22–24); Tsuneki 1970b: 14 (Taiwan: Manchou, Ssuchungchi, Hengchun, tax., descr.), 15 (figs 38–39).

######## Distribution.

China (Taiwan, Hong Kong). Burma, Laos, Sumatra, Thailand (Kimsey and Bohart 1991).

######## Remarks.

Tsuneki (1970b) added some morphological characteristics under the name *Chrysis schenklingi*.

####### 123. Chrysis jelisyni

Taxon classificationAnimaliaHymenopteraChrysididae

Radoszkowski, 1891

http://species-id.net/wiki/Chrysis_jelisyni

Chrysis jelisyni *Chrysis Jelisyni* Radoszkowski, 1891: 186. Holotype ♀, Mongolia [= China]: Kansu [= Gansu], Jelissyn-Kuce (186 (descr.), depository: ISEA-PAS)*.
Chrysis (Tetrachrysis) jelisyni : Bischoff 1913: 54 (Mongolia [= China], cat.).
Chrysis jelisyni : Kimsey and Bohart 1991: 426 (Mongolia [= China]: Kansu, cat., *comparata-scutellaris* group); Kurzenko and Lelej 2007: 1005 (China, cat.).

######## Distribution.

China (Gansu).

####### 124. Chrysis kashgarica

Taxon classificationAnimaliaHymenopteraChrysididae

Mocsáry, 1912

http://species-id.net/wiki/Chrysis_kashgarica

Chrysis kashgarica Mocsáry, 1912b: 550. Holotype ♂, China [Xinjiang]: Kashgar (550 (descr.), depository: HNHM)*.
Chrysis kashgarica : Kimsey and Bohart 1991: 427 (China: Sinkiang [= Xinjiang], cat., *ignita* group); Tarbinsky 2000: 194 (key), 198 (China, cat.).

######## Distribution.

China (Xinjiang).

####### 125. Chrysis keriensis

Taxon classificationAnimaliaHymenopteraChrysididae

Radoszkowski, 1887

http://species-id.net/wiki/Chrysis_keriensis

Chrysis keriensis Radoszkowski, 1887: 47. Holotype ♀, China [Xinjiang]: Keria-Daria (47 (descr.), depository: ISEA-PAS)*.
Chrysis (Tetrachrysis) keriensis : Mocsáry 1889: 516 (Mongolia [= China], tax., descr.); Bischoff 1913: 54 (Mongolia [= China], cat.).
Chrysis keriensis : Dalla Torre 1892: 73 (Mongolia [= China], cat.); Kimsey and Bohart 1991: 427 (Mongolia [= China], cat.); Kurzenko and Lelej 2007: 1005 (China: Xinjiang, cat.).

######## Distribution.

China (Xinjiang).

####### 126. Chrysis kokuevi

Taxon classificationAnimaliaHymenopteraChrysididae

Semenov-Tian-Shanskij, 1967

http://species-id.net/wiki/Chrysis_kukuevi

Plate 44

Chrysis (Tetrachrysis) kokuevi Semenov-Tian-Shanskij, 1967: 178. Holotype ♂, China: Alashan: Dyn-yuan-in oasis (178 (descr.), depository: ZIN)*.
Chrysis kokuevi : Kimsey and Bohart 1991: 428 (N China: Dyn-yuan-in oasis, cat.).

######## Distribution.

China (Inner Mongolia).

######## Remarks.

It belongs to the *succincta* group. The metasoma of the holotype was glued on a card beneth the specimen.

####### 127. Chrysis kozlovi

Taxon classificationAnimaliaHymenopteraChrysididae

Semenov-Tian-Shanskij, 1967

http://species-id.net/wiki/Chrysis_kozlovi

Plate 45

Chrysis (Gonodontochrysis) kozlovi Semenov-Tian-Shanskij, 1967: 160. Holotype ♂, North China: Alashan, Tzosto Canyon (160 (descr.), depository: ZIN)*.
Chrysis kozlovi : Kimsey and Bohart 1991: 429 (Mongolia [= Inner Mongolia], Tzosto Canyon, cat., *rufitarsis* group).

######## Distribution.

China (Inner Mongolia).

####### 128. Chrysis kukunorensis

Taxon classificationAnimaliaHymenopteraChrysididae

Semenov-Tian-Shanskij, 1967

http://species-id.net/wiki/Chrysis_kukunorensis

Plate 46

Chrysis (Tetrachrysis) kukunorensis Semenov-Tian-Shanskij, 1967: 178. Holotype ♀, China [Qinghai]: SE lake Kukunor [= Qinghai lake] (178 (descr.), depository: ZIN)*.
Chrysis kukunorensis : Kimsey and Bohart 1991: 429 (North China: SE Lake Kukunor, cat.).

######## Material examined.

1♀, China: Gansu Xiahe (Labhran) 3000–3500 m, 13–23.7.91, leg. P. Salk, Linsenmaier det. 1995 (NMLS).

######## Distribution.

China (Qinghai, Gansu).

######## Remarks.

It belongs to the *ignita* group.

####### 129. Chrysis lama

Taxon classificationAnimaliaHymenopteraChrysididae

Mocsáry, 1914

http://species-id.net/wiki/Chrysis_lama

Plate 47

Chrysis lama Mocsáry, 1914: 45. Lectotype ♂ design. by Bohart (in Kimsey and Bohart 1991: 431), Tibet: Gyantse (depository: BMNH)*.
Chrysis lama : Kimsey and Bohart 1991: 431 (Tibet: Gyantse, lectotype design., cat., *ignita* group).

######## Distribution.

China (Tibet).

####### 130. Chrysis lincea

Taxon classificationAnimaliaHymenopteraChrysididae

Fabricius, 1775

http://species-id.net/wiki/Chrysis_lincea

Chrysis lincea Fabricius, 1775: 367. Holotype, Sierra Leone (367 (descr.), depository: Drury coll.).
Chrysis lyncea (!): du Buysson 1898b: 560 (China, cat.).
Chrysis (Pyria) lyncea (!): Linsenmaier 1959: 178 (*lyncea* group).
Chrysis lincea : Kimsey and Bohart 1991: 326 (fig. 106u), 331 (figs 108o, 108p), 352 (cat.), 357 (fig. 113f), 433 (*lincea* group, cat.).

######## Distribution.

China. Widely distributed in Asia, Australia and the Afrotropical Region (Linsenmaier 1959; Kimsey and Bohart 1991; Madl and Rosa 2012).

####### 131. Chrysis longiss ima

Taxon classificationAnimaliaHymenopteraChrysididae

du Buysson, 1898

http://species-id.net/wiki/Chrysis_longissima

Chrysis longissima du Buysson, 1898b: 529. Lectotype ♀ design. by Bohart (in Kimsey and Bohart 1991: 433), China: Kiang-si [= Jiangxi] (depository: MNHN).
Chrysis longissima : du Buysson 1899: 165 (China, cat.); Kimsey and Bohart 1991: 433 (China: Kiang-si [= Jiangxi], cat.); Kurzenko and Lelej 2007: 1005 (China, cat.).
Chrysis (Tetrachrysis) longissima : Bischoff 1913: 55 (China: Kiang-si [= Jiangxi], cat.).

######## Distribution.

China (Jiangxi).

####### 132. Chrysis longula aeneopaca

Taxon classificationAnimaliaHymenopteraChrysididae

Linsenmaier, 1959

http://species-id.net/wiki/Chrysis_longula_aeneopaca

Chrysis (Chrysis) longula ssp. aeneopaca Linsenmaier, 1959: 160. Holotype ♀, Transcaspia (160 (type series: China, Fennoscandia, Japan, Siberia, Transcaspia, descr.), *ignita* group, depository: NMLS)*.
Chrysis longula : Kimsey and Bohart 1991: 433 (cat.).

######## Distribution.

China. Fennoscandia, Japan, Siberia, Transcaspia (Linsenmaier 1959).

####### 133. Chrysis mane

Taxon classificationAnimaliaHymenopteraChrysididae

Semenov-Tian-Shanskij, 1912

http://species-id.net/wiki/Chrysis_mane

Plate 48

Chrysis mane Semenov-Tian-Shanskij, 1912: 192. Lectotype ♂, China: Alashan (192 (descr.), depository: ZIN)*.
Chrysis mane : Kimsey and Bohart 1991: 436 (China [not Mongolia]: Gansu, Quingai, cat., *ignita* group).

######## Material examined.

33♂♂, Din-yuan-in, IV.-VI.1908–1909, leg. Kozlov (ZIN); 1♂, env. Lang Zhou, 24.IV.1909, leg. Kozlov (ZIN); 4♀♀, env. Sinin, 1.–6.IX.1908, leg. Kozlov (ZIN); 19♀♀, Din-yuan-in, Alashan, IX.1908, leg. Kozlov (ZIN); 1♀, Ning-sia-fu, Yellow river Valley, 1.–4.VI.1908, leg. Kozlov (ZIN); 1♀, between Tsing-yung-siang and Pilung-gu-ang, 1908, leg. Kozlov (ZIN); 3♀♀, Alashan, Tzosto Canyon, 20.–26.V.1908, leg. Kozlov (ZIN).

######## Distribution.

China (Gansu, Qinghai, Inner Mongolia).

######## Remarks.

The lectotype bears the locality label: Alashan, oasis Din-yuan-in, 23.–26.IV.1909, expedition Kozlov.

####### 134. Chrysis maracandensis

Taxon classificationAnimaliaHymenopteraChrysididae

Radoszkowski, 1877

http://species-id.net/wiki/Chrysis_maracandensis

Chrysis maracandensis Radoszkowski, 1877: 14. Lectotype ♂ [not ♀] design. by Bohart (in Kimsey and Bohart 1991: 436), Uzbekistan: Sarafschan (depository: MMU)*.
Chrysis (Tetrachrysis) maracandensis : Bischoff 1910: 473 (Chinese Turkestan [= Xinjiang]: Pjalma-Chotan, cat.).
Chrysis maracandensis : Kimsey and Bohart 1991: 436 (cat., *comparata-scutellaris* group).

######## Distribution.

China (Xinjiang). Turkmenistan, Uzbekistan (Kimsey and Bohart 1991; Tarbinsky 2002c).

####### 135. Chrysis marginata

Taxon classificationAnimaliaHymenopteraChrysididae

Mocsáry, 1889

http://species-id.net/wiki/Chrysis_marginata

Chrysis marginata Mocsáry 1889: 451. Holotype ♀; Turkestan (451 (descr.), depository: ISEA-PAS)*.
Chrysis (Tetrachrysis) marginata : Bischoff 1910: 479 (Chinese Turkestan [= Xinjiang]: Pjalma-Chotan, cat.); Semenov-Tian-Shanskij and Nikol’skaja 1954: 127 (China: Xinjiang, tax.).
Chrysis (Chrysis) marginata : Linsenmaier 1959: 146 (key, tax., descr., *comparata* group), 204 (fig. 373).
Chrysis marginata : Kimsey and Bohart 1991: 436 (cat., *comparata* group); Tarbinsky 2002c: 34 (China: Xinjiang, key, cat., distr.), 42 (figs 18–19).

######## Distribution.

China (Xinjiang). Southeastern Europe, Cyprus, Greece, Iran, Kazakhstan, Palestine, Tadjikistan, Turkey, Turkmenistan, Uzbekistan (Linsenmaier 1959; Tarbinsky 2002c; Rosa et al. 2013).

####### 136. Chrysis matutina

Taxon classificationAnimaliaHymenopteraChrysididae

Semenov-Tian-Shanskij, 1967

http://species-id.net/wiki/Chrysis_matutina

Plate 49

Chrysis (Tetrachrysis) matutina Semenov-Tian-Shanskij, 1967: 179. Holotype ♀, China: Gansu (179 (descr.), depository: ZIN)*.
Chrysis matutina : Kimsey and Bohart 1991: 437 (China: Hansiu, cat., *ignita* group); Kurzenko and Lelej 2007: 1005 (China: Hubei, cat., *ignita* group).

######## Distribution.

China (Gansu, Hubei).

####### 137. Chrysis mongoliana

Taxon classificationAnimaliaHymenopteraChrysididae

Bohart, 1991

http://species-id.net/wiki/Chrysis_mongoliana

Plate 50

Chrysis (Tetrachrysis) mongolica Semenov-Tian-Shanskij, 1967: 179. Holotype ♀, Mongolia: Transbaikal, Ingoda river (type series: N China: Alashan; 179 (descr.), depository: ZIN)* *nec* Mocsáry, 1914.
Chrysis mongoliana Bohart (in Kimsey and Bohart), 1991: 440. Replacement name for Chrysis (Tetrachrysis) mongolica Semenov-Tian-Shanskij, 1967 *nec* Mocsáry, 1914.
Chrysis mongoliana : Kurzenko and Lelej 2007: 1005 (China: Gansu, cat.).

######## Material examined.

1♀, Alashan, Din-yuan-in, 20.V.1908, leg. Kozlov (ZIN); 1♀, Alashan, Tzosto Canyon, 25.V.1908, leg. Kozlov (ZIN):

######## Distribution.

China (Inner Mongolia, Gansu).

####### 138. Chrysis nigricincta

Taxon classificationAnimaliaHymenopteraChrysididae

Bischoff, 1910

http://species-id.net/wiki/Chrysis_nigricincta

Chrysis (Tetrachrysis) nigricincta Bischoff, 1910: 475. Holotype ♂, Chinese Turkestan [= Xinjiang]: Pjalma-Chotan (475 (descr.), depository: MNHU)*.
Chrysis (Tetrachrysis) nigricincta : Bischoff 1913: 56 (Chinese Turkestan [= Xinjiang], cat.).
Chrysis nigricincta : Kimsey and Bohart 1991: 443 (China: Sinkiang [= Xinjiang], cat.).

######## Distribution.

China (Xinjiang).

####### 139. Chrysis nigropilosa

Taxon classificationAnimaliaHymenopteraChrysididae

Tsuneki, 1970

http://species-id.net/wiki/Chrysis_nigropilosa

Chrysis (Tetrachrysis) nigropilosa Tsuneki, 1970b: 16. Holotype ♀, Taiwan: Chiai Province, Fenchifu, 1400 m (16 (descr.), 17 (type series: Taiwan: Fenchifu, Chienching, descr., figs 42–48), depository: OMNH, not NIAS).
Chrysis nigropilosa : Kimsey and Bohart 1991: 443 (Taiwan: Chiai, Fenchifu, cat.).

######## Distribution.

China (Taiwan).

####### 140. Chrysis parallela

Taxon classificationAnimaliaHymenopteraChrysididae

Brullé, 1846

http://species-id.net/wiki/Chrysis_parallela

Plate 51

Chrysis parallela Brullé, 1846: 29. Holotype ♀ [not ♂], Indonesia: Timor Is. (29 (descr.), depository: MNHN).
Chrysis parallela : du Buysson 1901b: 104 (China, cat.); Kimsey and Bohart 1991: 447 (cat., *smaragdula* group).
Chrysis fukaii Rohwer, 1911: 478. Holotype ♀, Taiwan: Horisha (478 (descr.), depository: USNM) (synonymised by Kimsey and Bohart 1991).
Chrysis assamensis Mocsáry, 1913a: 24. Holotype ♀, India: Assam (24 (descr.), depository: HNHM)* (synonymised by Kimsey and Bohart 1991).
Chrysis principalis var. assamensis : Tsuneki 1963a: 4 (Taiwan, tax.).
Chrysis fukaii : Tsuneki 1963a: 4 (possible syn. of *principalis* Smith, 1874); Tsuneki 1970b: 19 (synonym of *principalis* Smith, 1874).

######## Distribution.

China (Taiwan). Widely distributed in the Oriental Region (Kimsey and Bohart 1991).

######## Rermarks.

*Chrysis fukaii* Rohwer was previously synonymised with *Chrysis principalis* Smith, 1874 by Tsuneki (1970), who also considered *Chrysis assamensis* as a colour variation of *Chrysis principalis* (Tsuneki 1963a).

####### 141. Chrysis pleskei

Taxon classificationAnimaliaHymenopteraChrysididae

Semenow, 1892

http://species-id.net/wiki/Chrysis_pleskei

Plate 52

Chrysis pleskei *Chrysis Pleskei* Semenow, 1892a: 257. Lectotype ♀ design. by Bohart (in Kimsey and Bohart 1991: 449), China [Xinjiang]: Sandzhu (depository: ZIN)*.
Chrysis (Tetrachrysis) pleskei : Bischoff 1910: 483 (Chinese Turkestan [= Xinjiang]: Pjalma-Chotan, cat.); Bischoff 1913: 57 (Chinese Turkestan [= Xinjiang]: Pjalma-Chotan, cat.).
Chrysis pleskei : Kimsey and Bohart 1991: 449 (China: Sinkiang [= Xinjiang], cat., *comparata-scutellaris* group); Kurzenko and Lelej 2007: 1005 (China: Xinjiang: Sandzhu, cat.).

######## Distribution.

China (Xinjiang).

####### 142. Chrysis potanini

Taxon classificationAnimaliaHymenopteraChrysididae

Radoszkowki, 1891

http://species-id.net/wiki/Chrysis_potanini

Chrysis potanini Radoszkowski, 1891: 186. Holotype ♂, Mongolia [= China]: Tufyn (ISEA-PAS)*.
Chrysis (Tetrachrysis) potanini : Bischoff 1911: 57 (Mongolia [= China], cat.).
Chrysis potanini : Kimsey and Bohart 1991: 450 (Mongolia [= China], cat.).

######## Distribution.

China.

######## Remarks.

It belongs to the *comparata* group.

####### 143. Chrysis principalis

Taxon classificationAnimaliaHymenopteraChrysididae

Smith, 1874

http://species-id.net/wiki/Chrysis_principalis

Chrysis principalis Smith, 1874b: 461. Holotype ♀, China: Shanghai (46 (descr.), depostiory: HEC).
Chrysis (Hexachrysis) principalis : Mocsáry 1889: 559 (China, descr., distr.); Bischoff 1910: 490 (Taiwan, cat.); Mocsáry 1913b: 619 (Taiwan: Takao [= Kaohsiung], Taihorinsho, Anping, Kosempo, cat.); Uchida 1927: 152 (Taiwan, North China, cat.); Uchida 1933: 4 (Taiwan, North China, cat.); Tsuneki 1948a: 127 (China: Shanxi: Yangchêng, cat., distr.), 128 (Shanxi, cat.); Tsuneki 1953b: 28 (East coast of China, Taiwan, tax.); Tsuneki 1961: 377 (North China, Taiwan, tax.); Tsuneki 1963a: 2 (East China, Taiwan, key, tax., descr., distr.), 3 (figs 1–8), 4 (China as far north as Manchuria (Dairen), Taiwan: Taihoku, Heito, Kuraru, distr., comp. notes), 9 (comp. notes, fig. 9).
Chrysis principalis : Dalla Torre 1892: 85 (China, cat.); du Buysson 1898a: 537 (China: Kiang-si [= Jiangxi], cat.), 558 (North China: Pena, cat.); du Buysson 1899: 168 (China, cat.); du Buysson 1900: 153 (China: Beijing, cat.); Bingham 1903: 440 (key), 490 (tax., descr.), 491 (China, distr.); Kimsey and Bohart 1991: 450 (China: Shanghai, cat., *smaragdula* group); Kurzenko and Lelej 2007: 1005 (China, cat.); Terayama et al. 2010: 8 (China, Taiwan, cat.).
Hexachrysis principalis : Hammer 1950: 2 (China: Kiangsu [= Jiangsu], cat.).
Chrysis (Pyria) principalis : Tsuneki 1970b: 18 (Taiwan, cat.).

######## Material examined.

1♀, Taiwan, Chipon, VIII.1935, leg. K. Iwata, det. Linsenmaier 1974 (NMLS).

######## Distribution.

China (Liaoning, Beijing, Shanxi, Jiangsu, Shanghai, Jiangxi, Taiwan). India, Sri Lanka, Sumatra, Java, Celebes, Siam, Aru Is., Korea (Tsuneki 1953b).

######## Remarks.

Tsuneki (1970b) considered *Chrysis buddhae* Mocsáry, 1913 and *Chrysis fukai* Rowhwer, 1911 junior synonyms of *Chrysis principalis* Smith, 1874. Kimsey and Bohart (1991) considered *Chrysis buddhae* Mocsáry, 1913 as a valid species and *Chrysis fukai* as a synonym of *Chrysis parallela* Brullé, 1846.

####### 144. Chrysis przewalskii

Taxon classificationAnimaliaHymenopteraChrysididae

Radoszkovski, 1887

http://species-id.net/wiki/Chrysis_przewalskii

Chrysis przewalskii *Chrysis Przewalskii* Radoszkowski, 1887: 46. Holotype ♂, Mongolia [= China]: Zaïdam (46 (descr.), depository: ISEA-PAS)*.
Chrysis (Tetrachrysis) przewalskii : Mocsáry 1889: 504 (Mongolia [= China], tax., descr.); Bischoff 1913: 57 (Mongolia [= China], cat.).
Chrysis przewalskii : Dalla Torre 1892: 86 (Mongolia [= China], cat.); Kimsey and Bohart 1991: 452 (Mongolia [= China], Keria Mts., cat., *pulchella* group).

######## Distribution.

China (Xinjiang, Qinghai).

####### 145. Chrysis rutilans extranea

Taxon classificationAnimaliaHymenopteraChrysididae

Linsenmaier, 1959

http://species-id.net/wiki/Chrysis_rutilans_extranea

Chrysis (Chrysis) rutilans ssp. extranea : Linsenmaier 1959: 128. Holotype ♂, Japan (128 (key, tax., descr., *splendidula* group), 203 (fig. 302), 211 (figs 566, 569); depository: NMLS)*.
Chrysis rutilans : Radoszkovsky 1866: 12 (China, tax.); Kimsey and Bohart 1991: 458 (cat., *splendidula* group).
Chrysis (Chrysis) rutilans ssp. extranea : Linsenmaier 1997b: 101 (China [Heilongjiang], tax.).

######## Material examined.

Heilongjiang: 1♂, Harbin, 18.VI.1944 [Paratype]; 1♂ labeled: [Szechuen, China, Kintung, Chauchiatu 24.IV.49 Y.W. Djon] [Paratype] (NMLS).

######## Distribution.

China (Heilongjiang, Sichuan). Europe and Eurasia (Linsenmaier 1959, Kimsey and Bohart 1991).

######## Remarks.

The specimens identified as *Chrysis rutilans* Olivier by Radoszkovsky (1866) is very likely referrable to other species, based on wrong identifications found in his collection.

####### 146. Chrysis rutiliventris nankingensis

Taxon classificationAnimaliaHymenopteraChrysididae

Linsenmaier, 1959

http://species-id.net/wiki/Chrysis_rutiliventris_nankingensis

Chrysis (Chrysis) rutiliventris ssp. nankingensis Linsenmaier, 1959: 153. Holotype ♀, China [Jiangsu]: Nanking [=Nanjing] (153 (descr.), *ignita* group, depository: NMLS)*.
Chrysis rutiliventris : Kimsey and Bohart 1991: 458 (China: Nan King [= Nanjing], cat., *ignita* group); Kurzenko and Lelej 2007: 1005 (China, cat.).

######## Material examined.

Jiangsu: 1♀, Nanking China 30.IV.23 / Presented by Van Dyke collector / Type ♀ *Chrysis* L. *rutiliventris* ssp. *nankingensis* det. Linsenmaier 1959 (NMLS).

######## Distribution.

China (Jiangsu). Europe, Eurasia and North Africa (Kimsey and Bohart 1991).

####### 147. Chrysis schalfeewi

Taxon classificationAnimaliaHymenopteraChrysididae

Semenow, 1892

http://species-id.net/wiki/Chrysis_schalfeewi

Plate 53

Chrysis schalfeewi *Chrysis Schalfeewi* Semenow, 1892: 80. Holotype ♂, China [Xinjiang]: Sandzhu (80 (descr.), depository: ZIN)*.
Chrysis (Tetrachrysis) schalfewi (!): Bischoff 1913: 58 (China, cat.). Incorrect emendation.
Chrysis schalfeewi : Kimsey and Bohart 1991: 459 (China: Sinkiang [= Xinjiang]: cat., *comparata-scutellaris* group); Kurzenko and Lelej 2007: 1005 (China: Xinjiang, cat.).

######## Distribution.

China (Xinjiang).

####### 148. Chrysis serena

Taxon classificationAnimaliaHymenopteraChrysididae

Radoszkowski, 1891

http://species-id.net/wiki/Chrysis_serena

Chrysis serena Radoszkowski, 1891: 194. Holotype ♂, Iran: Sarakhs (194 (descr.), depository: ISEA-PAS)*.
Chrysis (Chrysis) pyrrhina ssp. serena : Linsenmaier 1968: 82 (Manchuria [= Heilongjiang], tax., descr., distr., *viridula* group).
Chrysis pyrrhina Dahlbom, 1854: Kimsey and Bohart 1991: 454 (cat.).

######## Material examined.

Heilongjiang: 1♀, Harbin, 19.VII.1953 (NMLS).

######## Distribution.

China (Heilongjiang).

####### 149. Chrysis sinensis

Taxon classificationAnimaliaHymenopteraChrysididae

du Buysson, 1898

http://species-id.net/wiki/Chrysis_ignita_sinensis

Chrysis ignita var. sinensis du Buysson, 1898b: 139. Holotype ♀, China: Shanghai (139 (descr.), depository: MNHN).
Chrysis (Tetrachrysis) ignita var. sinensis : Bischoff 1913: 53 (China, cat.); Trautmann 1927: 145 (cat.), 147 (Shanghai, tax.).
Chrysis (Chrysis) sinensis : Linsenmaier 1959: 152 (key), 155 (China, tax., descr., *ignita* group).
Chrysis sinensis : Kimsey and Bohart 1991: 463 (cat., *ignita* group); Kurzenko and Lelej 2007: 1005 (China, cat.).

######## Distribution.

China (Shanghai). Japan (Kimsey and Bohart 1991).

####### 150. Chrysis spinidens

Taxon classificationAnimaliaHymenopteraChrysididae

Mocsáry, 1887

http://species-id.net/wiki/Chrysis_spinidens

Chrysis (Tetrachrysis) spinidens Mocsáry (inédite) in Radoszkowski, 1887: 48. Holotype ♂, Mongolia [= China]: Zaïdam (48 (descr.), depository: ISEA-PAS)*.
Chrysis spinidens : Dalla Torre 1892: 97 (Mongolia [= China], cat.); Kimsey and Bohart 1991: 464 (Mongolia [= China], cat.).
Chrysis (Tetrachrysis) spinidens : Bischoff 1913: 59 (Mongolia [= China], cat.).

######## Distribution.

China (Qinghai).

######## Remarks.

It belongs to the *ignita* group.

####### 151. Chrysis splendidula

Taxon classificationAnimaliaHymenopteraChrysididae

Rossi, 1790

http://species-id.net/wiki/Chrysis_splendidula

Chrysis splendidula Rossi, 1790: 76. Syntypes, Italy (76 (descr.), depository: MNHU)*.
Chrysis (Tetrachrysis) splendidula : Tsuneki 1953a: 59 (Manchuria: Ronshui near Heiho, tax., distr.); Tsuneki 1953b: 26 (Manchuria, cat., distr.).
Chrysis (Chrysis) splendidula : Linsenmaier 1959: 127 (tax., descr., distr., *splendidula* group), 203 (fig. 301).
Chrysis splendidula : Kimsey and Bohart 1991: 341 (fig. 111g), 465 (cat., *splendidula* gr.).

######## Distribution.

China (Heilongjiang). Widely distributed in the Palaearctic Region from south Europe and North Africa to Central Asia, Korea and Japan (Kimsey and Bohart 1991).

####### 152. Chrysis strauchi

Taxon classificationAnimaliaHymenopteraChrysididae

Semenow, 1892

http://species-id.net/wiki/Chrysis_strauchi

Plate 54

Chrysis strauchi *Chrysis Strauchi* Semenow, 1892a: 85. Holotype ♂, Chinese Turkestan [= Xinjiang]: oasis Sandzhu (85 (descr.), depository: ZIN)*.
Chrysis (Tetrachrysis) stranchi (!): Bischoff 1910: 483 (Chinese Turkestan [= Xinjiang], cat.).
Chrysis (Tetrachrysis) strauchi : Bischoff 1913: 60 (Chinese Turkestan [= Xinjiang], cat.).
Chrysis strauchi : Kimsey and Bohart 1991: 466 (China [Xinjiang], cat., *comparata-scutellaris* group); Kurzenko and Lelej 2007: 1005 (China: Xinjiang, cat.).

######## Distribution.

China (Xinjiang).

####### 153. Chrysis taihorina

Taxon classificationAnimaliaHymenopteraChrysididae

Mocsáry, 1913

http://species-id.net/wiki/Chrysis_taihorina

Plate 55

Chrysis (Tetrachrysis) taihorina Mocsáry, 1913b: 617. Holotype ♂, Taiwan: Taihorin (617 (descr.), 619 (Taiwan, cat.), depository: HNHM)*.
Chrysis (*Tetrachrysis) taihorina*: Uchida 1927: 151 (Taiwan, cat.); Uchida 1933: 5 (Taiwan, cat.).
Chrysis (Chrysis) taihorina : Tsuneki 1970b: 16 (Taiwan, cat.).
Chrysis taihorina : Kimsey and Bohart 1991: 469 (Taiwan, cat., *ignita* group).

######## Distribution.

China (Taiwan).

####### 154. Chrysis taiwana

Taxon classificationAnimaliaHymenopteraChrysididae

Tsuneki, 1970

http://species-id.net/wiki/Chrysis_taiwana

Chrysis (Chrysura) taiwana Tsuneki, 1970b: 7. Holotype ♂, Taiwan [Pintung Province]: Hengchun (7 (tax., descr., figs 9–13), 8 (Taiwan, Hengchun), depository: OMNH).
Chrysis taiwana : Kimsey and Bohart 1991: 470 (Taiwan, cat., *capitalis* group).

######## Distribution.

China (Taiwan).

####### 155. Chrysis takasago

Taxon classificationAnimaliaHymenopteraChrysididae

Tsuneki, 1963

http://species-id.net/wiki/Chrysis_takasago

Chrysis (Hexachrysis) takasago Tsuneki, 1963a: 4. Syntypes ♀♀, Taiwan: Gyochi, Taihoku, Shinka, Kuraru, Urai (1 (Taiwan, key), 4 (tax., descr.), 5 (comp. notes, figs 10–12), depository: OMNH).
Chrysis (Pyria) principalis ssp. takasago : Tsuneki 1970b: 19 (Taiwan: Ilan, Hualien, Nantou, Taitung, Pingtung, tax., aberr.).
Chrysis takasago : Kimsey and Bohart 1991: 470 (Taiwan, cat., *smaragdula* group).

######## Distribution.

China (Taiwan).

####### 156. Chrysis takeuchii

Taxon classificationAnimaliaHymenopteraChrysididae

Tsuneki, 1950

http://species-id.net/wiki/Chrysis_takeuchii

Chrysis (Tetrachrysis) takeuchii Tsuneki, 1950: 76. Holotype ♀, Taiwan: Horisha (76 (descr.), 77 (type series: Taiwan: Horisha, Takesaki), depository: OMNH).
Chrysis (Chrysis) takeuchii : Tsuneki 1970b: 14 (Taiwan: Nanshanchi, tax., descr.), 15 (figs 40–41).
Chrysis takeuchii : Kimsey and Bohart 1991: 470 (Taiwan: Horisha, cat.).

######## Distribution.

China (Taiwan).

####### 157. Chrysis talitha

Taxon classificationAnimaliaHymenopteraChrysididae

Mocsáry, 1913

http://species-id.net/wiki/Chrysis_talitha

Plate 56

Chrysis talitha *Chrysis (Tetrachrysis) Talitha* Mocsáry, 1913b: 616. Holotype ♀, Taiwan: Taihorinsho (616 (descr.), 619 (Taiwan, cat.), depository: HNHM)*.
Chrysis (*Tetrachrysis) talitha*: Uchida 1927: 152 (Taiwan, cat.); Uchida 1933: 5 (Taiwan, cat.).
Chrysis (Chrysis) talitha : Tsuneki 1970b: 15 (Taiwan: Taihorinsho, cat.).
Chrysis talitha : Kimsey and Bohart 1991: 470 (Taiwan: Taihorinsho, cat., *splendidula-senegalensis* group).

######## Distribution.

China (Taiwan).

####### 158. Chrysis tibetana

Taxon classificationAnimaliaHymenopteraChrysididae

Mocsáry, 1914

http://species-id.net/wiki/Chrysis_tibetana

Plate 57

Chrysis (Tetrachrysis) tibetana Mocsáry, 1914: 43. Lectotype ♂ design. by Bohart (in Kimsey and Bohart 1991: 471), Tibet: Gyangtse (depository: BMNH)*.
Chrysis tibetana : Kimsey and Bohart 1991: 471 (Tibet: Gyangtse, lectotype design., cat., *ignita* group).

######## Material examined.

Tibet: Paralectotypes, 1♂, Gyangtse. 13,000ft June 1904 Tibet Exp. H.J. Walton 1905–173; 1♀, Tibet: Kyishong. 14,500 ft 10.VII.1924 Maj. R.W.G.Hingston / Everest Exp. Brit. Mus. 1924–386; both specimens identified by Linsenmaier in 1966 (NMLS).

######## Distribution.

China (Tibet).

####### 159. Chrysis tsingtauensis

Taxon classificationAnimaliaHymenopteraChrysididae

Bischoff, 1910

http://species-id.net/wiki/Chrysis_tsingtauensis

Chrysis (Tetrachrysis) tsingtauensis Bischoff, 1910: 482. Lectotype ♂ design. by Bohart (in Kimsey and Bohart 1991: 471), China [Qingdao]: Kiautschau [= Jiaozhou Bay], Tsingtau (MNHU)*.
Chrysis (Tetrachrysis) tsingtauensis : Bischoff 1913: 60 (Tsingtau, cat.).
Chrysis tsingtauensis : Kimsey and Bohart 1991: 471 (China [Qingdao]: Kiautschau, Tsingtau, lectotype design., cat., *ignita* group).

######## Material examined.

1♂, Shanghai, 15.V.1923; 1♀, China “C.F.”, Lju Coll.; both specimens identified by Linsenmaier 1966, but to be considered as doubtful identifications.

######## Distribution.

China (Shandong, Shanghai).

####### 160. Chrysis varicolor

Taxon classificationAnimaliaHymenopteraChrysididae

Smith, 1874

http://species-id.net/wiki/Chrysis_varicolor

Chrysis varicolor Smith, 1874b: 482. Holotype ♂, China [Fujian]: Foochow [= Fuzhou] (482 (descr.), depository: BMNH).
Chrysis (Tetrachrysis) variicolor (!): Mocsáry 1889: 539 (China: Ta-tschian-sy, tax., descr.).
Chrysis variicolor (!): Dalla Torre 1892: 105 (China borealis, cat.).
Chrysis (Hexachrysis) variicolor (!): Bischoff 1913: 68 (North China, cat.).
Chrysis varicolor : Kimsey and Bohart 1991: 474 (cat., *ignita* group); Kurzenko and Lelej 2007: 1005 (China: Fujian, cat.).

######## Distribution.

China (Fujian).

####### 161. Chrysis vicaria

Taxon classificationAnimaliaHymenopteraChrysididae

Mocsáry, 1913

http://species-id.net/wiki/Chrysis_vicaria

Plate 58

Chrysis (Hexachrysis) vicaria Mocsáry, 1913a: 11. Lectotype ♂ design. by Bohart (in Bohart and French 1986: 343), China: Taiwan: Taihorinsho (11 (descr.), 12 (type series: Takao, Fuhosho, Tainan, Taihorinsho), depository: HNHM)*.
Chrysis (Hexachrysis) vicaria : Mocsáry 1913b: 619 (Taiwan: Anping, Takao, Taihorinsho, Tainan, Fuhosho, cat.); Bischoff 1913: 68 (Taiwan, cat.); Uchida 1927: 152 (Taiwan, cat.).
Chrysis (Hexachrysis) fasciata vicaria : Tsuneki 1962: 2 (Taiwan, cat.); Tsuneki 1963a: 1 (Taiwan and probably eastern coast of China, key), 6 (tax., descr.), 7 (Taiwan, comp. notes, figs 13–15), 9 (comp. notes).
Chrysis (Pyria) fasciata vicaria : Tsuneki 1970b: 19 (Taiwan: Ilan, Nantou, Pingtung, cat.); Tsuneki 1970c; 49 (Taiwan, key, tax.).
Chrysis vicaria : Kimsey and Bohart 1991: 476 (Taiwan: Taihorinsho, cat., *smaragdula* group); Terayama et al. 2010: 9 (China (?), Taiwan, cat.).

######## Material examined.

1♀, Taiwan; Kosempo 7–19.IV.1908 leg. H. Sauter, identified by Linsenmaier 1973 (NMLS).

######## Distribution.

China (Taiwan).

######## Remarks.

Tsuneki (1962, 1963a, 1970b) considered *Chrysis vicaria* as a subspecies of *Chrysis fasciata* Olivier, 1790.

####### 162. Chrysis violenta ultramonticola

Taxon classificationAnimaliaHymenopteraChrysididae

Linsenmaier 1968

http://species-id.net/wiki/Chrysis_violenta_ultramonticola

Chrysis (Chrysis) violenta ssp. ultramonticola Linsenmaier, 1968: 97. Holotype ♀, Tibet: Tropde, 11.000 ft (97 (Tibet: Tropde, 11.000 ft; Rongshar, 13.000 ft, descr.), *ignita* group, depository: BMNH).
Chrysis violenta : Kimsey and Bohart 1991: 477 (Tibet, Everest Region: Tropde, cat., *ignita* group).

######## Distribution.

China (Tibet). Nepal (Boesi et al. 2005).

######## Host.

Boesi et al. (2005) found three specimens of *Chrysis violenta ultramonticola* in the nest of *Ancistrocerus sikhimensis* Bingham (Hymenoptera, Vespidae, Eumeninae).

####### 163. Chrysis viridula

Taxon classificationAnimaliaHymenopteraChrysididae

Linnaeus, 1761

http://species-id.net/wiki/Chrysis_viridula

Chrysis viridula Linnaeus, 1761: 415. Holotype ♂; Europe (415 (descr.), depository: LSL).
Chrysis (Tetrachrysis) viridula f. apicata Uchida, 1927: Tsuneki 1948b: 47 (Manchuria, tax.).
Chrysis (Tetrachrysis) viridula : Tsuneki 1953a: 59 (Manchuria [Jilin]: Chintsang, North of Kunchun, tax., distr.).
Chrysis (Chrysis) viridula : Linsenmaier 1959: 129 (key, *viridula* group), 130 (tax., descr.), 203 (fig. 304), 211 (figs 561, 562).
Chrysis viridula : Kimsey and Bohart 1991: 329 (fig. 107a), 341 (fig.111h), 367 (cit.), 329 (fig. 197a), 341 (fig. 111h), 477 (cat.).

######## Distribution.

China (Jilin). Widely distributed in the Palaearctic to Russian Far East (Kimsey and Bohart 1991; Kurzenko and Lelej 2007).

######## Remarks.

In Kimsey and Bohart (1991) Chrysis viridula var. apicata Uchida, 1927 from Japan is included in the synonymic list for *viridula*, but according to Tsuneki (1948b, 1953a), it is a synonym of *splendidula* Rossi, 1790.

####### 164. Chrysis volatilis

Taxon classificationAnimaliaHymenopteraChrysididae

Smith, 1874

http://species-id.net/wiki/Chrysis_volatilis

Chrysis volatilis Smith, 1874b: 459. Holotype ♀, Shanghai (459 (descr.), depository: BMNH).
Chrysis (Tetrachrysis) volatilis : Mocsáry 1889: 374 (Shanghai, descr., cat.); Bischoff 1913: 61 (North China, cat.).
Chrysis volatilis : Dalla Torre 1892: 109 (Shanghai, cat.); Kimsey and Bohart 1991: 478 (China: Shanghai, cat., *ignita* group); Kurzenko and Lelej 2007: 1005 (China: Shanghai cat.).

######## Distribution.

China (Shanghai).

###### 16. Genus *Chrysura* Dahlbom, 1845

####### 165. Chrysura hirsuta

Taxon classificationAnimaliaHymenopteraChrysididae

(Gerstaecker, 1869)

http://species-id.net/wiki/Chrysis_hirsuta

Chrysis hirsuta Gerstaecker, 1869: 185. Holotype ♀, Austria: Ober-Kärnthen (depository: MNHU)*.
Chrysis davidi du Buysson, 1898a: 524. Holotype ♀, China: Jehol [= Johol] (depository: MNHN).
Chrysis davidi : du Buysson, 1899: 163 (China, cat.).
Chrysis (Holochrysis) davidi : Bischoff 1913: 38 (North China, cat.); Tsuneki 1948a: 125a (China: Shanxi: Hengshuichen-Hengligkuan, cat., distr.); 128 (China: Beijing distr., Manchuria, Shanxi, cat.); Tsuneki 1948b: 47 (North China: Rehe, Shanxi, tax.); 51 (résumé); Tsuneki 1950: 67 (comp. notes); Tsuneki 1953b: 24 (North China, (Johol, Shanxi), cat.); Linsenmaier 1959: 79 (synonym of *hirsuta*).
Chrysis (Chrysogona) hirsuta : Linsenmaier 1959: 79 (key, tax., descr., biol., *pustulosa* group), 202 (fig. 244).
Chrysis hirsuta : Móczár 1967: 73 (China, key, tax., descr., biol.).
Chrysura hirsuta : Kimsey and Bohart 1991: 490 (cat., *radians* group); Kurzenko and Lelej 2007: 1006 (China, cat.); Terayama et al. 2010: 4 (fig.), 9 (China, cat.), 12 (tab., biol.).

######## Material examined.

1♀, Kouy-Théou Cavalaire, 1921 (NMLS).

######## Distribution.

China (Liaoning, Hebei, Beijing, Shanxi, Guizhou). Central Europe and Fennoscandia, south European mountains, Korea, Japan (Linsenmaier 1959; Tsuneki 1953b).

######## Remarks.

Tsuneki (1948b) placed *Chrysis iwatai* Tosawa, 1942 in synonym of *Chrysis davidi* du Buysson. *Chrysis iwatai* is considered as a valid species in the genus *Chrysis* by Kimsey and Bohart (1991: 426). Tsuneki (1950: 67) provided a comparison of Chrysis (Holochrysis) davidi and *Chrysis koma* Tsuneki, 1950. *Osmia orientalis* (Hymenoptera, Megachilidae) is recorded as its host (Terayama et al. 2010).

####### 166. Chrysura refulgens

Taxon classificationAnimaliaHymenopteraChrysididae

(Spinola, 1806)

http://species-id.net/wiki/Chrysis_refulgens

Chrysis refulgens Spinola, 1806: 8. Holotype ♀, Italy (8 (descr.), depository: MRSN)*.
Chrysis artifex Smith, 1874b: 456. Holotype ♂, Hong Kong (456 (descr.), depository: BMNH) (synonymised by Kimsey and Bohart 1991).
Chrysis (Holochrysis) artifex : Mocsáry, 1889: 247 (tax., descr.), 248 (Hong Kong), Bischoff 1913: 37 (North China, cat.).
Chrysis artifex : Dalla Torre 1892: 44 (China, cat.).
Chrysura refulgens : Kimsey and Bohart 1991: 495 (cat., *radians* group).

######## Distribution.

China (Hong Kong). Widely distributed in the Mediterranean basin (Linsenmaier 1959).

###### 17. Genus *Euchroeus* Latreille, 1809

####### 167. Euchroeus mon golicus

Taxon classificationAnimaliaHymenopteraChrysididae

Tsuneki, 1947

http://species-id.net/wiki/Euchroeus_purpuratus_mongolicus

Plate 59

Euchroeus purpuratus f. mongolicus Tsuneki, 1947: 54. Holotype ♀, China: Inner Mongolia: Apaka (54 (descr., biol.), 55 (ecol.), depository: NIAS).
Euchroeus purpuratus mongolicus : Tsuneki 1948a: 124 (China: Manchuria, Nanpintsun, tax., descr.), 128 (Shanxi, cat.).
Euchroeus (Euchroeus) mongolicus : Linsenmaier 1959: 73 (Mongolia [= Inner Mongolia], tax., descr.), 200 (fig. 213).
Brugmoia quadrata (Shuckard, 1837): Kimsey and Bohart 1991: 296 (cat.).

######## Material examined.

Paratypes, 2♂♂2♀♀, Apaka, Inner Mongolia, 4.VI.1939, K. Tsuneki, *Euchroeus purpuratus mongolicus* m. (NMLS).

######## Distribution.

China (Inner Mongolia, Shanxi).

######## Host.

Possible host is *Podalonia caucasica* Morawitz (Hymenoptera, Sphecidae).

######## Remarks.

*Euchroeus mongolicus* was synonymised with *Brugmoia quadrata* (=*Euchroeus purpuratus*) by Kimsey and Bohart (1991). But, the generic name *Euchroeus* Latreille, 1809 and specific name *Chrysis purpurata* Fabricius, 1787 were conserved by ICZN (1998). As a result the generic name *Brugmoia* Radoszkowski, 1877 (used by Kimsey and Bohart (1991)) is a junior synonym of *Euchroeus* Latreille, 1809, and the name *quadrata* is a junior synonym of *purpurata*.

*Euchroeus mongolicus* is well characterized by the male’s colouration (similar to that of the female, in contrast with the green-blue colouration of the males belonging to this genus) and by its sparse punctuation, the smallest mandibles and shorter tongue compared with the typical *Euchroeus purpuratus*.

####### 168. Euchroeus orientis

Taxon classificationAnimaliaHymenopteraChrysididae

(Semenov-Tian-Shansky, 1909)

http://species-id.net/wiki/Pseudochrysis_purpurata_orientis

Plates 60 , 61

Pseudochrysis purpurata ssp. orientis Semenov-Tian-Shansky, 1909: 214 [*nec Euchroeus*]. Lectotype ♂ design. by Kimsey (in Kimsey and Bohart 1991: 296), Dzhungaria Chinense [= Xinjiang]: Bugas near Hami (depository: ZIN)*.
Euchroeus purpuratus var. orientalis (!): Bischoff 1913: 29 (Dzhungaria).
Brugmoia purpurata ssp. orientis : Kimsey and Bohart 1991: 296 (China: Dzhungaria Chinense [= Xinjiang]: Hami, cat.).

######## Material examined.

Paralectotype, 1♀, Dzhungaria Chinense [= Xinjiang]: Bugas near Hami, 7.IX.1895, expedition Kozlov (ZIN).

######## Distribution.

China (Xinjiang).

######## Remarks.

The oriental specimens examined (from China to Kyrgyzstan) are differentiated from the typical *Euchroeus purpuratus* (Fabricius, 1787) and are considered as a valid species. The coarse body sculpture and different colouration between the two demonstrate that they are different species.

###### 18. Genus *Praestochrysis* Linsenmaier, 1959

####### 169. Praestochrysis lachesis

Taxon classificationAnimaliaHymenopteraChrysididae

(Mocsáry, 1913)

http://species-id.net/wiki/Chrysis_lachesis

Plate 62

Chrysis (Pentachrysis) lachesis Mocsáry, 1913a: 7. Holotype ♂, Taiwan: Taihorisho (7 (descr.), depository: HNHM)*.
Chrysis (Pentachrysis) lachesis : Mocsáry 1913b: 619 (Taiwan, cat.); Bischoff 1913: 62 (Taiwan, cat.); Uchida 1927: 152 (Taiwan, cat.); Tsuneki 1955: 35 (key), 40 (tax., descr.), 41 (Taiwan: Taihorihsho); Tsuneki 1970b: 18 (Taiwan, cat.).
Chrysis (Pentachrysis) basilacuna Sugihara, 1932: 372. Type ?; Taiwan (372 (descr.), depository unknown).
Chrysis (Pentachrysis) basilacuna : Tsuneki 1955: 35 (key), 41 (tax., descr.), 42 (figs 7–14), 43 (Taiwan: Taihoku); Tsuneki 1970b: 18 (synonym of *lachesis*).
Praestochrysis lachesis : Kimsey and Bohart 1991: 532 (Taiwan, cat.).

######## Distribution.

China (Taiwan).

######## Remarks.

Tsuneki (1955) assumed that *Chrysis lachesis* was a synonym of *Chrysis basilacuna*. According to his description, the only distinguishing characteristic between the two species is the length of the antennal segments, which we think that could be a sexual dimorphic characteristic. Tsuneki was not able to study the type of *Chrysis lachesis* and postponed discussion of the possible synonymy. Later, Tsuneki (1970b) considered *Chrysis basilacuna* a synonym of *Chrysis lachesis* without further discussion, but he surely examined all of the material available in the area. Kimsey and Bohart (1991) did not examine the type of *Chrysis basilacuna* and placed it in the genus *Chrysis*. However, the anal margin of the third tergite with five teeth excludes this species from the genus *Chrysis* s. str., and we agree with Tsuneki’s interpretation.

####### 170. Praestochrysis ribbei

Taxon classificationAnimaliaHymenopteraChrysididae

(Mocsáry, 1889)

http://species-id.net/wiki/Chrysis_ribbei

Plate 63

Chrysis (Pentachrysis) ribbei Mocsáry, 1889: 524. Lectotype ♀ design. by Bohart (in Bohart and French 1986: 342), Celebes (depository: HNHM)*.
Chrysis (Pentachrysis) shanghaiensis var. ribbei : Bischoff 1910: 486 (China: Canton [= Guangzhou], cat.).
Chrysis (Pentachrysis) ribbei : Tsuneki 1955: 43 (possible syn. of *Praestochrysis shanghaiensis*).
Praestochrysis ribbei : Kimsey and Bohart 1991: 534 (cat.).

######## Distribution.

China (Guangdong). Indonesia, Thailand (Kimsey and Bohart 1991).

####### 171. Praestochrysis shanghaiensis

Taxon classificationAnimaliaHymenopteraChrysididae

(Smith, 1874)

http://species-id.net/wiki/Chrysis_shanghaiensis

Praestochrysis shanghaiensis *Chrysis Shanghaiensis* Smith, 1874b: 460. Holotype ♀, China: Shanghai (469 (descr.), depository: BMNH).
Chrysis (Pentachrysis) mandarina Mocsáry, 1889: 522. Holotype ♀, China: Ta-tschian-sy (522 (descr.), depository: HNHM) (synonymised by Kimsey and Bohart 1991: 534).
Chrysis (Pentachrysis) shanghaiensis : Mocsáry, 1889: 522 (China borealis, tax., descr.); Bischoff 1910: 486 (China [Shandong]: Kiautschou [= Jiaozhou Bay], cat.); Bischoff 1913: 63 (North China, cat.); Uchida 1927: 152 (North China, Taiwan, cat.); Tsuneki 1953a: 60 (South Manchuria [Liaoning]: Dairen [= Dalian], Tashonshan, tax.); Tsuneki 1955: 36 (key), 43 (tax.), 44 (descr., figs 15–20, 46 (China, Dairen [= Dalian], Taiwan, biol.); Tsuneki 1970b: 18 (Taiwan: Penpuchi, Kuanfu, cat.); Tsuneki 1970c: 49 (eastern part of China, Manchuria, North China, key, tax.).
Chrysis shanghaiensis : Dalla Torre 1892: 95 (China borealis, cat.); du Buysson 1898c: 82 (China: Tché-li, Han-Kèou, Shanghai, tax., biol., morphology), pl. 1 (figs 1–7); du Buysson 1901a: 29 (China: Tché-li, Shanghai, tax., biol.); Seurat 1901: 236 (Shanghai, tax., biol.); Bingham 1903: 438 (key), 477 (tax., descr.), 478 (China, distr.); Hammer 1950: 1 (China: Kiansu [= Jiangsu], cat.).
Praestochrysis shanghaiensis : Kimsey and Bohart 1991: 534 (China, cat.); He et al. 2004: 889 (cat.); Kurzenko and Lelej 2007: 1006 (China, Taiwan, cat.); Terayama et al. 2010: 6 (figs.), 9 (China, Taiwan, cat.), 12 (tab., biol.).

######## Material examined.

1♂, China, 1947, Ming Po, Coll. Linsenmaier; 1♀ / J. de Joannis / with pinned cocoon; 1 ♂, [Hori, Formosa, 25.V.’32 / L. Gressitt Collection; 1 ♂, China, Ningpo, 1. –5.7.1934, Naef; 2 ♀♀, Chusan [= Zhoushan, Zhejiang] China, Juni, 1948, Collect. Naef. All the specimens were identified by Linsenmaier (NMLS).

######## Distribution.

China (Liaoning, Jiangsu, Shandong, Jiangsu, Shanghai, Zhejiang, Taiwan, Jiangxi, Hubei, Hunan). Japan, Indin (He et al. 2004).

######## Host.

*Monema flavescens* (Lepidoptera, Limacodiidae) (du Buysson 1898c, 1901a, Seurat 1901). Various studies on the parasitism by *Praestochrysis shanghaiensis* have been published (Yamada 1980, 1987a, 1987b, 1988, 1990, 1991; Komeda and Hisamatsu 2005).

###### 19. Genus *Primeuchroeus* Linsenmaier, 1968

####### 172. Primeuchroeus crassiceps

Taxon classificationAnimaliaHymenopteraChrysididae

(Tsuneki, 1970)

http://species-id.net/wiki/Chrysis_crassiceps

Chrysis (Chrysura) crassiceps Tsuneki 1970b: 8. Holotype ♀, Taiwan: Chiai Province: Kuanhua (8 (tax., descr., figs 12–22), 9 (Taiwan: Kuanhua, descr.), depository: NIAS).
Primeuchroeus crassiceps : Bohart 1988: 22 (fig.1), 23 (key); Kimsey and Bohart 1991: 541 (Taiwan: Chiai Prov.: Kuanhua, cat., *siamensis* group); Wei et al., 2014a: 45 (key, tax., descr.), 46 (figs 1–2, ♀), 47 (figs 3–9, ♀, *siamensis* group), 48 (figs 10–11, ♂), 49 (figs 12–18, ♂).

######## Distribution.

China (Taiwan, Yunnan).

####### 173. Primeuchroeus kansitakuanus

Taxon classificationAnimaliaHymenopteraChrysididae

(Tsuneki, 1970)

http://species-id.net/wiki/Chrysis_kansitakuanus

Chrysis kansitakuanus Tsuneki 1970b: 9. Holotype ♀, Taiwan: Chiai Province: Kansitaku (9 (tax., descr.), 10 (Taiwan, Kansitaku, descr., figs 23–26, comp. notes), depository: OMNH, not NIAS).
Primeuchroeus kansitakuanus : Bohart 1988: 22 (fig.2), 23 (key); Kimsey and Bohart 1991: 542 (*ghilianii* group); Wei et al. 2014a: 45 (key), 48 (China: Zhejiang: Lin’an, Mt. Qingliangfeng; Fujian: Da’an; Hubei, Jingmen, Jingshan; Hunan: Mt. Huping, Shinianzigou; Mt. Huping, Shinianzigou; Mt. Huping, Zongfeng; Mt. Huping, Shuawu village; Huaihua; Guangzhou: Wangzishan Forest Park; Liuxihe Forest Park; Guangdong: Chebaling National Nature Reserve; Hainan: Mt. Wuzhi; Guizhou: Tianzhu; Mayang River, Dahe Dam; Yunnan: Jinggu, Yunhai Reserve; Yingjiang; Chenggong, Luoyang, tax.), 50 (figs 19–20, ♀), 51 (figs 21–27, ♀, descr.), 52 (biol., *ghiliani* group).

######## Distribution.

China (Zhejiang, Fujian, Taiwan, Hubei, Hunan, Guangdong, Hainan, Guizhou, Yunnan). Malaysia, Viet Nam (Kimsey and Bohart 1991).

####### 174. Primeuchroeus yongdaerianus

Taxon classificationAnimaliaHymenopteraChrysididae

Kim, 2013

http://species-id.net/wiki/Primeuchroeus_yongdaerianus

Primeuchroeus yongdaerianus Kim, 2013: 95. Holotype ♀, Korea: Inje-gun, Buk-myeon, Yongdae-ri (95 (tax., descr.), 96 (figs 1A–1D, ecol., comp. notes).
Primeuchroeus yongdaerianus : Wei et al. 2014a: 45 (key), 52 (China: Yunnan: Gaoligongshan National Nature Reserve; Mailongxia, tax., descr.), 53 (figs 28–29), 54 (figs 30–36), 55 (biol., *siamensis* group).

######## Distribution.

China (Yunnan). Korea (Kim 2013).

###### 20. Genus *Pseudospinolia* Linsenmaier, 1951

####### 175. Pseudospinolia humboldti

Taxon classificationAnimaliaHymenopteraChrysididae

(Dahlbom, 1845)

http://species-id.net/wiki/Chrysura_humboldti

Chrysura humboldti Dahlbom, 1845: 6. Holotype ♂, Rhodes (6 (descr.), depository: NHRS)*.
Pseudochrysis humboldti : Tsuneki 1953a: 58 (China, tax.).
Euchroeus (Pseudospinolia) humboldti : Linsenmaier 1959: 67 (key, descr.), 201 (fig. 227).
Pseudospinolia humboldti : Kimsey and Bohart 1991: 547 (cat., distr.).

######## Distribution.

China (Shanxi). Widely distributed in the Palaearctic Region (Kimsey and Bohart, 1991).

####### 176. Pseudospinolia incrassata

Taxon classificationAnimaliaHymenopteraChrysididae

(Spinola, 1838)

http://species-id.net/wiki/Chrysis_incrassata

Chrysis incrassata Spinola, 1838: 454. Syntypes ♀♀, Corse (454 (descr.), depository: MRSN)*.
Pseudochrysis incrassata : Tsuneki 1953a: 57 (Manchuria [Liaoning]: Dairen [= Dalian], cat., distr.).
Euchroeus (Pseudospinolia) incrassata : Linsenmaier 1959: 67 (key, tax.), 68 (distr., descr.), 200 (fig. 207).
Pseudospinolia incrassata : Kimsey and Bohart 1991: 546 (fig. 138f), 547 (cat., distr.).

######## Distribution.

China (Liaoning). Widely distributed in the Palaearctic Region (Linsenmaier 1959).

####### 177. Pseudospinolia neglecta

Taxon classificationAnimaliaHymenopteraChrysididae

(Shuckard, 1837)

http://species-id.net/wiki/Chrysis_neglecta

Chrysis neglecta Shuckard, 1837: 169. Lectotype ♀ design. by Morgan (1984: 9), England (depository: BMNH).
Pseudochrysis neglecta : Trautmann 1927: 92 (key), 99 (Tianshan [Xinjiang ?], descr., biol., distr.).
Euchroeus (Pseudospinolia) neglectus : Linsenmaier 1959: 65 (key), 66 (tax., descr.), 200 (fig. 206).
Pseudospinolia neglecta : Kimsey and Bohart 1991: 546 (fig. 138c), 548 (cat.); Rosa 2006: 30 (biol.), 32 (biol.), 40 (ecol.), 52 (ecol.), 55 (biogeogr.), 78 (cat.), 200 (China, tax., descr., biol.).

######## Distribution.

China (Xinjiang?). Widely distributed in the Palaearctic Region (Bohart and Kimsey 1991).

###### 21. Genus *Stilbum* Spinola, 1806

####### 178. Stilbum calens

Taxon classificationAnimaliaHymenopteraChrysididae

(Fabricius, 1781)

http://species-id.net/wiki/Chrysis_calens

Chrysis calens Fabricius, 1781: 455. Holotype ♀, Siberia (455 (descr.), depository: BMNH)*.
Stilbum splendidum var. calens : du Buysson 1900: 156 (China: Beijing, cat.).
Stilbum cyanurum var. calens : Hammer 1936: 3 (China [Inner Mongolia]: Hurtjertu Gol, tax.).
Stilbum cyanurum cyanurum f. calens (!): Tsuneki 1947: 53 (China [Inner Mongolia]: Apaka); Tsuneki 1948a: 124 (China: Shanxi: Nanpintsun, Manchuria: Tonei, tax., distr.), 128 (Shanxi, cat.); Tsuneki 1953a: 58 (Manchuria [Liaoning]: Dairen [= Dalian], Tungning, cat., distr.).
Stilbum calens ssp. zimmermanni Linsenmaier, 1959: Linsenmaier 1968: 123 (China, cat.).
Stilbum calens ssp. wesmaeli Dahlbom, 1845: Linsenmaier 1997b: 134 (China, tax., descr.).

######## Material examined.

1♂, Valley of the river Kuldgi, V.VI.1878, leg. Regel (ZIN). 1♂, Alashan, Din-yuan-in, 8.–9.VI.1908, expedition Kozlov (ZIN).

######## Distribution.

China (Liaoning, Beijing, Inner Mongolia, Shanxi). Widely distributed in the Palaearctic Region (Tsuneki 1948a; Linsenmaier 1959).

####### 179. Stilbum cyanurum

Taxon classificationAnimaliaHymenopteraChrysididae

(Forster, 1771)

http://species-id.net/wiki/Chrysis_cyanura

Chrysis cyanura Forster, 1771: 89. Holotype ♂, Spain (89 (descr.), depository: BMNH).
Chrysis splendida Fabricius, 1775: 357. Syntypes, Australia (357 (descr.), depositories: BMNH, ZMU)*.
Chrysis amethystina Fabricius, 1775: 359. Syntypes, Australia (359 (descr.), depositories: BMNH, ZMU).
Stilbum splendidum : Smith 1864: 144 (China, tab.); Radoszkovsky 1866: 14 (China, cat.); du Buysson 1900: 156 (China: Hong Kong, cat.).
Stilbum splendidum var. caspicum du Buysson (In André), 1896: 680. Syntypes, Turkmenistan: Otreck, Ethiopia: Abissinia (680 (descr.), depository: ISEA-PAS, MNHN)*.
Stilbum cyanurum : du Buysson 1898a: 544 (China: Kiang-si [= Jiangxi], cat.); du Buysson 1899: 169 (China, cat.); Hammer 1936: 3 (China [Inner Mongolia]: Hurtjertu Gol, cat.); Tsuneki 1948b: 50 (Manchuria, North China, Taiwan, tax.); Kimsey and Bohart 1991: 567 (cat.), Kurzenko and Lelej 2007: 1006 (China, cat.); Terayama et al. 2010: 6 (fig.), 9 (China, Taiwan, cat.), 12 (tab., biol.), 13 (fig. 4).
Stilbum splendidum var. caspicum : du Buysson 1898a: 561 (China, cat.); Zimmermann 1937: 657 (tax.).
Stilbum splendidum var. amethystinum : Mocsáry 1913b: 613 (tax.), 614 (Taiwan, cat.); du Buysson 1898a: 561 (China, cat.); Uchida 1927: 151 (Taiwan, China, cat.); Yasumatsu 1938: 73 (China, cat.).
Stilbum cyanurum var. auratum Trautmann, 1920: 240. Holotype ♀, China (240 (descr.), depository: MNHU); Trautmann 1927: 81 (China, tax.), 82 (Central China, Kansu, tax.).
Stilbum cyanurum var. splendidum : Uchida 1927: 151 (Taiwan, cat.); Tsuneki 1970b: 19 (cat.), 20 (Taiwan: Ilan Province: Chuantou, Tsukeng; Hualien Province: Liyuchih; Nantou Province: Puli, Jihyuehtan; Taitung Province: Chihpenchi; Pingtung Province: Checheng, Kentin Park, tax.).
Stilbum cyanurum cyanurum : Zimmermann 1937: 650 (East China, tax., distr.), 656 (Central China, type); Tsuneki 1947: 52 (China: Shanxi, tax.); *Stilbum cyanurum*: Hammer 1950: 1 (China: Kiangsu [= Jiangsu], tax.); Tsuneki 1953a: 24 (North China, Manchuria, tax.); *Stilbum cyanurum cyanurum*: Tsuneki 1953b: 58 (Manchuria: Chinchow, cat.); Linsenmaier 1959: 180 (key, tax.), 181 (China, descr.), 216 (fig. 686); *Stilbum cyanurum cyanurum*: Móczár 1967: 117 (key, tax., descr.), 118 (Manchuria, biol.).
Stilbum cyanurum f. auratum : Zimmermann 1937: 656 (Central China, tax.).
Stilbum calens ssp. auratum : Linsenmaier 1959: 182 (China, Kansu, tax.).

######## Material examined.

Guandong: 1♂, Canton [= Guangzhou], 1910; Yunnan: 2♂♂, Ta-pin-tze, leg. R.P. Delavay; 1♀, Macao; 1♂, Taiwan, Takao, 1923; 1♀, id., 29.IX.1907; 1♀, Taihanroku; 1♀, Taipei, 1.V.1976; all the specimens identified by Linsenmaier (NMLS). 1♂, Alashan, Din-yuan-in, 17. –20.VIII.1908, leg. Kozlov (ZIN). 62 exx. under the name *Stilbum cyanurum auratum* housed in ZIN with the following labels:, Din-yuan-in, Alashan, 17.–18.VI.1908 and 22.IV.1909, leg. Kozlov; Alashan, Golih-Goli Canyon; Alashan, Shan-Shun Canyon, 17. –18.VI.1908, leg. Kozlov.

######## Distribution.

China (Liaoning, Inner Mongolia, Shanxi, Gansu, Shandong, Jiangsu, Taiwan, Jiangxi, Guangdong, Hong Kong, Macao, Yunnan). Widely distributed in the Oriental, Palaearctic, Afrotropical and Australian Regions (Kimsey and Bohart 1991).

###### 22. Genus *Trichrysis* Lichtenstein, 1876

####### 180. Trichrysis cyanea

Taxon classificationAnimaliaHymenopteraChrysididae

(Linnaeus, 1758)

http://species-id.net/wiki/Sphex_cyanea

Sphex cyanea Linnaeus, 1758: 572. Lectotype ♂ design. by Morgan (1984: 10), Europe (depository: LSL).
Chrysis (Trichrysis) cyanea : Tsuneki 1947: 55 (China: Beijing, cat., distr.); Tsuneki 1950: 70 (comp. notes); Tsuneki 1953a: 58 (Manchuria, Kaiyüan, Shorei, Yiyasaka, tax., distr.); Tsuneki 1953b: 25 (North China, Manchuria, tax.); Linsenmaier 1959: 169 (key), 170 (tax., descr., biol.), 205 (fig. 383).
Trichrysis cyanea : Kimsey and Bohart 1991: 571 (cat.).

######## Distribution.

China (Liaoning, Beijing). Widely distributed in Europe and western Asia to Siberia, Russian Far East, Korea and Japan (Kurzenko and Lelej 2007).

####### 181. Trichrysis imperiosa

Taxon classificationAnimaliaHymenopteraChrysididae

(Smith, 1874)

http://species-id.net/wiki/Chrysis_imperiosa

Chrysis imperiosus (!) Smith, 1874b: 460. Lectotype ♀ design. by Bohart (in Kimsey and Bohart 1991: 533), Australia: Queensland, Moreton Bay (460 (descr.), depository: BMNH).
Chrysis imperiosa : du Buysson 1898a: 536 (“Montagnes de Song-Chaï”, cat.); du Buysson 1899: 168 (China, cat.).
Chrysis (Pentachrysis) imperiosa : Mocsáry 1913b: 619 (Taiwan: Juhosho, cat.); Uchida 1927: 152 (Taiwan, cat.); Tsuneki 1970b: 18 (Taiwan, cat.).
Chrysis (Trichrysis) imperiosa : Linsenmaier 1959: 170 (tax., *lusca* group); Linsenmaier 1994: 193 (tax.).
Praestochrysis imperiosa : Strumia 1996: 62 (Taiwan, tax.).

######## Distribution.

China (Taiwan). Austrialia, Thailand (Smith, 1874b; Tsuneki, 1963b).

######## Remarks.

Linsenmaier (1994, 1997a) and Madl and Rosa (2012) moved the species group *lusca* (including *lusca* and *imperiosa*) from the genus *Praestochrysis* Linsenmaier to genus *Trichrysis* Lichtenstein. Kimsey and Bohart (1991) treated *Praestochrysis imperiosa* (Smith, 1874) as a junior synonym of *Praestochrysis lusca* (Fabricius, 1804). But *Praestochrysis imperiosa* is distinctly different from *Praestochrysis lusca* (Fabricius) (Strumia 1996).

####### 182. Trichrysis lusca

Taxon classificationAnimaliaHymenopteraChrysididae

(Fabricius, 1804)

http://species-id.net/wiki/Chrysis_lusca

Plate 64

Chrysis lusca Fabricius, 1804: 171. Holotype ♀, Italy (accidentally introduced) (171 (descr.), depository: ZMU)*.
Chrysis lusca : du Buysson 1898a: 536 (Macao, cat.); du Buysson 1899: 168 (China, cat.).
Chrysis (Pentachrysis) lusca : Bischoff 1910: 486 (Taiwan, cat.); Mocsáry 1913a: 11 (Taiwan, cat.); Mocsáry 1913b: 619 (Taiwan, cat.); Uchida 1927: 152 (Taiwan, cat.); Tsuneki 1953b: 28 (Taiwan, cat.); Tsuneki 1955: 35 (key), 36 (tax.), 37 (figs 1–6), 38 (East China, Taiwan, distr.); Tsuneki 1970b: 17 (tax.), 18 (Taiwan, Provinces of Taipei, Taoyuan, Illan, Huallien, Nantou, Chiai, Taitung, Pingtung, cat.); Tsuneki 1970c: 49 (Taiwan, key, tax.).
Pentachrysis lusca : Hammer 1950: 2 (China: Peitaiho, Taiwan, cat.).
Praestochrysis lusca : Kimsey and Bohart 1991: 533 (cat.); Kurzenko and Lelej 2007: 1006 (China, cat.).
Chrysis (Trichrysis) lusca : Linsenmaier 1994: 193 (tax.).

######## Material examined.

2♀♀, Taiwan, Chipon, VIII.1935 leg. K. Iwata, det. Enslin; 1♀, Taiwan, Sozan, VIII.1935, leg. K. Iwata det. Linsenmaier (NMLS).

######## Distribution.

China (Hebei, Taiwan, Macao). Widely distributed in the Palaearctic and Oriental Regions (Kimsey and Bohart 1991).

######## Remarks.

Linsenmaier (1994, 1997a) and Madl and Rosa (2012) moved the species group *lusca* (including *lusca* and *imperiosa*) from the genus *Praestochrysis* Linsenmaier to the genus *Trichrysis* Lichtenstein.

####### 183. Trichrysis luzonica

Taxon classificationAnimaliaHymenopteraChrysididae

(Mocsáry, 1889)

http://species-id.net/wiki/Chrysis_luzonica

Chrysis (Trichrysis) luzonica Mocsáry, 1889. 328. Holotype ♀, Philippines: Luzon (328 (descr.), depository: ISEA-PAS).
Chrysis (Trichrysis) taial Tsuneki, 1970b: 11. Holotype ♀, Taiwan; Nanton Province: Puli (11 (tax., descr.), 12 (Taiwan, Puli; Tsukeng, Chantou, Sschungchi, Manchou, Oluampi, Kentin Park, Fanshanlu, descr., figs 30–34), depository: OMNH, not NIAS) (synonymised by Kimsey and Bohart 1991: 572).
Trichrysis luzonica : Bohart 1987: 348 (Taiwan, key); Kimsey and Bohart 1991: 572 (Hong Kong, cat.).

######## Distribution.

China (Taiwan, Hong Kong). Philippines (Mocsáry 1889).

####### 184. Tric hrysis pellucida

Taxon classificationAnimaliaHymenopteraChrysididae

(du Buysson, 1887)

http://species-id.net/wiki/Chrysis_pellucida

Chrysis pellucida du Buysson, 1887: 183. Syntypes ♂♀, China, Turkey (183 (descr.), 184 (China), depository: MNHN).
Chrysis (Trichrysis) buyssoni Mocsáry, 1889. 323. Replacement name for *Chrysis pellucida* du Buysson 1887*nec Brugmoia pellucida* Radoszkowski, 1877.
Chrysis pellucida : du Buysson, 1898a: 525 (China: Jehol [= Johol]: “Nord de Pèking”, cat.); du Buysson, 1899: 164 (China, cat.); du Buysson, 1900: 144 (China: Beijing, cat.).
Chrysis (Trichrysis) pellucida : Bischoff, 1913: 46 (China, cat.); Tsuneki 1953b: 25 (North China: Jehol, cat.); Linsenmaier 1959: 169 (China, key, tax.).
Chrysis (Monochrysis) coreana Uchida, 1927: Tsuneki 1948b: 47 (tax., syn.); 51 (résumé);
Trichrysis buyssoni : Kimsey and Bohart 1991: 571 (cat.).

######## Distribution.

China (Inner Mongolia, Hebei, Beijing). Middle East to China and Russian Far East (Kimsey and Bohart 1991; Kurzenko and Lelej 2007).

######## Remarks.

Chrysis (Trichrysis) buyssoni Mocsáry was a replacement name for *Trichrysis pellucida* (du Buysson, 1887). However, after 1889, the name *Brugmoia pellucida* Radoszkowski was considered belonging to the genus *Euchroeus* Latreille and no longer congeneric. According to the Code (Art. 59), a junior secondary homonym replaced before 1961 is permanently invalid unless the substitute name is not in use and the relevant taxa are no longer considered congeneric, in which case the junior homonym is not to be rejected on grounds of that replacement.

####### 185. Trichrysis secernenda

Taxon classificationAnimaliaHymenopteraChrysididae

(Mocsáry, 1912)

http://species-id.net/wiki/Chrysis_secernenda

Plate 65

Chrysis (Trichrysis) secernenda Mocsáry, 1912a: 376. Lectotype ♂ design. by Bohart (in Bohart and French 1986: 342), Uzbekistan: Gouldsha (type series: China: Xinjiang, paralectotypes) (depository: HNHM)*.
Trichrysis secernenda : Kimsey and Bohart 1991: 573 (cat.).

######## Distribution.

China (Xinjiang).

####### 186. Trichrysis triacantha

Taxon classificationAnimaliaHymenopteraChrysididae

(Mocsáry, 1889)

http://species-id.net/wiki/Chrysis_triacantha

Chrysis (Trichrysis) triacantha Mocsáry, 1889: 325. Holotype ♀, Indonesia: Sumatra (325 (descr.), depository: NHMW)*.
Chrysis (Trichrysis) formosana Mocsáry, 1912a: 380. Lectotype ♀ design. by Bohart (in Bohart and French 1986: 341), Taiwan: Takao [= Kaohsiung] (HNHM)* (synonymised by Kimsey and Bohart 1991: 574).
Chrysis (Trichrysis) sauteri Mocsáry, 1912a: 381. Holotype ♂, Taiwan: Takao [= Kaohsiung] (381 (descr.), depository: HNHM)* (synonymised by Kimsey and Bohart 1991: 574).
Chrysis (Trichrysis) formosana : Bischoff 1913: 45 (Taiwan, cat.); Mocsáry 1913b: 614 (Taiwan: Takao [= Kaohsiung], Kankau, tax.), 619 (Taiwan, cat.)). Uchida 1927: 151 (Taiwan, cat.); Uchida 1933: 3 (Taiwan, cat.); Tsuneki 1970b: 10 (tax.), 11 (Taiwan: Chantou, Tsukeng, Penpuchi, Jihjuetan, Chulu, Chichpenchi, Ssuchungchi, Manchou, Fanshanlu, Kentin, Uluampi, descr.), 12 (comp. notes, figs 27–29).
Chrysis (Trichrysis) sauteri Mocsáry, 1913b: 614 (Taiwan, cat.); Mocsáry, 1913c: 289 (Taiwan, cat.); Uchida 1927: 151 (Taiwan, cat.); Tsuneki 1970b: 13 (Taiwan, tax.).
Chrysis (Trichrysis) tonkinensis Mocsáry, 1914: 25. Holotype ♀ [not ♂], Viet Nam: Tonkin (25 (descr.), depository: HNHM)* (synonymised by Kimsey and Bohart 1991: 574).
Chrysis (Trichrysis) tonkinensis var. cyanescens Mocsáry, 1914: 26. Holotype ♀, China: Poo Chow [= Fujian] (26 (descr.), depository: BMNH) (synonymised by Kimsey and Bohart 1991: 574).
Chrysis (Trichrysis) bicarinata Tsuneki, 1950: 69. Holotype ♀, Hong Kong (69 (descr.), depository: 70 (comp. notes), EIHU) (synonymised by Kimsey and Bohart 1991: 574).
Chrysis (Trichrysis) tonkinensis : Linsenmaier 1959: 169 (China, tax., descr.); Tsuneki 1961: 374 (Hong Kong, tax., descr., figs 19–21).
Trichrysis triacantha : Kimsey and Bohart 1991: 573 (Oriental: widespread, cat.); Terayama et al. 2010: 4 (fig.), 9 (Taiwan, cat.), 12 (tab., biol).

######## Distribution.

China (Fujian, Taiwan, Hong Kong). Widely distributed in the Oriental Region (Kimsey and Bohart 1991).

####### 187. Trichrysis trigona

Taxon classificationAnimaliaHymenopteraChrysididae

(Mocsáry, 1889)

http://species-id.net/wiki/Chrysis_trigona

Plate 66

Chrysis (Trichrysis) trigona Mocsáry, 1889: 327. Holotype ♀, Celebes: Bonthain (327 (descr.), depository: HNHM)*.
Trichrysis trigona : Kimsey and Bohart 1991: 574 (Hong Kong, cat.).

######## Distribution.

China (Hong Kong). Indonesia, Laos (Kimsey and Bohart 1991).

#### Subfamily Parnopinae

##### 23. Genus *Parnopes* Latreille, 1797

###### 188. Parnopes popovi

Taxon classificationAnimaliaHymenopteraChrysididae

Eversmann, 1857

http://species-id.net/wiki/Parnopes_popovi

Parnopes popovi Eversmann, 1857: 567. Holotype ♀, Siberia (567 (descr.), depository: ISEA-PAS)*.
Parnopes sinensis Smith, 1874b: 454. Holotype ♂, China: Shanghai (454 (descr.), depository: BMNH) (synonymised by Mocsáry 1889).
Parnopes popovii (!): Mocsáry 1889: 614 (China: Shanghai, Tschifu, Ta-tschian-sy, descr., distr.); Dalla Torre 1892: 112 (China, cat.).
Parnopes popovi : du Buysson (in André) 1896: 689 (China septentrionalis [Shandong]: Tschi-fu, cat.); Tsuneki 1953a: 58 (Manchuria [Heilongjiang]: Harbin, Yayasaka, cat., distr.); Tsuneki 1953b: 24 (North China, Manchuria, tax.); Kimsey 1987a: 86 (key), 89 (cat.); Kimsey and Bohart 1991; Kurzenko and Lelej 2007: 1005 (China, cat.).

####### Material examined.

1 ex., Imian Station, along the East Chinese railway, 18.VII.1914 (ZIN).

####### Distribution.

China (Heilongjiang, Shanghai, Shandong). Siberia and Korea (Tsuneki 1953b).

## Plates

**Plate 1.**
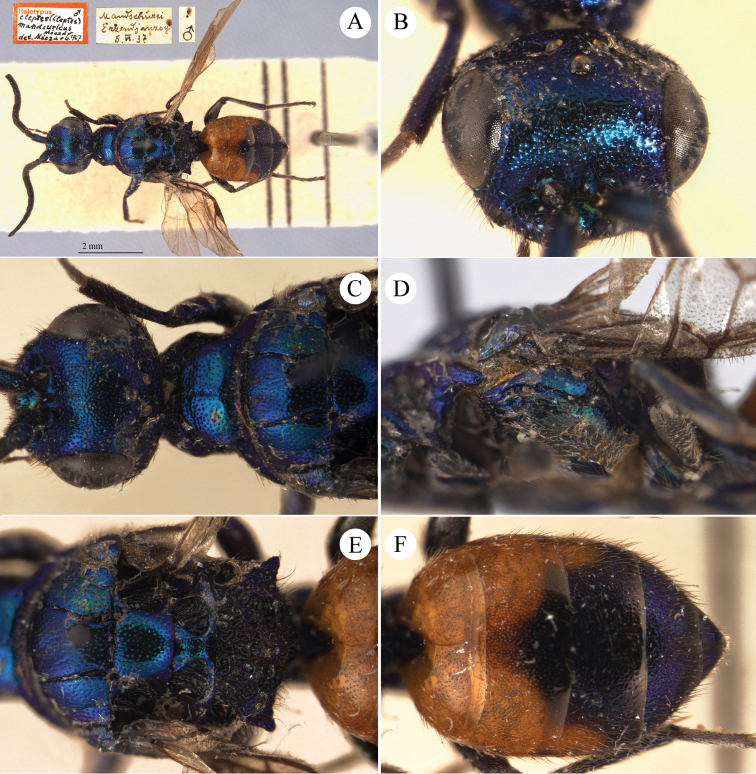
*Cleptes mandsuricus* Móczár, holotype. **A** Habitus, dorsal view **B** head, frontal view **C** head and anterior part of mesosoma, dorsal view **D** mesopleuron, lateral view **E** mesosoma, dorsal view **F** metasoma, dorsal view.

**Plate 2.**
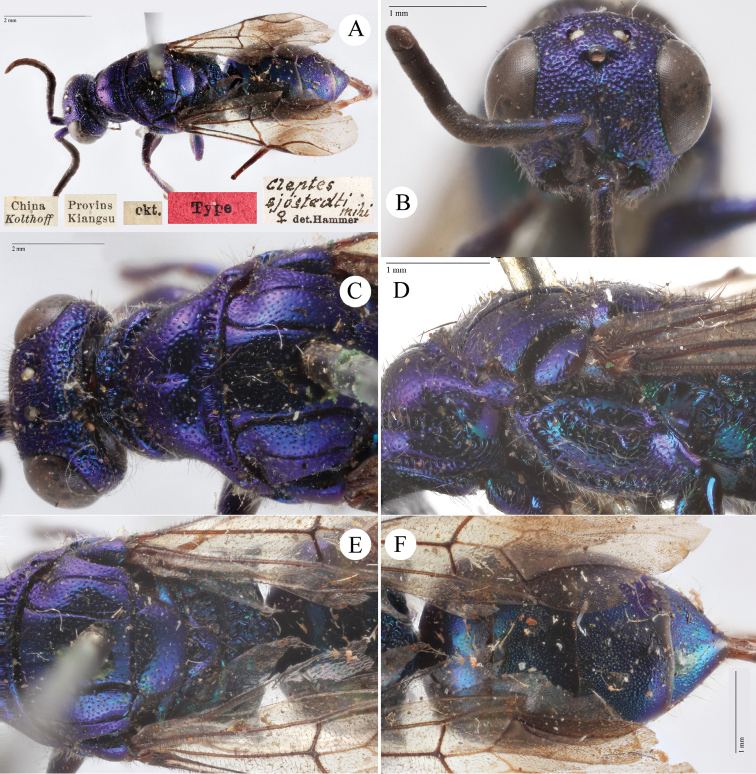
*Cleptes sjostedti* Hammer, holotype. **A** Habitus, dorso-lateral view **B** head, frontal view **C** head and anterior part of mesosoma, dorsal view **D** mesopleuron, lateral view **E** mesonotum, metanotum and propodeum, dorsal view **F** metasoma, dorsal view.

**Plate 3.**
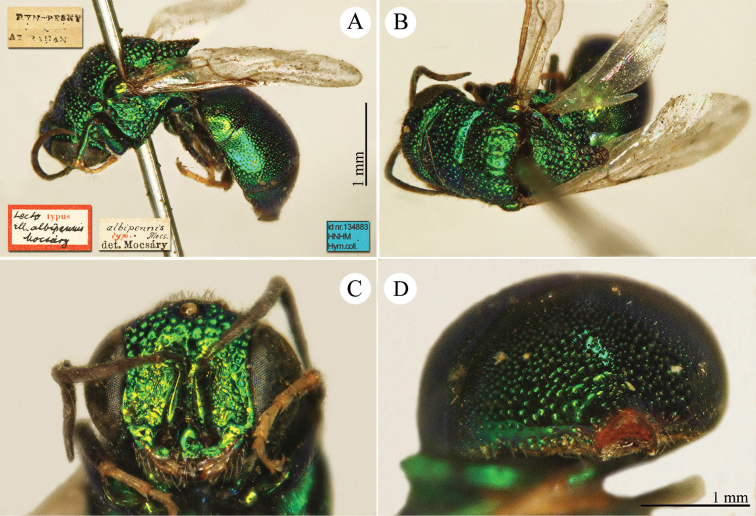
*Elampus albipennis* (Mocsáry), lectotype. **A** Habitus, lateral view **B** habitus, dorsal view **C** head, frontal view **D** mesosoma, posterior view.

**Plate 4.**
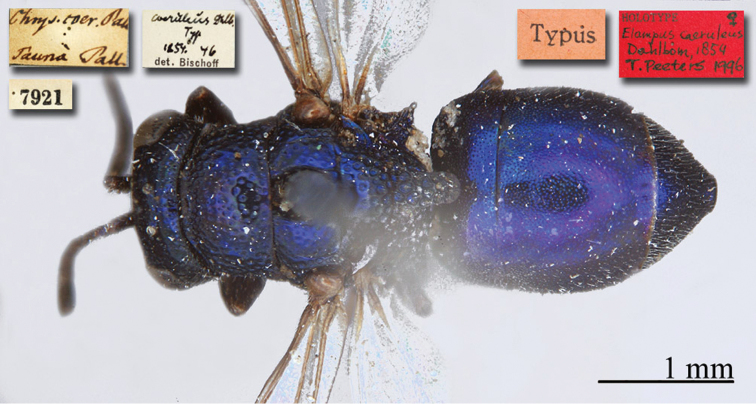
*Elampus coeruleus* Dahlbom, syntype, habitus, dorsal view.

**Plate 5.**
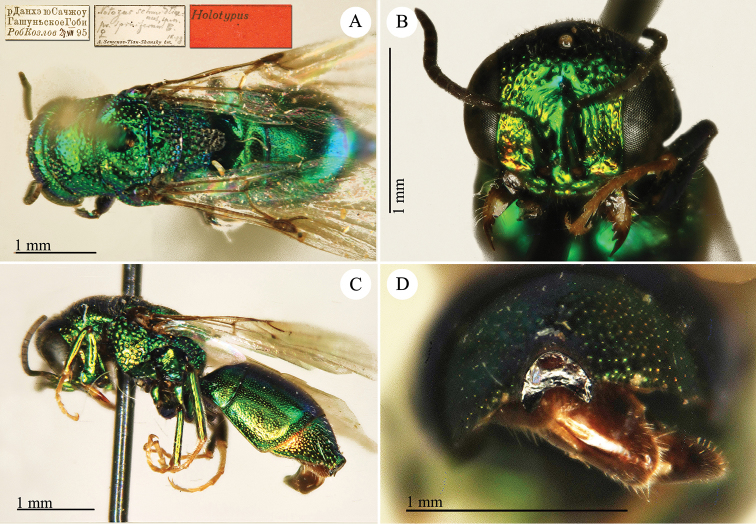
*Elampus schmidtianus* (Semenov-Tian-Shanskij), holotype. **A** Habitus, dorsal view **B** head, frontal view **C** habitus, lateral view **D** third metasomal tergite, posterior view.

**Plate 6.**
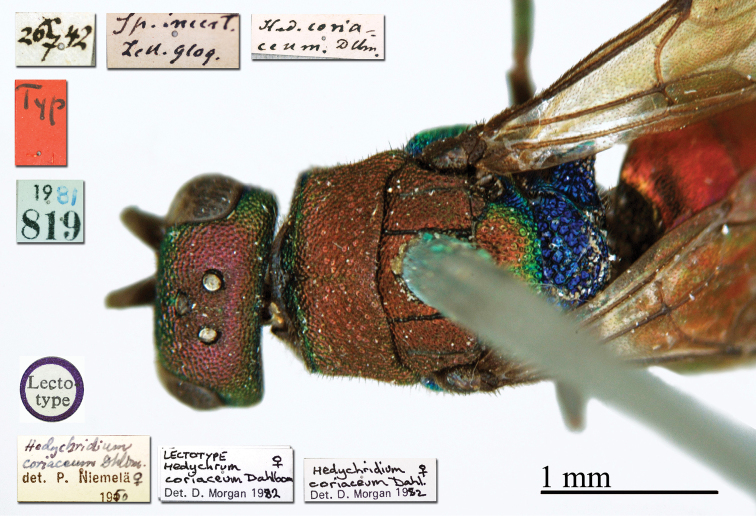
*Hedychridium coriaceum* (Dahlbom), lectotype, head and mesosoma, dorsal view.

**Plate 7.**
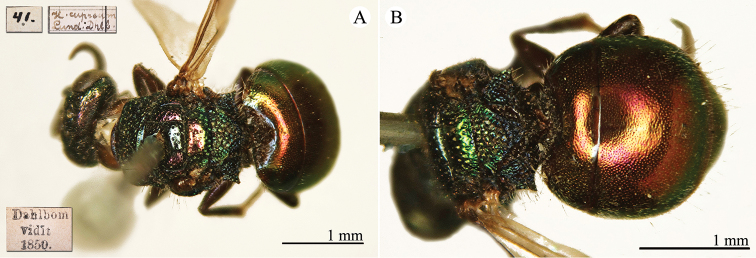
*Hedychridium cupreum* (Dahlbom), lectotype. **A** Habitus, dorsal view **B** metanotum, propodeum and metasoma, dorsal view.

**Plate 8.**
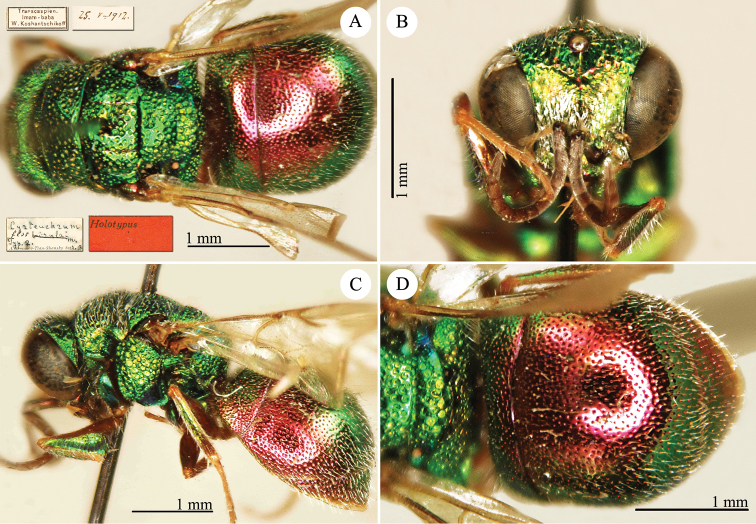
*Hedychridium flos* (Semenov-Tian-Shanskij), holotype. **A** Habitus, dorsal view **B** head, frontal view **C** habitus, lateral view **D** metasoma, dorsal view.

**Plate 9.**
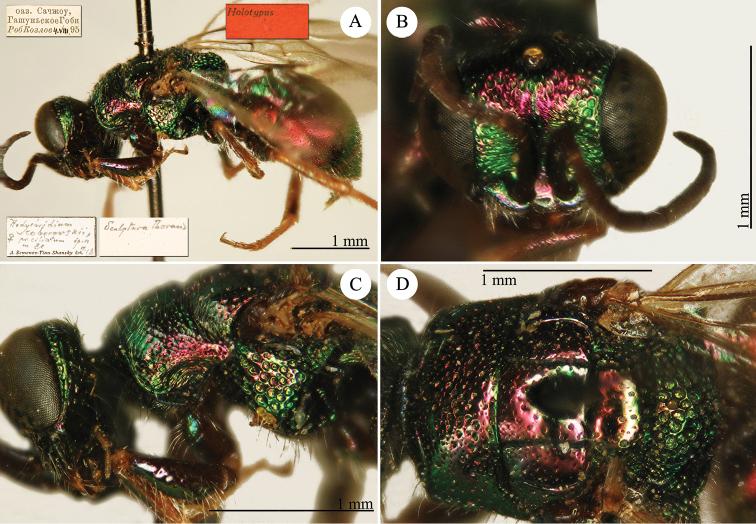
*Hedychridium roborovskii* Semenov-Tian-Shanskij, holotype. **A** Habitus, lateral view **B** head, frontal view **C** head and mesosoma, lateral view **D** mesosoma, dorsal view.

**Plate 10.**
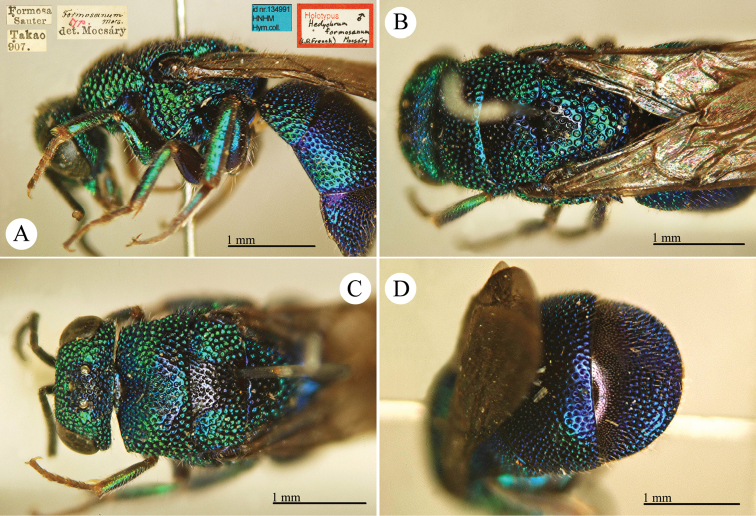
*Hedychrum formosanum* Mocsáry, holotype. **A** Habitus, lateral view **B** habitus, dorsal view **C** head and mesosoma, dorsal view **D** second and third metasomal tergites, dorso-lateral view.

**Plate 11.**
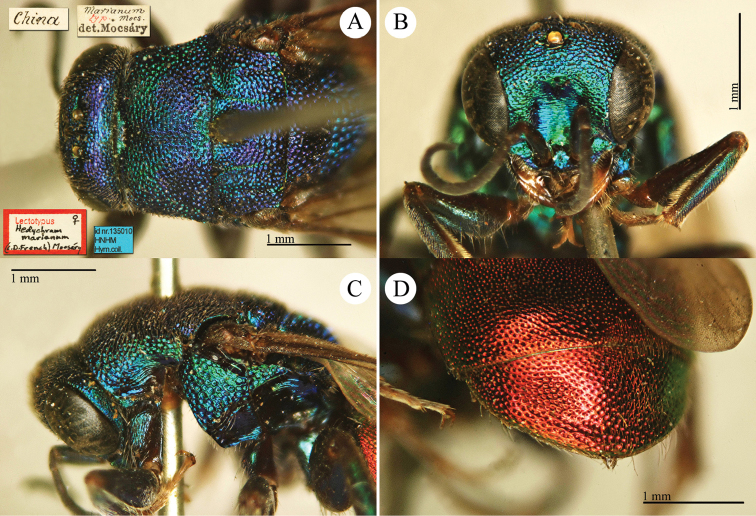
*Hedychrum marianum* Mocsáry, lectotype. **A** Head and mesosoma, dorsal view **B** head, frontal view **C** head and mesosoma, lateral view **D** second and third metasomal tergites, dorso-lateral view.

**Plate 12.**
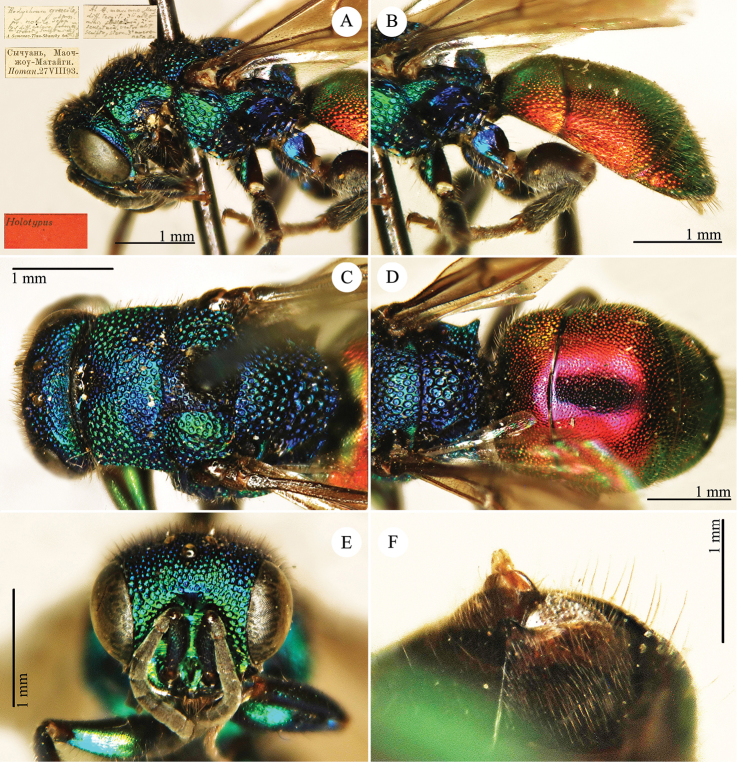
*Hedychrum gracile* Semenov-Tian-Shanskij, holotype. **A** Head and mesosoma, lateral view **B** propodeum and metasoma, lateral view **C** head and mesosoma, dorsal view **D** metanotum, propodeum and metasoma, dorsal view **E** head, frontal view **F** mesosoma, margin of the last visible sternite, ventral view.

**Plate 13.**
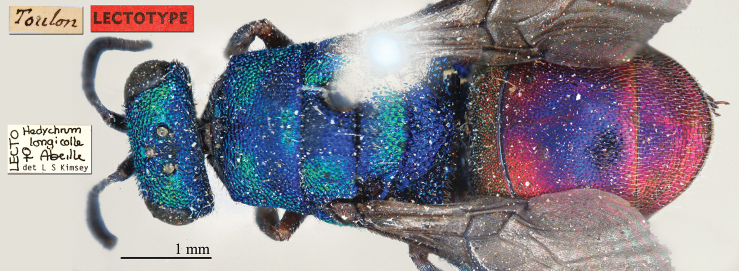
*Hedychrum longicolle* Abeille, lectotype, habitus, dorsal view.

**Plate 14.**
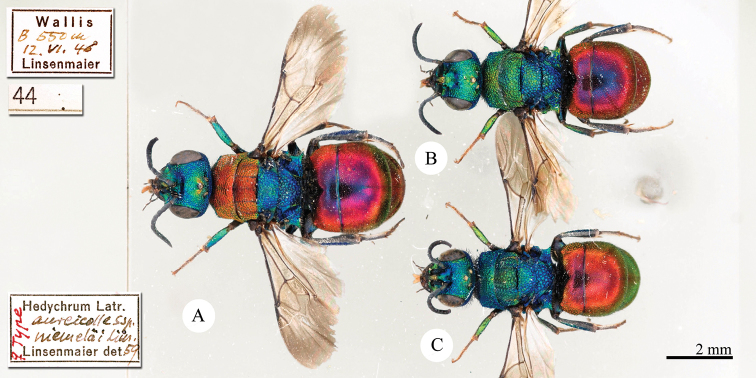
*Hedychrum niemelai* Linsenmaier, habitus, dorsal view. **A** Holotype female **B, C** paratypes, males.

**Plate 15.**
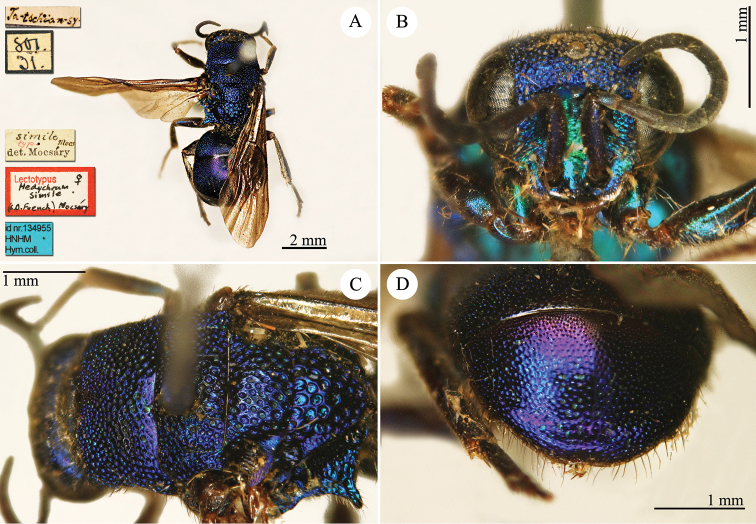
*Hedychrum simile* Mocsáry, lectotype. **A** Habitus, dorsal view **B** head, frontal view **C** mesosoma, dorsal view **D** third metasomal tergite, dorsal view.

**Plate 16.**
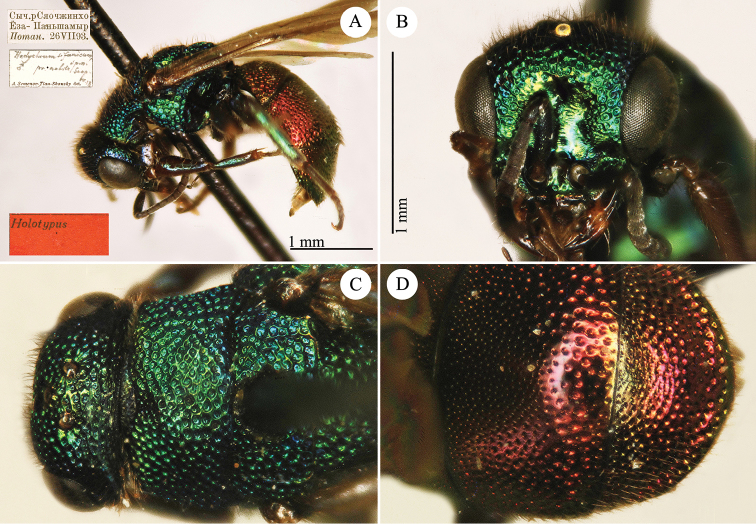
*Hedychrum sinicum* Semenov-Tian-Shanskij, holotype. **A** Habitus, lateral view **B** head, frontal view **C** mesosoma, dorsal view **D** second and third metasomal tergite, dorsal view.

**Plate 17.**
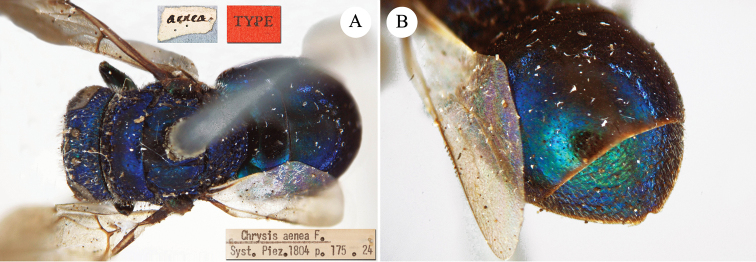
*Omalus aeneus* (Fabricius), holotype. **A** Habitus, dorsal view **B** second and third metasomal tergites, dorsal view.

**Plate 18.**
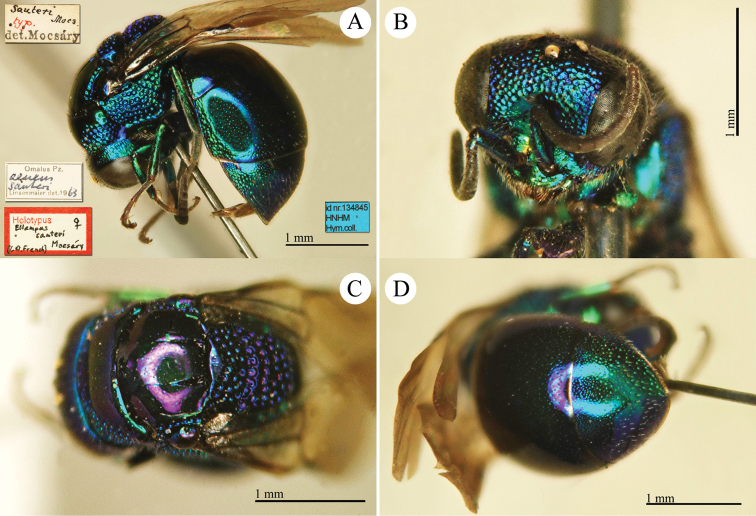
*Ellampus sauteri* Mocsáry, holotype. **A** Habitus, lateral view **B** head, frontal view **C** mesosoma, dorsal view **D** metasoma, dorsal view.

**Plate 19.**
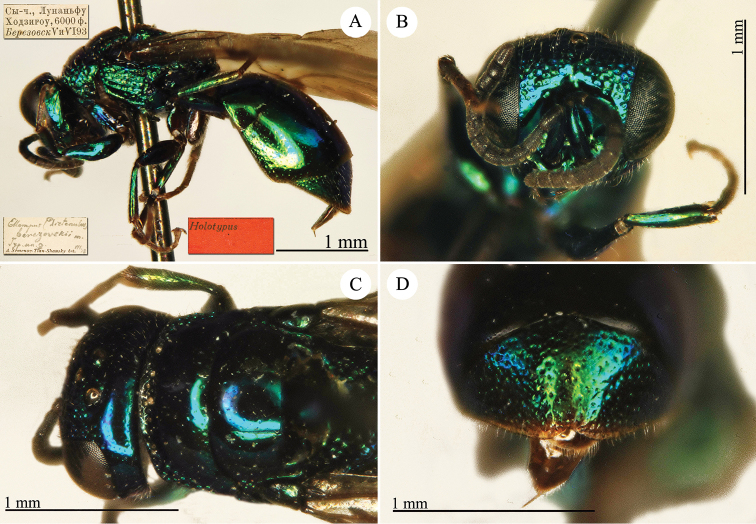
*Omalus berezovskii* (Semenov-Tian-Shanskij), holotype. **A** Habitus, lateral view **B** head, frontal view **C** head and mesosoma, dorsal view **D** third metasomal tergite, posterior view.

**Plate 20.**
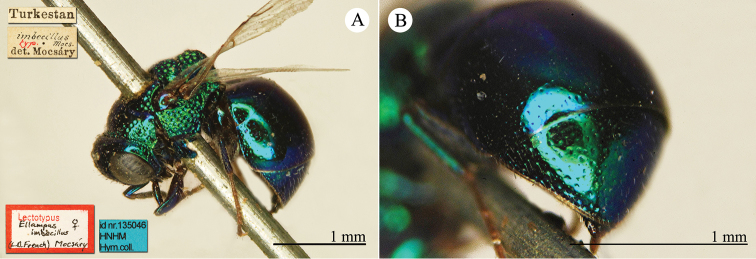
*Omalus imbecillus* (Mocsáry), lectotype. **A** Habitus, lateral view **B** second and third metasomal tergites, dorso-lateral view.

**Plate 21.**
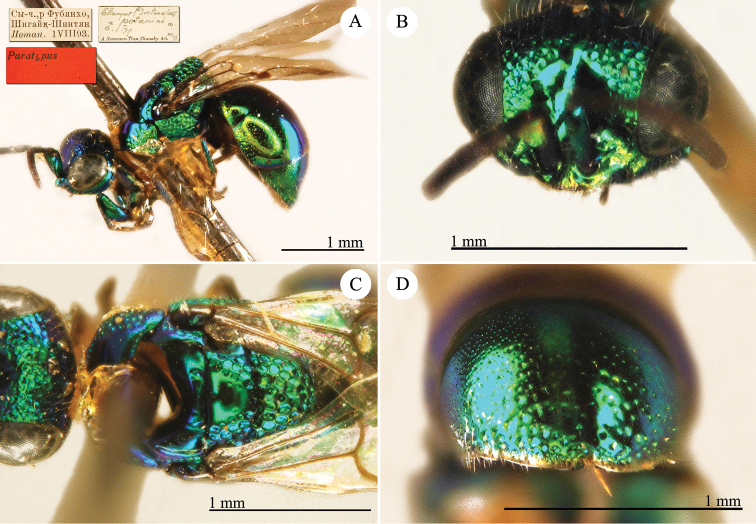
*Omalus potanini* (Semenov-Tian-Shanskij), paralectotype (?). **A** Habitus, lateral view **B** head, frontal view **C** head and mesosoma, dorsal view **D** third metasomal tergite, posterior view.

**Plate 22.**
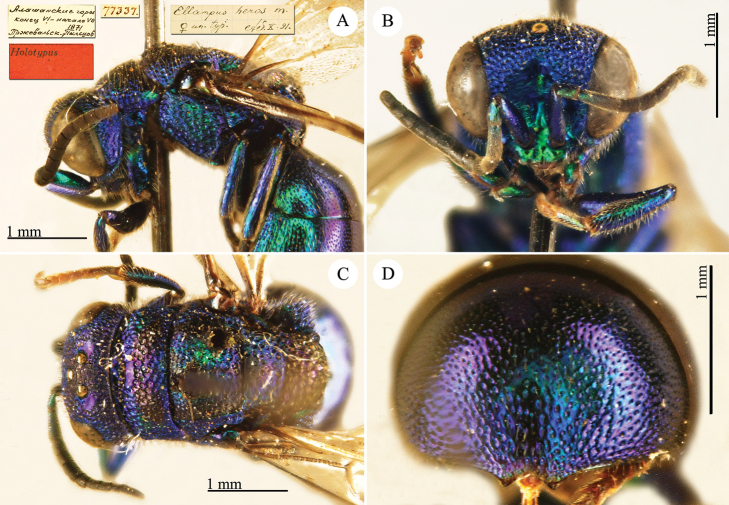
*Philoctetes heros* (Semenow), holotype. **A** Head, mesosoma and anterior part of metasoma, lateral view **B** head, frontal view **C** head and mesosoma, dorsal view **D** third metasomal tergite, posterior view.

**Plate 23.**
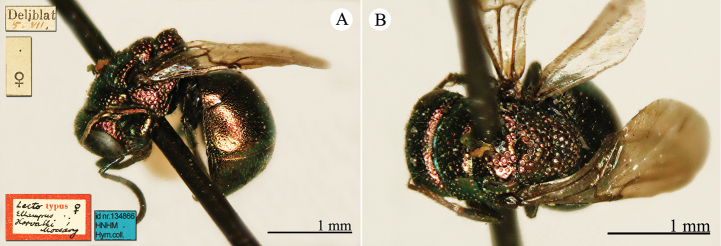
*Philoctetes horvathi* (Mocsáry), lectotype. **A** Habitus, lateral view **B** mesosoma, dorsal view.

**Plate 24.**
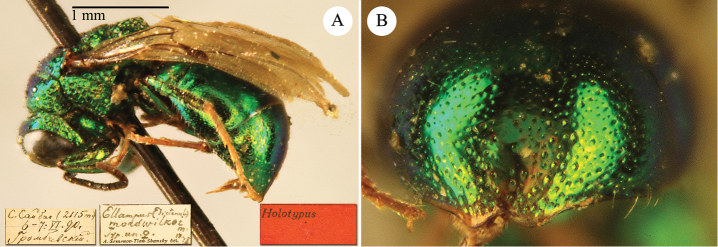
*Philoctetes mordvilkoi* (Semenov-Tian-Shanskij), holotype. **A** Habitus, lateral view **B** third metasomal tergite, dorsal view.

**Plate 25.**
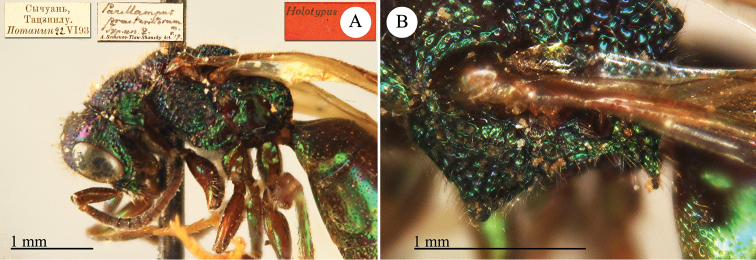
*Philoctetes praeteritorum* (Semenov-Tian-Shanskij), holotype. **A** Habitus, lateral view **B** mesopleuron and propodeum, dorso-lateral view.

**Plate 26.**
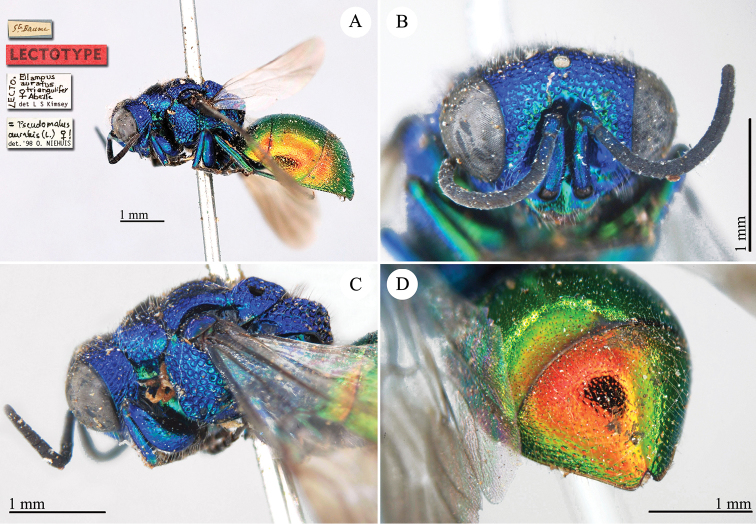
*Pseudomalus triangulifer* (Abeille), lectotype. **A** Habitus, lateral view **B** head, frontal view **C** head and mesosoma, lateral view **D** second and third metasomal tergites, dorso-lateral view.

**Plate 27.**
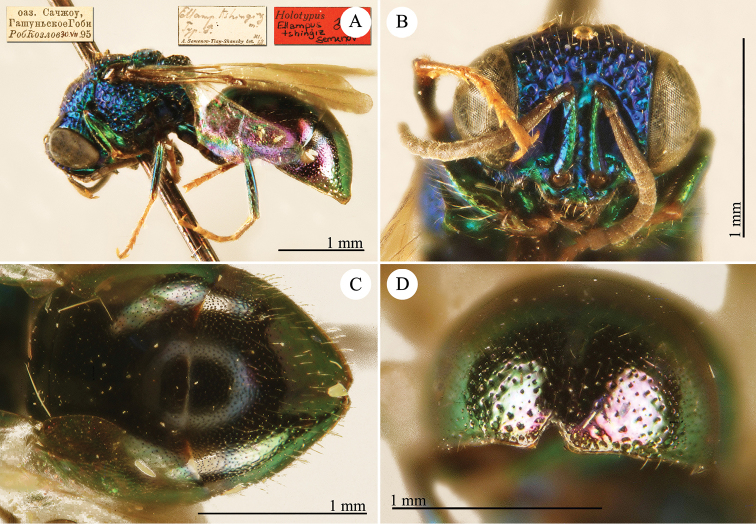
*Pseudomalus tshingiz* (Semenov-Tian-Shanskij), holotype. **A** Habitus, lateral view **B** head, frontal view **C** metasoma, dorsal view **D** third metasomal tergite, posterior view.

**Plate 28.**
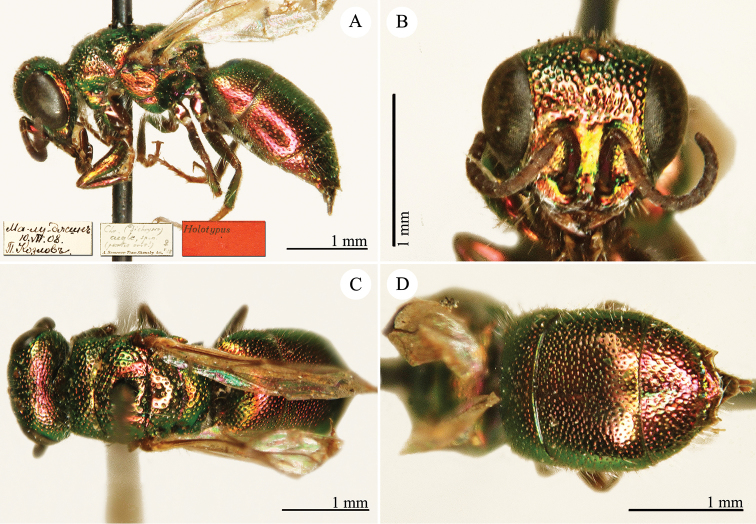
*Chrysis aegle* Semenov-Tian-Shanskij, holotype. **A** Habitus, lateral view **B** head, frontal view **C** habitus, dorsal view **D** metasoma, dorsal view.

**Plate 29.**
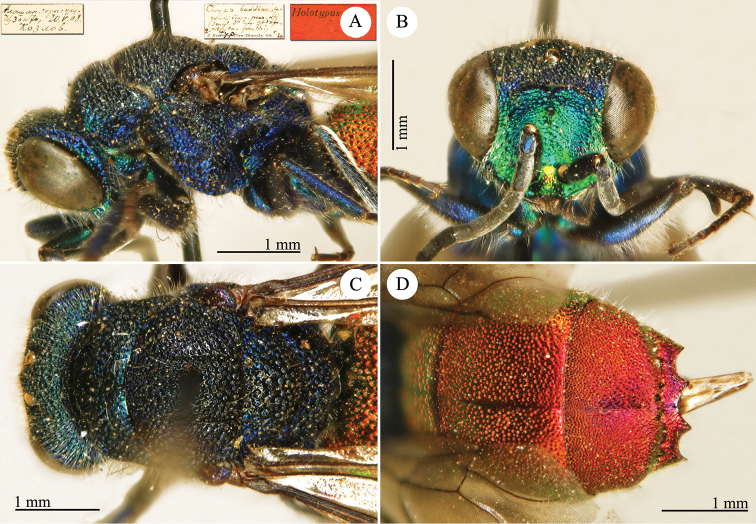
*Chrysis buda* Bohart, 1991, holotype. **A** Head and mesosoma, lateral view **B** head, frontal view **C** head and mesosoma, dorsal view **D** second and third metasomal tergites, dorsal view.

**Plate 30.**
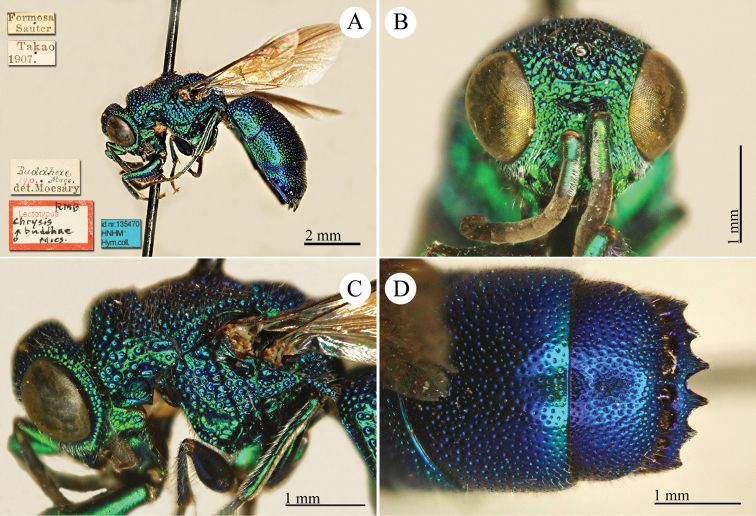
*Chrysis buddhae* Mocsáry, lectotype. **A** Habitus, lateral view **B** head, frontal view **C** head and mesosoma, lateral view **D** second and third metasomal tergites, dorsal view.

**Plate 31.**
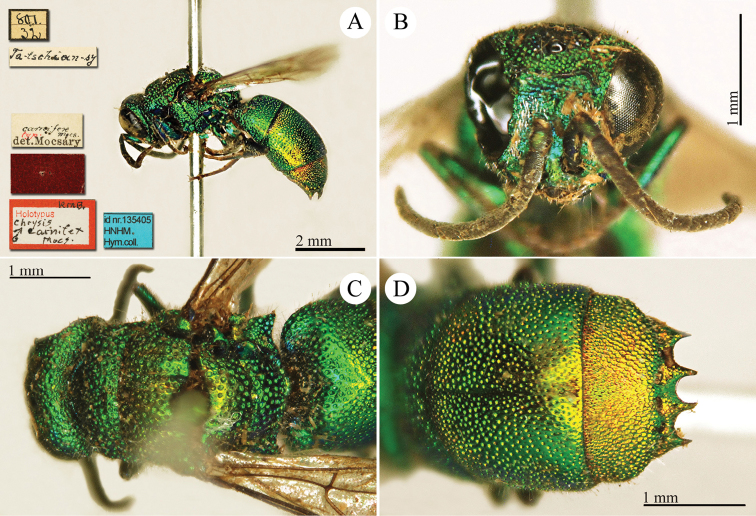
*Chrysis carnifex* Mocsáry, holotype. **A** Habitus, lateral view **B** head, frontal view **C** mesosoma, dorsal view **D** second and third metasomal tergites, dorsal view.

**Plate 32.**
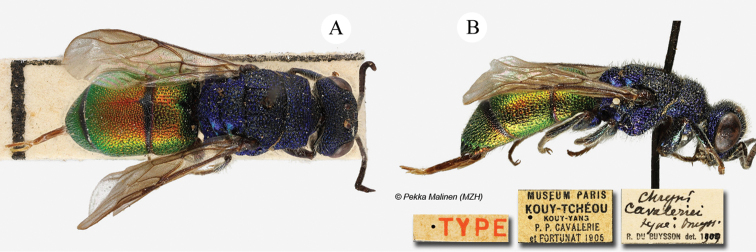
*Chrysis cavaleriei* (du Buysson), holotype. **A** Habitus, dorsal view **B** habitus, lateral view (photos courtesy of Pekka Malinen).

**Plate 33.**
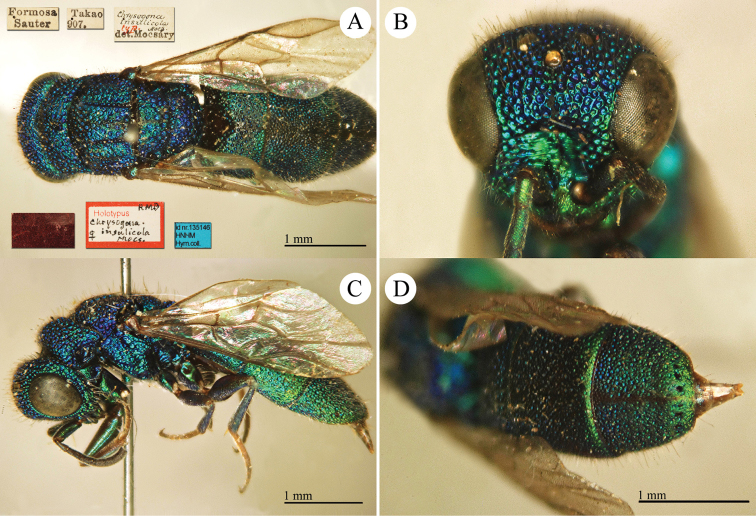
*Chrysidea insulicola* Mocsáry, holotype. **A** Habitus, dorsal view **B** head, frontal view **C** habitus, lateral view **D** second and third metasomal tergites, dorso-lateral view.

**Plate 34.**
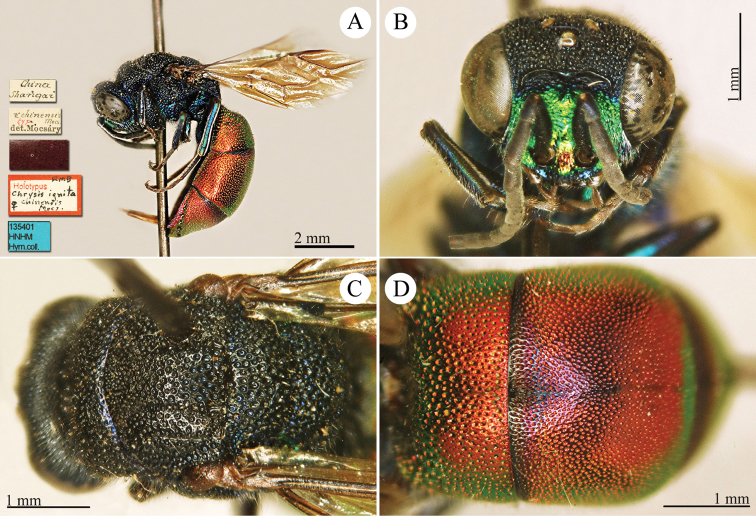
*Chrysis chinensis* Mocsáry, holotype. **A** Habitus, lateral view **B** head, frontal view **C** mesosoma, dorsal view **D** first and second metasomal tergites, dorsal view.

**Plate 35.**
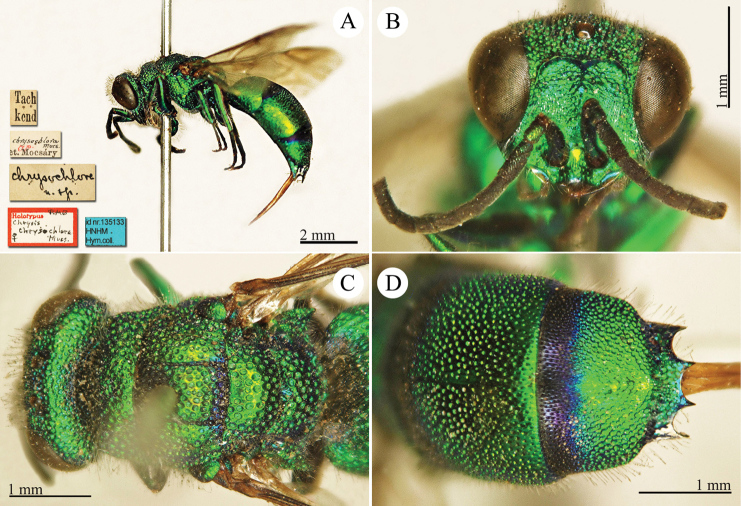
*Chrysis chrysochlora* Mocsáry, lectotype (given as holotype in the picture). **A** Habitus, lateral view **B** head, frontal view **C** head and mesosoma, dorsal view **D** second and third metasomal tergites, dorsal view.

**Plate 36.**
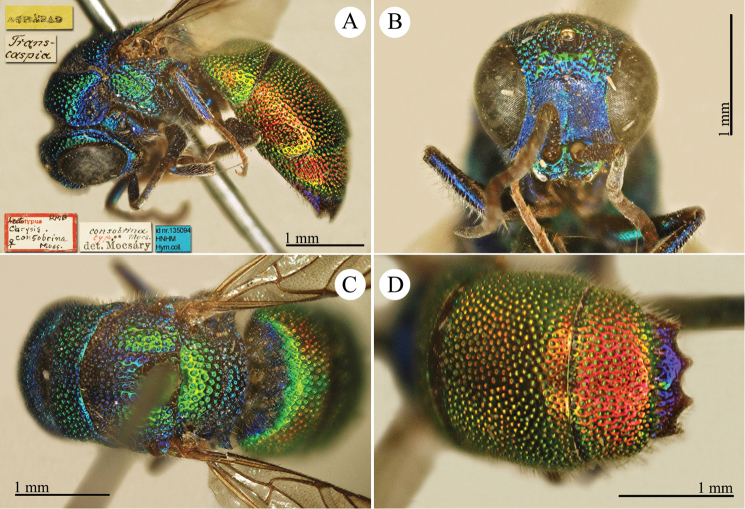
*Chrysis consobrina* Mocsáry, lectotype. **A** Habitus, lateral view **B** head, frontal view **C** mesosoma, dorsal view **D** second and third metasomal tergites, dorsal view.

**Plate 37.**
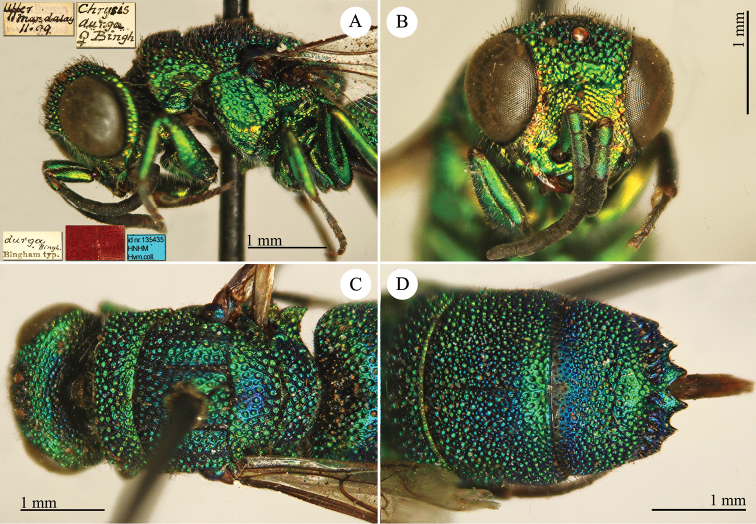
*Chrysis durga* Bingham, lectotype. **A** Head and mesosoma, lateral view **B** head, frontal view **C** mesosoma, dorsal view **D** second and third metasomal tergites, dorsal view.

**Plate 38.**
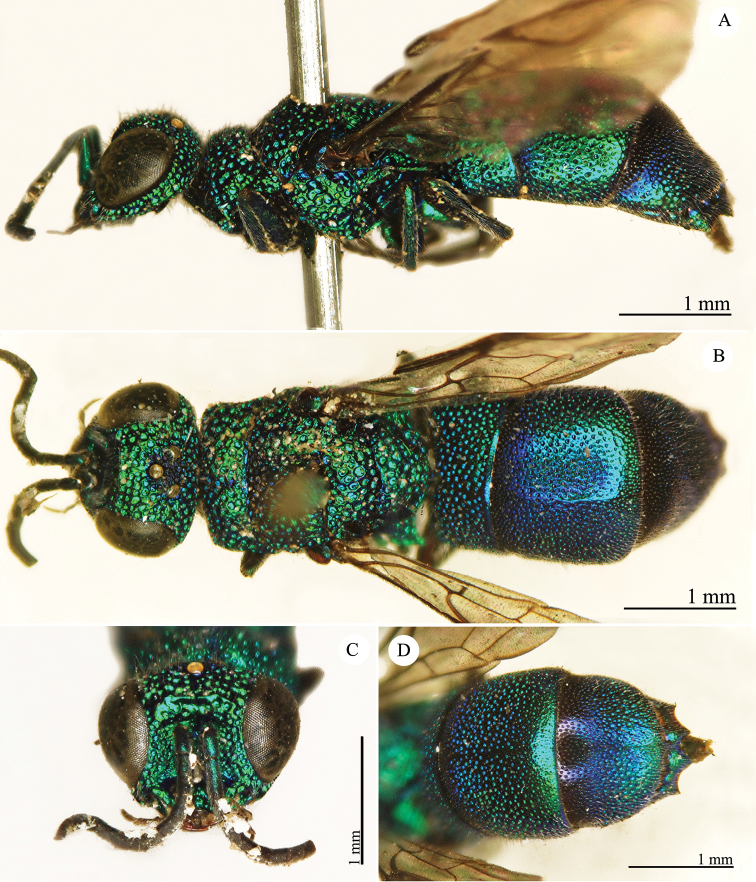
*Chrysis gracilenta* Mocsáry, holotype. **A** Habitus, lateral view **B** habitus, dorsal view **C** head, frontal view **D** second and third metasomal tergites, dorsal view.

**Plate 39.**
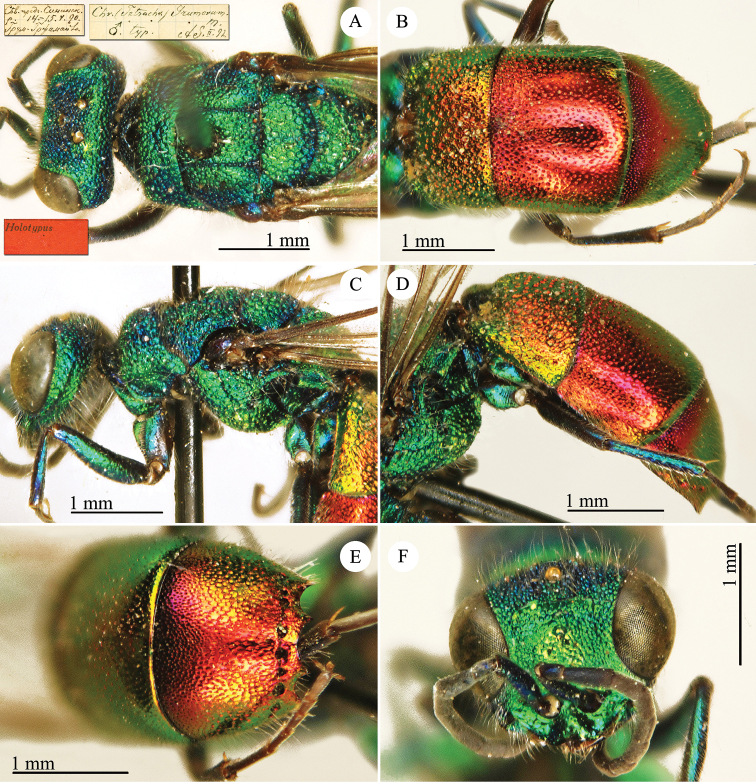
*Chrysis grumorum* Semenow, holotype. **A** Head and mesosoma, dorsal view **B** metasoma, dorsal view **C** head and mesosoma, lateral view **D** metasoma lateral view **E** third metasomal tergite, dorsal view **F** head, frontal view.

**Plate 40.**
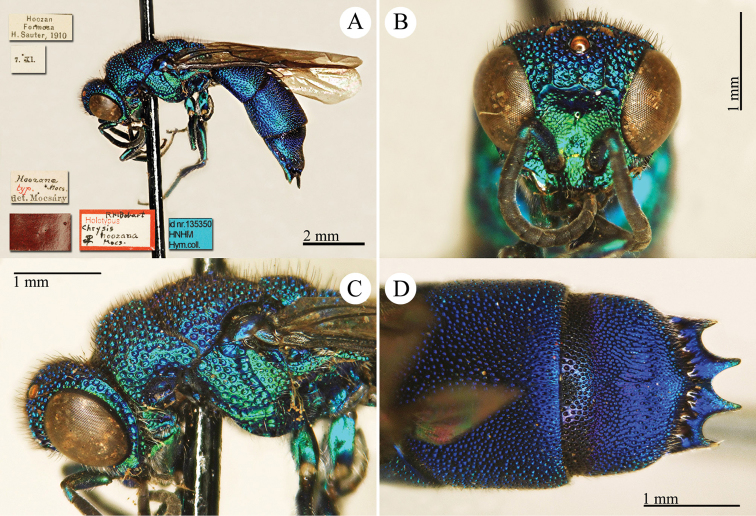
*Chrysis hoozana* Mocsáry, holotype. **A** Habitus, lateral view **B** head, frontal view **C** head and mesosoma, lateral view **D** second and third metasomal tergites, dorsal view.

**Plate 41.**
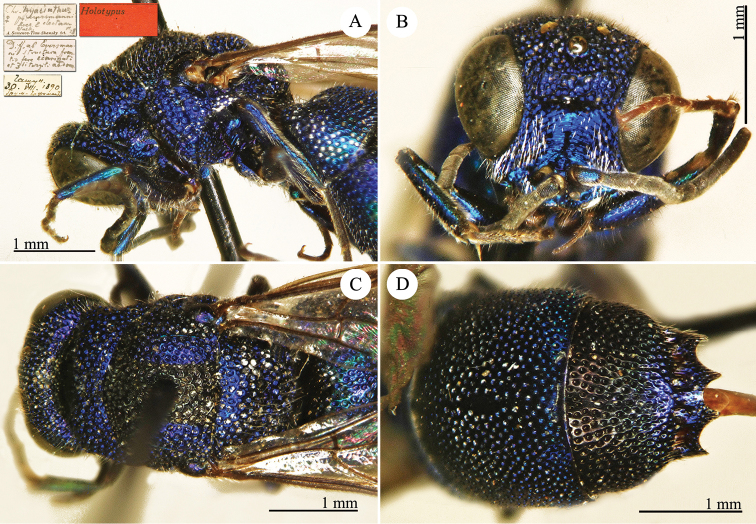
*Chrysis hyacinthus* Semenov-Tian-Shanskij, holotype. **A** Head and mesosoma, lateral view **B** head, frontal view **C** mesosoma, dorsal view **D** second and third metasomal tergites, dorsal view.

**Plate 42.**
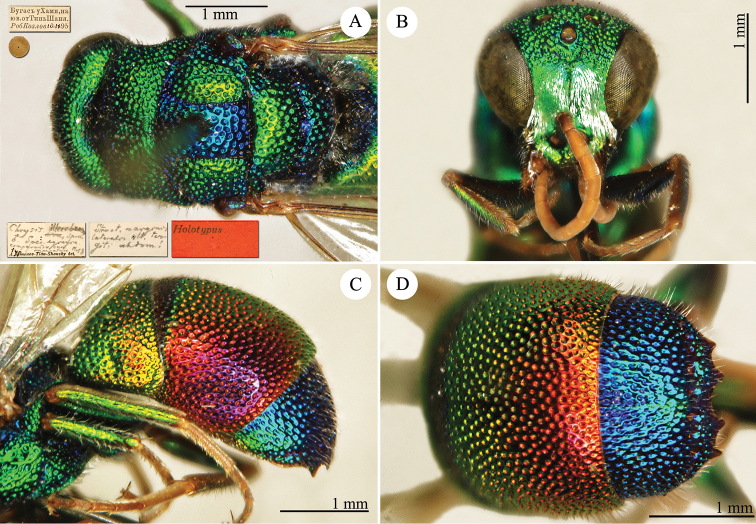
*Chrysis illecebrosa* Semenov-Tian-Shanskij, holotype. **A** Head and mesosoma, dorsal view **B** head, frontal view **C** metasoma, lateral view **D** second and third metasomal tergites, dorsal view.

**Plate 43.**
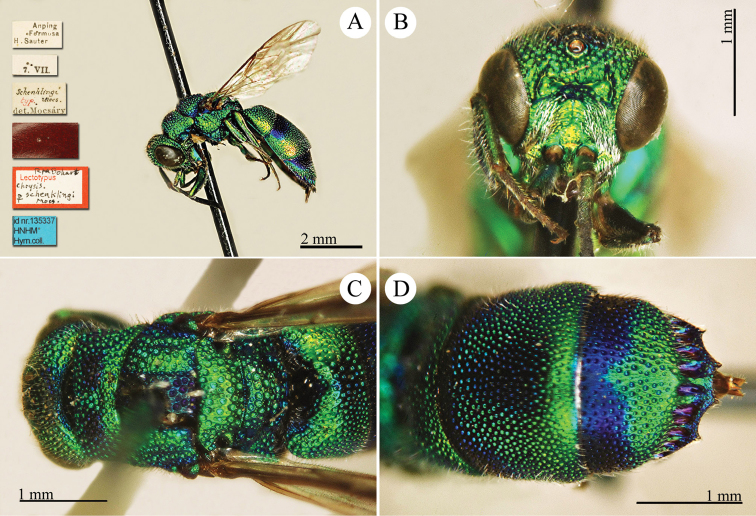
*Chrysis schenklingi* Mocsáry, lectotype. **A** Habitus, lateral view **B** head, frontal view **C** mesosoma, dorsal view **D** second and third metasomal tergites, dorsal view.

**Plate 44.**
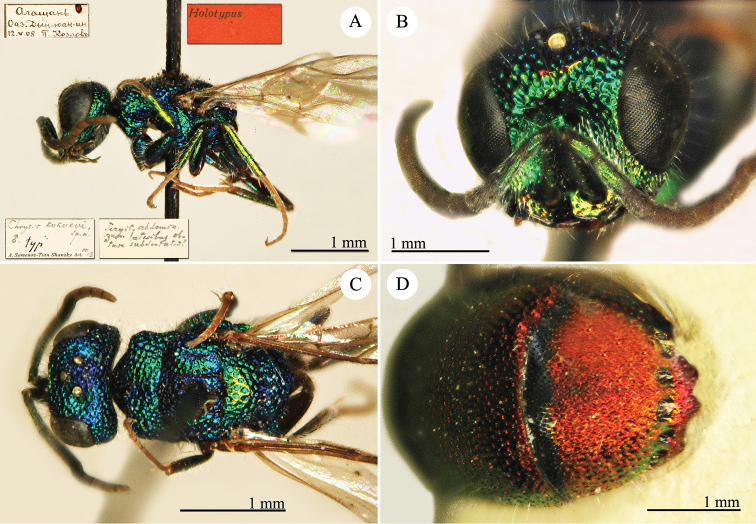
*Chrysis kokuevi* Semenov-Tian-Shanskij, holotype. **A** Head and mesosoma, lateral view **B** head, frontal view **C** head and metasoma, dorsal view **D** third metasomal tergite, dorsal view.

**Plate 45.**
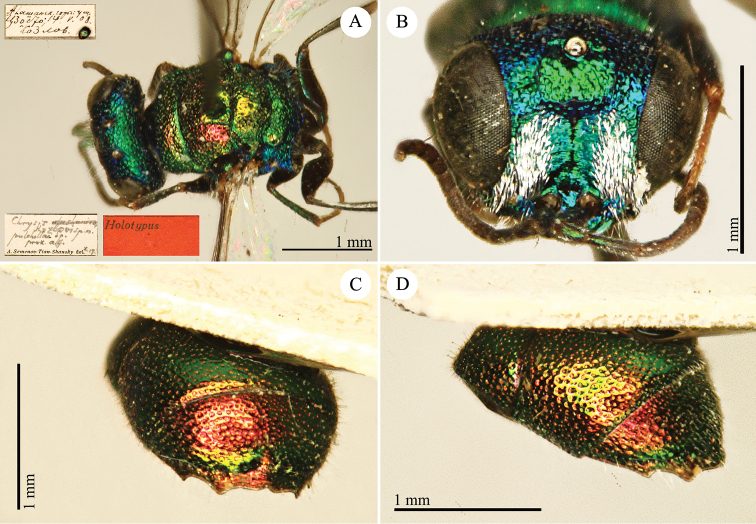
*Chrysis kozlovi* Semenov-Tian-Shanskij, holotype. **A** Head and mesosoma, dorsal view **B** head, frontal view **C** third metasomal tergite, dorsal view **D** second and third metasomal tergites, dorso-lateral view.

**Plate 46.**
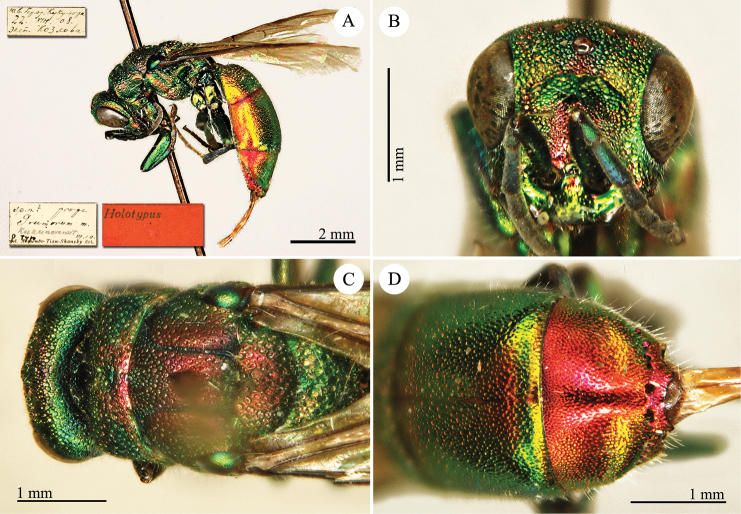
*Chrysis kukunorensis* Semenov-Tian-Shanskij, holotype. **A** Habitus, lateral view **B** head, frontal view **C** head and metasoma, dorsal view **D** second and third metasomal tergites, dorsal view.

**Plate 47.**
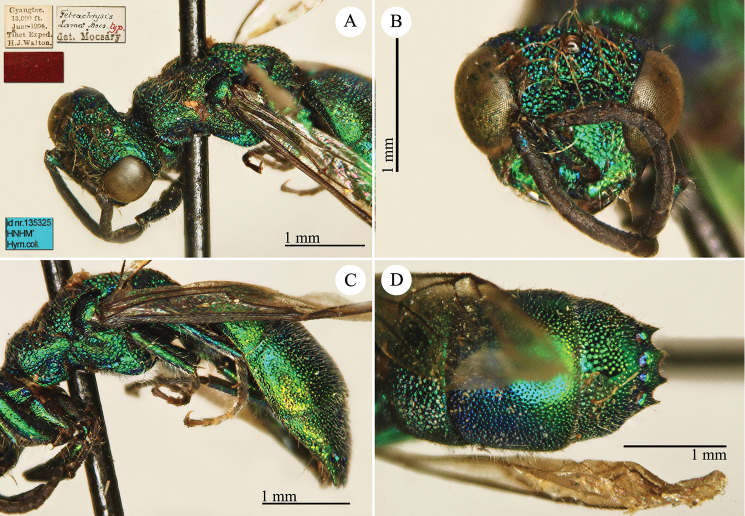
*Chrysis lama* Mocsáry, lectotype. **A** Head and mesosoma, lateral view **B** head, frontal view **C** mesosoma and metasoma lateral view **D** metasoma, dorsal view.

**Plate 48.**
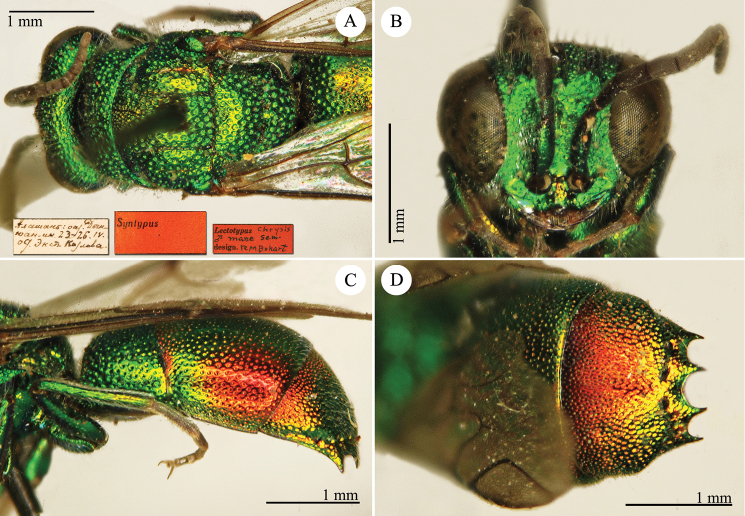
*Chrysis mane* Semenov-Tian-Shanskij, lectotype. **A** Head and mesosoma, dorsal view **B** head, frontal view **C** metasoma lateral view **D** third metasomal tergite, dorsal view.

**Plate 49.**
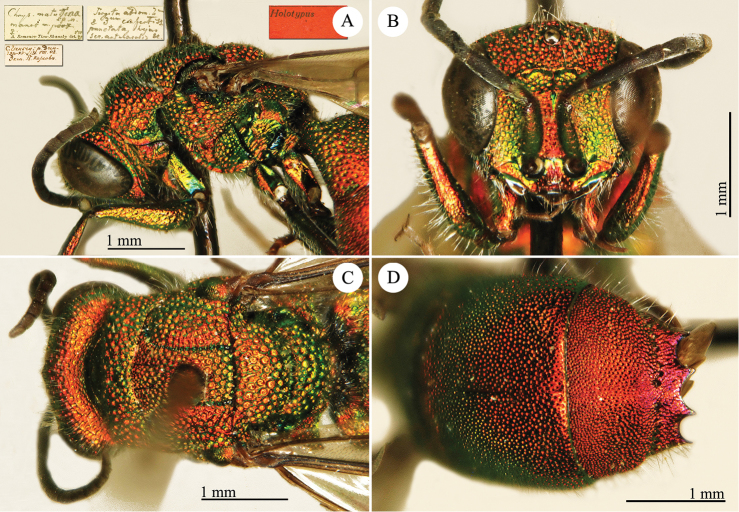
*Chrysis matutina* Semenov-Tian-Shanskij, holotype. **A** Head and mesosoma, lateral view **B** head, frontal view **C** Head and mesosoma, dorsal view **D** second and third metasomal tergites, dorsal view.

**Plate 50.**
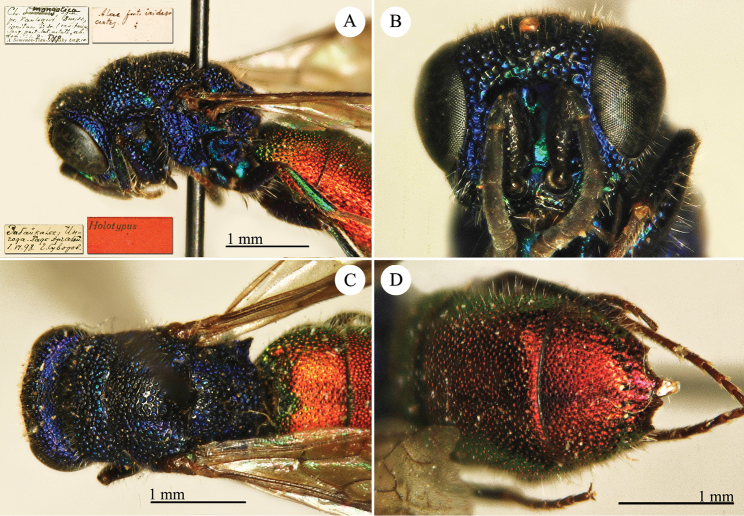
*Chrysis mongoliana* Bohart, 1991, holotype. **A** Head, mesosoma and anterior part of metasoma, lateral view **B** head, frontal view **C** head, mesosoma and anterior part of metasoma, dorsal view **D** second and third metasomal tergites, dorsal view.

**Plate 51.**
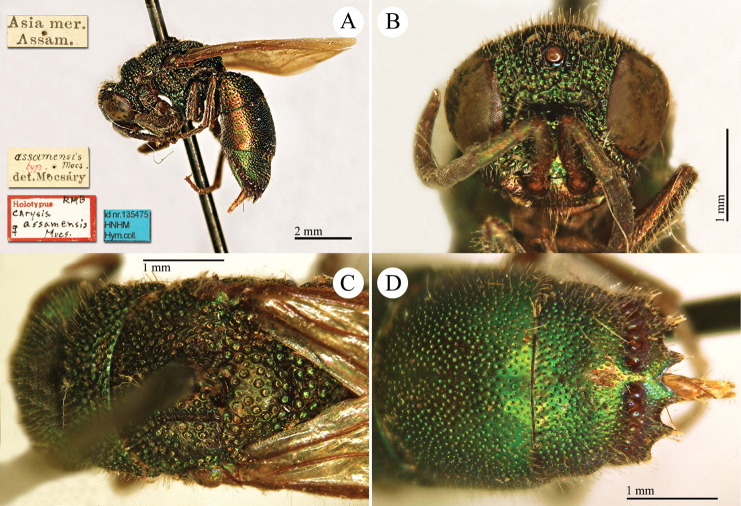
*Chrysis assamensis* Mocsáry, holotype. **A** Habitus, lateral view **B** head, frontal view **C** mesosoma, dorsal view **D** second and third metasomal tergites, dorsal view.

**Plate 52.**
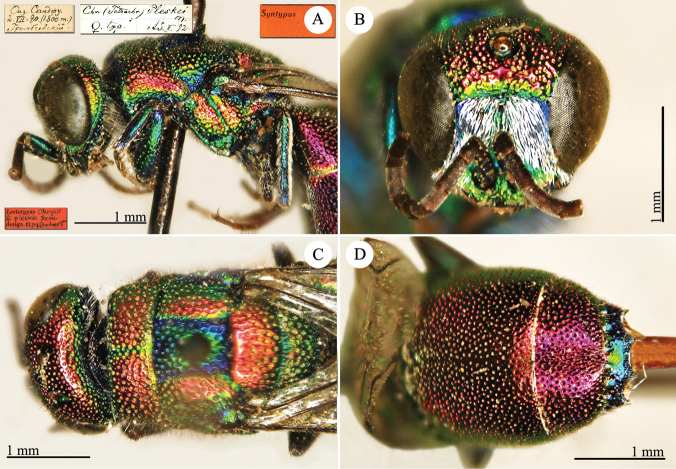
*Chrysis pleskei* Semenov-Tian-Shanskij, lectotype. **A** Head and mesosoma, lateral view **B** head, frontal view **C** Head and mesosoma, dorsal view **D** second and third metasomal tergites, dorsal view.

**Plate 53.**
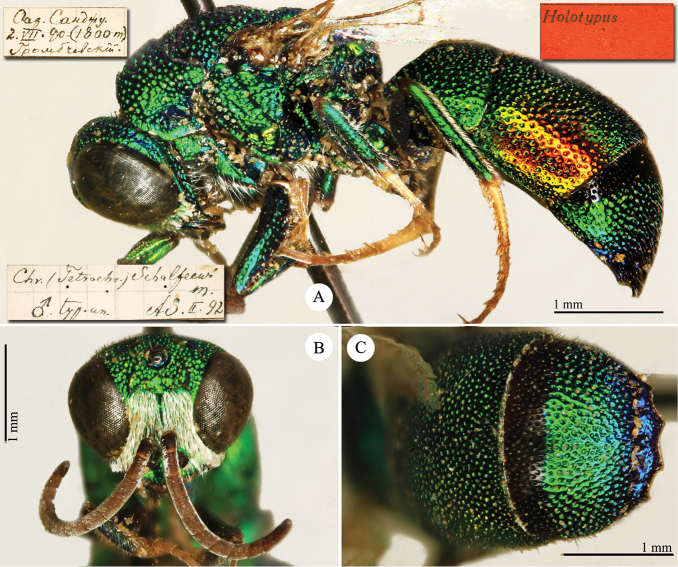
*Chrysis schalfeewi* Semenow, holotype. **A** Habitus, lateral view **B** head, frontal view **C** third metasomal tergite, dorsal view.

**Plate 54.**
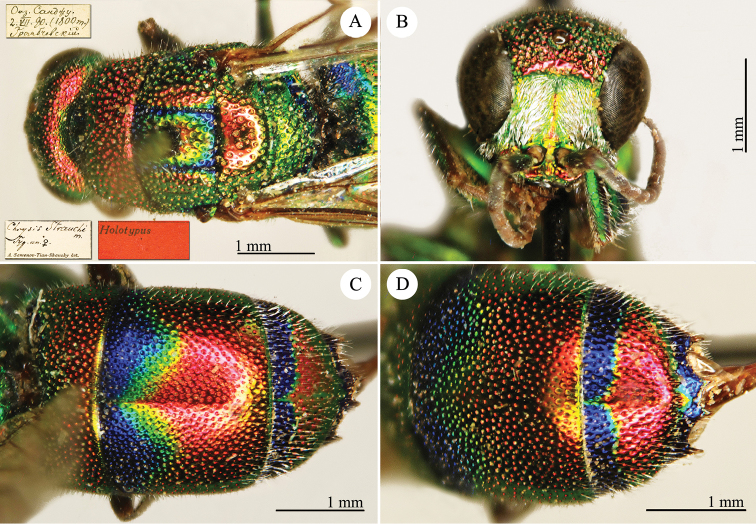
*Chrysis strauchi* Semenow, holotype. **A** Head and mesosoma, dorsal view **B** head, frontal view **C** metasoma, dorsal view **D** second and third metasomal tergites, dorsal view.

**Plate 55.**
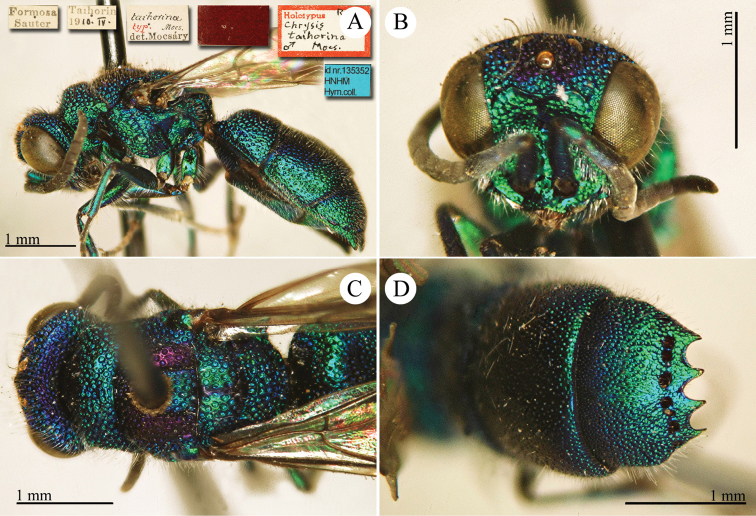
*Chrysis taihorina* Mocsáry, holotype. **A** Habitus, lateral view **B** head, frontal view **C** mesosoma, dorsal view **D** third metasomal tergite, dorsal view.

**Plate 56.**
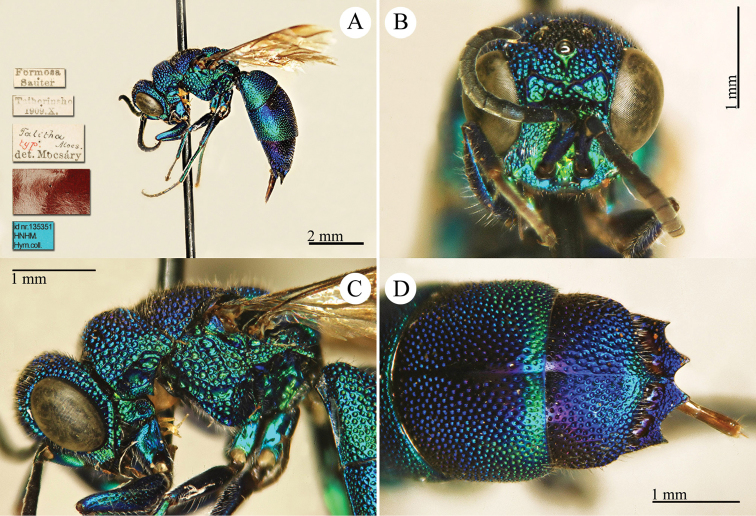
*Chrysis talitha* Mocsáry, holotype. **A** Habitus, lateral view **B** head, frontal view **C** head and mesosoma, lateral view **D** second and third metasomal tergites, dorsal view.

**Plate 57.**
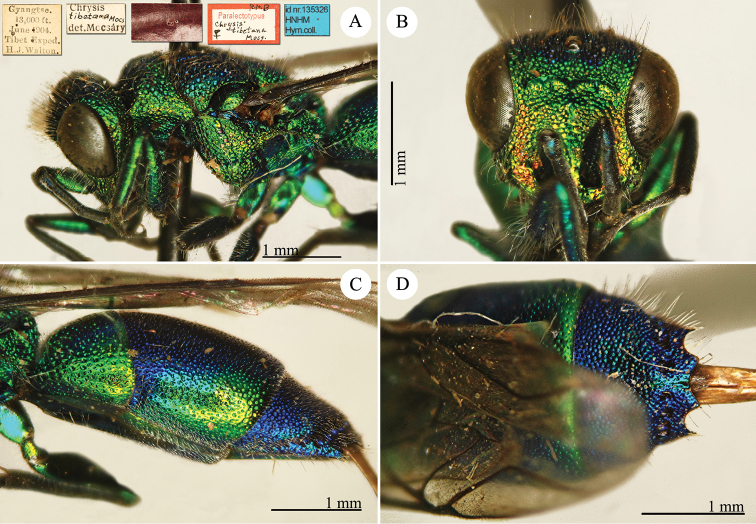
*Chrysis tibetana* Mocsáry, paralectotype. **A** Head and mesosoma, lateral view **B** head, frontal view **C** metasoma, lateral view **D** third metasomal tergite, dorsal view.

**Plate 58.**
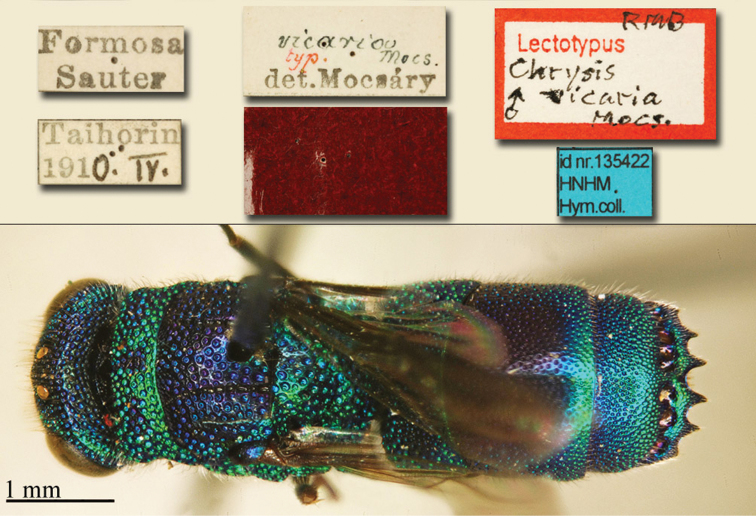
*Chrysis vicaria* Mocsáry, lectotype, habitus, dorsal view.

**Plate 59.**
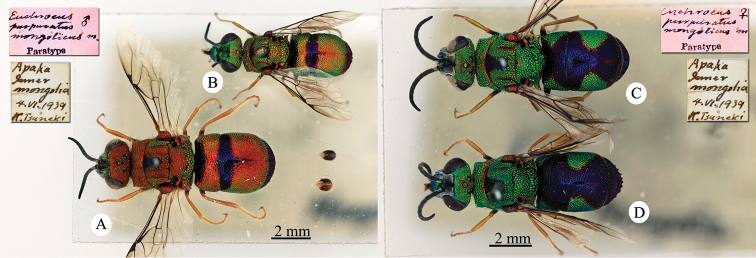
*Euchroeus mongolicus* Tsuneki, paratypes, habitus, dorsal view. **A, B** Males, dorsal view **C, D** females, dorsal view.

**Plate 60.**
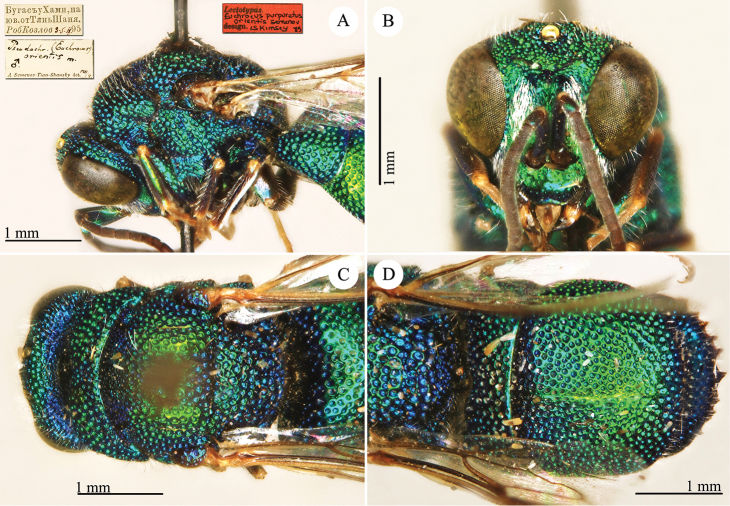
*Euchroeus orientis* (Semenov-Tian-Shanskij), lectotype. **A** Head and mesosoma, lateral view **B** head, frontal view **C** head and mesosoma, dorsal view **D** metasoma, dorsal view.

**Plate 61.**
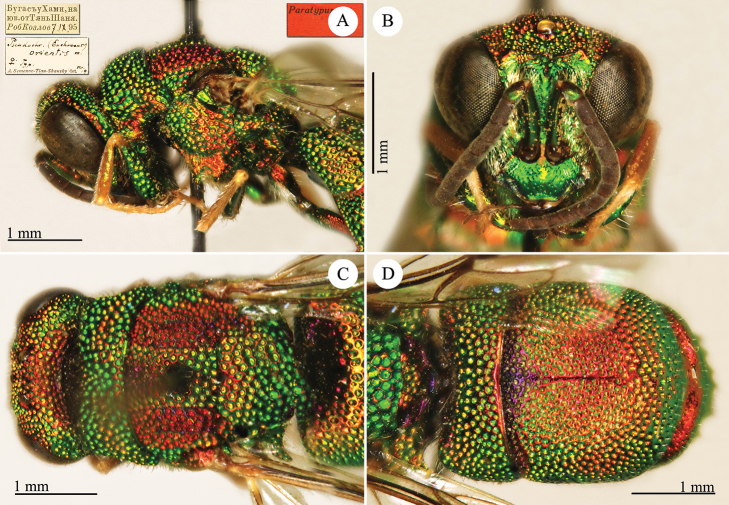
*Euchroeus orientis* (Semenov-Tian-Shanskij), paralectotype, female. **A** Head and mesosoma, lateral view **B** head, frontal view **C** head and mesosoma, dorsal view **D** first and second metasomal tergites, dorsal view.

**Plate 62.**
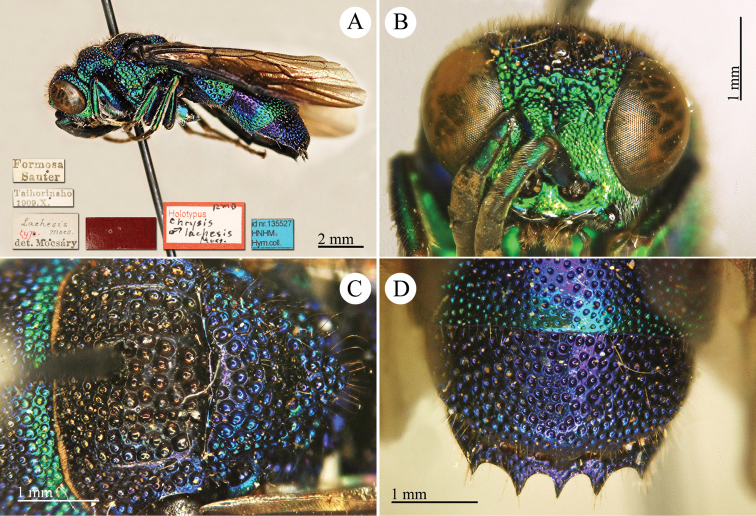
*Chrysis lachesis* Mocsáry, holotype. **A** Habitus, lateral view **B** head, frontal view **C** mesosoma, dorsal view **D** third metasomal tergite, dorsal view.

**Plate 63.**
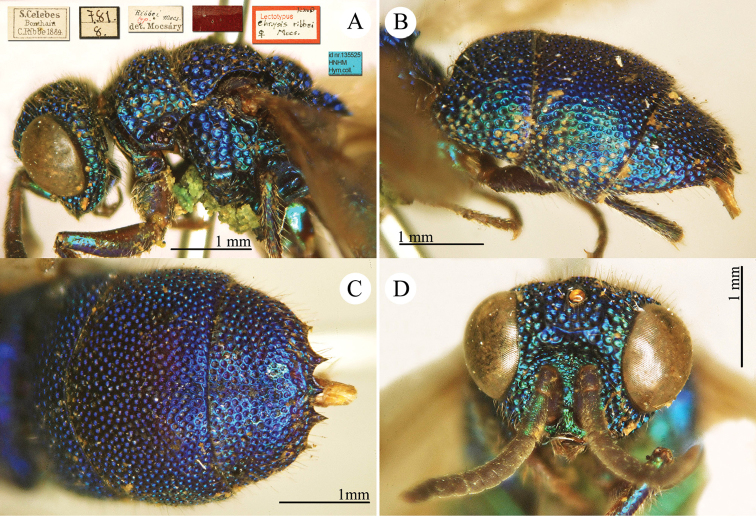
*Praestochrysis ribbei* (Mocsáry, 1889), lectotype. **A** Head and mesosoma, dorsal view **B** metasoma, lateral view **C** second and third metasomal tergites, dorsal view **D** head, frontal view.

**Plate 64.**
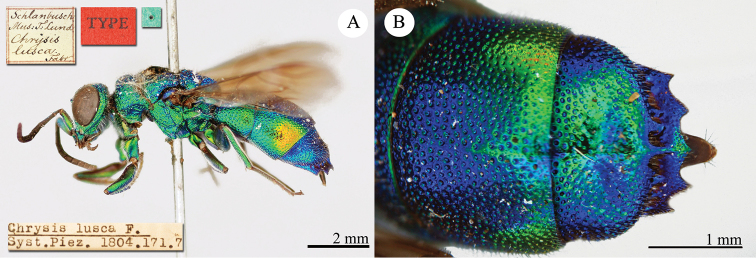
*Trichrysis lusca* (Fabricius), holotype. **A** Habitus, lateral view **B** second and third metasomal tergites, dorsal view.

**Plate 65.**
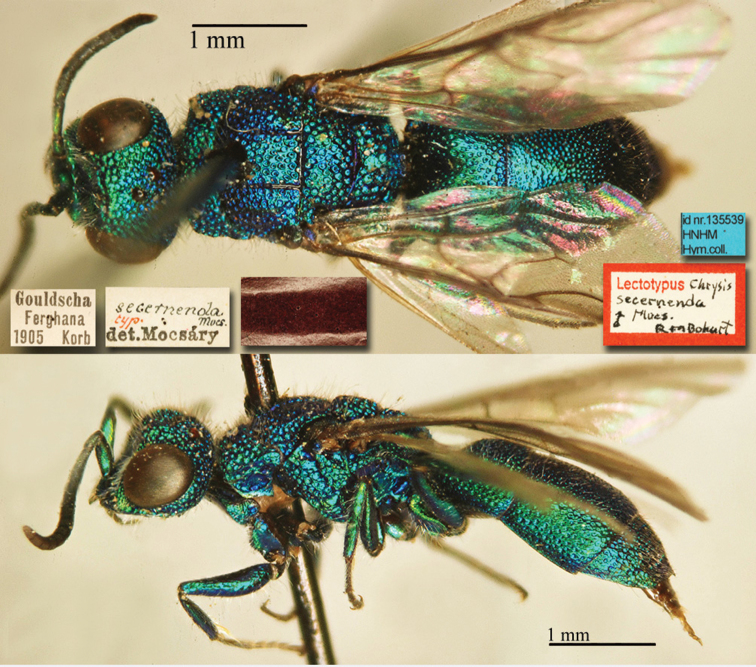
*Trichrysis secernenda* (Mocsáry), lectotype. **A** Habitus, dorsal view **B** habitus, lateral view.

**Plate 66.**
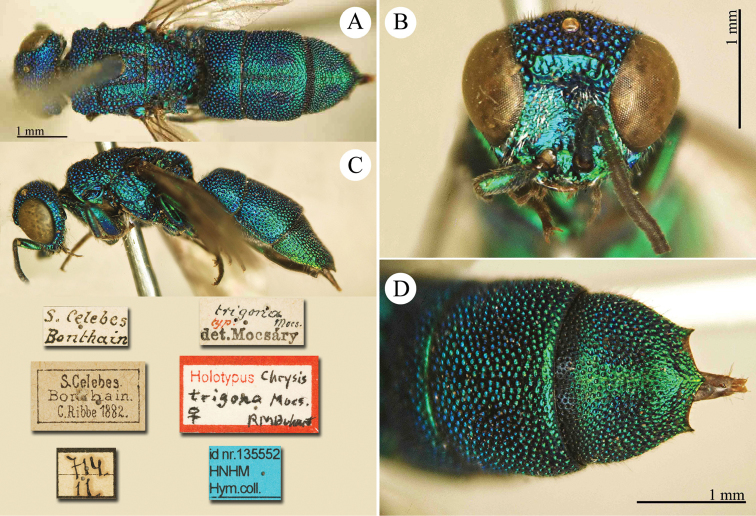
*Trichrysis trigona* (Mocsáry), holotype. **A** Habitus, dorsal view **B** head, frontal view **C** habitus, lateral view **D** second and third metasomal tergites, dorsal view.

## II. Taxa to be excluded from China

**1. *Chrysis coerulans* Fabricius, 1804**

**Remarks.**

Radoszkovsky (1866) identified two specimens as *Chrysis coerulans* Fabricius, but it is a misidentification, as *Chrysis coerulans* is a Nearctic species (currently *Chrysis nitidula* Fabricius, 1775). Radoszkovski was not sure about his identification: “J’ai placé cette espèce sous le nom de *coerulans*; quoique plus rapprochée par la couleur de cette dernière espèce, elle ressemble en même temps par sa forme à la *chrysis nitidula*”. Dalla Torre (1892, sub *caerulans* Fabr.) reported the same datum for China.

**2. *Chrysis syriaca* Guérin-Méneville, 1842**

**Remarks.**

Bischoff (1913: 51) erroneusly listed *Chrysis nomima* du Buysson (currently *Chrysis syriaca*) as being from China, rather than from Algeria and Egypt.

**3. *Holopyga gloriosa viridis* Guérin-Méneville, 1842**

**Remarks.**

*Holopyga viridis* is present from North Africa to Palestine (Linsenmaier 1959, 1999) with records also found further west towards Oman (Linsenmaier 1994). Its appearance in China is dubius and the specimens identified by Tsuneki (1947, 1948a) must be double-checked.

## III. Doubtful taxa mentioned in China

**1. *Cleptes nitidulus* (Fabricius, 1793)**

**Remarks.**

The report of *Chrysis nitidulus* in the east Palaearctic (Uchida 1926) was considered debatable by Ha et al. (2011). Therefore, *Chrysis nitidulus* is temporarily excluded from the checklist of the Chinese *Cleptes*.

**2. *Cleptes semiauratus* (Linnaeus, 1758)**

**Remarks.**

As in the previous case, Sheng et al. (1998) reported *Chrysis semiauratus* as a new record to China. After examining the specimens, Wei et al. (2013) discovered that the specimen evidently belonged to an undescribed species (*Chrysis shengi* Wei, Rosa and Xu, 2013). However *Chrysis semiauratus* may be present in the most western part of the country.

**3. *Hedychrum coerulescens* Shuckard, 1837**

**Remarks.**

Tsuneki (1946, 1947, 1948a) listed *Hedychrum coerulescens* Schuckard, 1837 *nec* Lepeletier, 1806, *nec* Chevrier, 1862. The occurence of this species in China is surely in error. Tsuneki based the identification on a very short diagnosis of two specimens housed in BMNH without locality labels. At present these species cannot be recognized and the description is not adequate to identify the species. Furthermore, the name *Hedychrum coerulescens* Schuckard is a primary junior homonym of *Hedychrum coerulescens* Lepeletier, 1806 (currently *Pseudomalus violaceus* (Scopoli, 1763)).

**4. *Holopyga amoenula amoenula* Dahlbom, 1854**

**Remarks.**

The presence of *Holopyga amoenula amoenula* Dahlbom, 1854 (sub *Holopyga gloriosa amoenula* in du Buysson (1911) and Tsuneki (1948a)) is restricted to the island of Rhodes. The Chinese records identified by du Buysson and Tsuneki must be double-checked so as to confirm their identity.

**5. *Loboscelidia defecta* Kieffer, 1916**

**Remarks.**

It was listed from south China by Kimsey and Bohart (1991), but was recently excluded from the Chinese fauna by Kimsey (2012).

**6. *Pseudomalus bergi* (Semenov-Tian-Shanskij, 1932)**

**Remarks.**

Kimsey and Bohart (1991) mistakenly listed *bergi* as having originally described from China, but the correct locality is “Prov. Heptapotamica (Semiretshj’e); vallis fl. Kora in montibus Alatau Dzungarico”. “Dzungarian Alatau” is a mountain range found along the boundary between Dzungaria (China) and Zhetysu (Kazakhstan), however the Kora river is located within the former Soviet country. Regardless, the species may be present in China too.

**7. *Pseudomalus pusillus* (Fabricius, 1804)**

**Remarks.**

Listed in China by Berland and Bernard (1938: 35, Shanxi: Nan-chan) and by Balthasar (1954) without any precise locality.

## Conclusion

The current number of known Chinese chrysidid species and subspecies is 188 in total. We excluded some published yet doubtful data due to uncertain identifications. Overall, the Chinese chrysidid fauna is still poorly known, comparing with the fauna of the adjacent countries both in the Palearctic Region (e.g. Korea, Mongolia, Far East Russia, Tajikistan) and the Oriental Region (e.g. Burma, Vietnam). We expect a higher number of taxa for China as results of its geographical position, climatic condition and topographical structure.
